# Safety and broad immunogenicity of HIVconsvX conserved mosaic candidate T-cell vaccines vectored by ChAdOx1 and MVA in HIV-CORE 006: a double-blind, randomised, placebo-controlled phase 1 trial in healthy adults living without HIV-1 in eastern and southern Africa

**DOI:** 10.1016/j.lanmic.2024.101041

**Published:** 2025-06

**Authors:** Chama Chanda, Freddie Kibengo, Michael Mutua, Fred Ogada, Vincent Muturi-Kioi, Belkis M Akis Yildirim, Mary Amondi, Andrea Baines, Vincent Basajja, Nicola Borthwick, Kefa Bosire, Elias Chambula, Paramesh Chetty, Kundai Chinyenze, Oscar Chirro, Alison Crook, Jan De Bont, Natalia Fernandez, Peter Ejou, Bashir Farah, Molly Glaze, Ben Gombe, Anne Gumbe, Peter Hayes, Sally Itwi, Sheba Juma, Anita Kabarambi, Chishiba Kabengele, Paddy Kafeero, Ayoub Kakande, Jennifer Kanungi, William Kidega, Deborah King, Rose Mahira, Roselyn Malogo, Mabela Matsoso, Clive Michelo, Annie Moyo, Susan Mugaba, Irene Mugenya, Patrick Muhumuza, Yama F Mujadidi, Moses Muriuki, Vernon Musale, Gaudensia Mutua, Meya Muwowo, Fatima Mwale, Irene Mwangi, Maria Nakimbugwe, Angella Namuyanja, Eunice Nduati, Leslie Nielsen, Jaquelyn Nyange, Geofrey Oino, Brenda Okech, Gloria Omosa-Manyonyi, Dan Otieno, Shaun Palmer, Hilda Phiri, Kelly Ramko, Rachel L Rutishauser, Eddy Sayeed, Rose Sajabi, Jennifer Serwanga, Edmund G-T Wee, Claire Wenden, Paola Cicconi, Patricia Fast, Jill Gilmour, Walter Jaoko, Pontiano Kaleebu, William Kilembe, Hester Kuipers, Eduard J Sanders, Tomáš Hanke

**Affiliations:** a Center for Family Health Research Zambia (CFHRZ), Lusaka, Zambia; b Division of Experimental Medicine, Zuckerberg San Francisco General Hospital, University of California, San Francisco, CA, USA; c IAVI, New York, NY, USA; d IAVI Human Immunology Laboratory, Imperial College London, London, UK; e Joint Research Center for Human Retrovirus Infection, Kumamoto University, Kumamoto, Japan; f KEMRI–Wellcome Trust Research Programme, Kilifi, Kenya; g KAVI Institute of Clinical Research (KAVI-ICR), University of Nairobi, Nairobi, Kenya; h Medical Research Council/Uganda Virus Research Institute and London School of Hygiene & Tropical Medicine Uganda Research Unit, Entebbe, Uganda; i Oxus Technologies, Oxford, UK; j The Jenner Institute, Nuffield Department of Medicine, Oxford University, Oxford, UK; k UVRI-IAVI HIV Vaccine Program, Entebbe, Uganda

## Abstract

**Background:**

Even within the context of antiretroviral treatment and prevention, an HIV-1 vaccine remains the best strategy for ending the HIV/AIDS epidemic. A vaccine is particularly needed in sub-Saharan Africa, where HIV-1 greatly affects people's lives and economy. Here, we aimed to assess the safety and immunogenicity of candidate T-cell vaccines in African populations.

**Methods:**

HIV-CORE 006 was a double-blind, randomised, placebo-controlled phase 1 trial conducted across four clinical research centres in Uganda, Kenya, and Zambia. Eligible participants were not pregnant, were living without HIV-1 or HIV-2, had a low likelihood of acquiring HIV-1, were aged 18–50 years, fully comprehended the purpose and details of this study as outlined in the participant information sheet, and passed an assessment of understanding before providing written informed consent. Participants were randomly assigned (9:2) to receive either a vaccine regimen or a placebo. The vaccine was administered as ChAdOx1.tHIVconsv1 (C1) followed by MVA.tHIVconsv3 (M3) and MVA.tHIVconsv4 (M4) in regimen C1-M3M4. The first primary outcome was the vaccines’ safety assessment, assessed in all participants who received at least one vaccine or placebo dose. The second primary outcome evaluated the C1-M3M4 regimen’s induction of HIVconsvX-specific T-cell responses by assessing the proportion of vaccine recipients who responded to the vaccination, assessed in all participants who received all doses of vaccine or placebo as per protocol. This study is registered with ClinicalTrials.gov, NCT04553016, and the Pan-African Clinical Trials Registry PACTR202006495409011, and is now closed.

**Findings:**

Between July 15, 2021, and Nov 2, 2022, 89 healthy adults living without HIV-1 were randomly assigned, with 88 receiving either the vaccine (n=72) or placebo (n=16). Of these 88 participants, 57 (65%) were male and 31 (35%) were female. The C1, M3, and M4 vaccine components were well tolerated and induced HIVconsvX-specific responses in 70 (99%) of the 71 participants who completed all vaccine doses. Vaccine-elicited T cells peaked at a median of 2310 (IQR 1080–4480) IFN-γ spot-forming units per 10^6^ peripheral blood mononuclear cells and recognised a median of eight (five to ten) of ten peptide pools spanning the HIVconsvX immunogen. The total frequencies of elicited T cells decreased 4·6 times over a 40-week follow-up period compared with the peak responses. Upon antigenic re-exposure, T cells proliferated, exhibited multiple effector functions, and inhibited HIV-1 representatives from clades A, B, C, and D.

**Interpretation:**

Results from key sub-Saharan African populations supported the safety of the vaccine regimen previously shown in the first-in-human trial in the UK. The induction of T cells and their characteristics encourage vaccine integration into HIV-1 cure strategies, which could inform HIV-1 prevention efforts.

**Funding:**

The European and Developing Countries Clinical Trials Partnership.

## Introduction

HIV-1 acquisition and AIDS remain a global public health crisis. Despite substantial progress in prevention and treatment, approximately 39 million people worldwide are living with HIV-1. In 2022 alone, there were 1·3 million new virus acquisitions and 630 000 individuals died from AIDS-related illnesses.^1^ These figures are unacceptably high and highlight the urgent need for an effective HIV-1 vaccine. Even in the most affected regions such as sub-Saharan Africa, prevention options are frequently not readily available. Various preventive methods exist, such as condom use, pre-exposure prophylaxis, and medically supervised male circumcision. However, these methods are not universally effective or accessible for everyone and populations without access to these preventive measures remain vulnerable. Additionally, HIV-1/AIDS imposes a major economic and social burden on individuals with HIV-1 and their families, communities, and health-care systems. Although combination antiretroviral therapy has substantially reduced HIV-1 fatalities, transforming AIDS into a manageable chronic infection, treatment is not a cure. Lifelong medications entail side-effects and pose adherence challenges and stigma. Considerable efforts have been made to develop an HIV-1 vaccine, but progress has proven scientifically challenging, slow, and fraught with setbacks. Thanks to the perseverance of dedicated teams in the HIV-1 vaccine research community, there is now renewed optimism supported by emerging data suggesting that current efforts are advancing in the right direction towards an efficacious HIV-1 vaccine.^2^, ^3^, ^4^

**Research in context**

**Evidence before this study**

Our working hypothesis postulates that focusing vaccine-elicited killer T cells on the most functionally conserved regions of the HIV-1 proteome will slow HIV-1 escape and efficiently eliminate virus-producing cells. CD8^+^ killer T cells can inhibit HIV-1 replication if the virus carries epitopes that these T cells recognise. Yet, in most individuals, even the robust T-cell responses generated by the initially healthy immune system during acute HIV-1 infection are unable to halt the virus due to escape mechanisms facilitated by prolific mutations. It is, therefore, important to recognise that not all T-cell responses provide equal protection, and it follows that the aim of vaccines is to induce more effective T cells than those elicited by natural infection. For challenging microbes that have long resisted vaccine development, such as HIV-1, even the successful induction of broadly neutralising antibodies—a key goal of HIV-1 vaccinology—will benefit from the safety net of protective T-cell responses in controlling HIV-1 viraemia and preventing progression to AIDS. It remains a real possibility that past unsuccessful vaccine candidates as well as the marginally effective RV144 vaccine tested for the prevention of HIV-1 acquisition could have shown more favourable outcomes had they induced more effective CD8^+^ killer T cells. Reports on efficacy trials evaluating experimental interventions to prevent HIV-1 acquisition published on PubMed as of Aug 1, 2024 were considered.

**Added value of this study**

Sub-Saharan Africa bears the greatest global burden of AIDS-related deaths and new HIV-1 acquisitions. Participation and eventual acceptance of any HIV-1 intervention in the region will be most effective if novel vaccine candidates are tested in this population. Thus, it is crucial to engage African scientists and wider communities in the development and design of clinical trials to enhance and strengthen the region’s research capacity, and later for scaling up using the network of adequately developed clinical research centres (CRCs) for the region. This strategy will foster trust in any future vaccine that emerges from such studies. Testing of the HIVconsvX vaccines in HIV-CORE 006 and capacity building were two objectives of the Globally Relevant HIV Vaccine Europe-Africa Clinical Trial (GREAT) consortium. The four CRCs of HIV-CORE 006 were selected to encompass regions with diverse circulating HIV-1 clades A and D (in Kenya and Uganda) and clade C (in Zambia). The trial enrolled a larger number of participants than the first-in-human trial conducted in the UK with prevalent clade B testing the same vaccine regimen, thus providing a greater analytic power.

**Implications of all the available evidence**

The findings of the HIV-CORE 006 trial concerning both the primary and secondary outcomes lend credence to the integration of the HIVconsvX T-cell candidate vaccines into forthcoming HIV-1 cure strategies.

Guided by most licensed vaccines that work through the induction of antibodies, HIV-1 vaccine efforts have aimed to induce broadly neutralising antibodies. However, given the selective pressure on HIV-1 imposed by CD8^+^ T cells, a vaccine will probably require a concerted effort of both antibody and cellular immune responses primed within the context of the appropriate innate environment. An effective vaccine must, therefore, evoke multiple protective mechanisms that function together.

Our team aims to develop a vaccine strategy for inducing killer T cells that preferentially recognise vulnerable parts of HIV-1 and contribute to protection. This strategy implies a more complex and sophisticated approach, involving the appropriate set of immunogens presented to the immune system by the right types of vaccine modalities (vectors assembled into a regimen), and delivered through the correct routes of administration. We recognise that not all T cells are equally protective and hypothesise that targeting functionally conserved regions of HIV-1 proteins—those that tend to be subdominant in natural infection and, therefore, underutilised—is one of the key prerequisites for cellular immunity.^5^ We have made iterative improvements to vaccine immunogens and vector regimens informed by human data. Following earlier HIVA^6^ and HIVconsv^7^ vaccines, our current iteration, HIVconsvX, uses bivalent mosaic immunogens of six cross-clade conserved Gag and Pol regions, which are delivered through a sequential administration of replication-deficient vectors ChAdOx1 derived from a simian (chimpanzee) adenovirus and poxvirus modified vaccinia virus Ankara (MVA).^8^ The advantage of this design is that it holds the potential to be universally deployed in any geographical region, irrespective of the local circulating clades. The first-in-human trial (named HIV-CORE 005.2) in healthy individuals living without HIV-1 in Oxford, UK, showed that HIVconsvX vaccines were well tolerated and induced robust, broad, and polyfunctional T-cell responses.^9^ In this current study, HIV-CORE 006, we aimed to assess the safety and immunogenicity of the same HIVconsvX vaccines in healthy individuals living without HIV-1 at four clinical research centres (CRCs) in sub-Saharan Africa in areas with circulating HIV-1 clades A, D, and C.

## Methods

### Study design

HIV-CORE 006 was a double-blind, randomised, placebo-controlled, phase 1 clinical trial at four CRCs in Uganda, Kenya (two CRCs), and Zambia. The four CRCs were: the Medical Research Council/Uganda Virus Research Institute and London School of Hygiene and Tropical Medicine Uganda Research Unit (MUL; Masaka, Uganda); the KEMRI-Wellcome Trust Research Programme (KWTRP; Kilifi, Kenya); the KAVI-Institute for Clinical Research, University of Nairobi (KAVI-ICR; Nairobi, Kenya); and the Center for Family Health Research Zambia (CFHRZ; Lusaka, Zambia).

Ethical approval for HIV-CORE 006 was granted for MUL by the Uganda Virus Research Institute Research and Ethics Committee (reference number GC/127/20/06/761), the Uganda National Council for Science and Technology (reference number HS844ES), and the National Drug Authority (reference number CTC 0171/2021); for the KWTRP by the Clinical Science Committee (protocol number CSC 180) KEMRI Scientific and Ethics Review Unit (protocol number KEMRI/SERU/CGMR-C/180-2020/4025), National Commission for Science, Technology, and Innovation (reference number 286477; licence number NACOSTI/P/20/6699), and the Pharmacy and Poisons Board (PPB; reference number PPB/ECCT/20/10/01/2021); for KAVI-ICR by the Kenyatta National Hospital Ethics Research Committee (reference number P863/10/2019) and PPB (reference number PPB/ECCT/20/06/09/2020); and for the CFHRZ by the University of Zambia Biomedical Research Ethics Committee (UNZABREC; reference number 495-2019), the Zambia Medicines Regulatory Authority (clinical trial number CT-098), the National Biosafety Authority (reference number NBA/101/16/1), the National Health Research Authority, and the Oxford Tropical Research Ethics Committee (OxTREC; reference number 56-19). The study was conducted according to the principles of the 2008 Declaration of Helsinki and complied with the International Conference on Harmonization Good Clinical Practice guidelines and Good Participatory Practices.^10^ The clinical trial protocol (CTP) is provided in the appendix (p 26).

### Participants

Healthy adult male participants and female participants who were not pregnant were recruited if they were living without HIV-1 or HIV-2, had a low likelihood of HIV-1 acquisition, aged 18–50 years, fully comprehended the purpose and details of this study as provided in the participant information sheet, and passed the assessment of understanding before providing written informed consent. As per the CTP (appendix p 26)**,** eligibility depended on the results of laboratory tests, review of medical history, physical examination results, and answers to questions about behaviours that could increase the chance of acquiring HIV-1. For COVID-19 vaccinations, participants must have been vaccinated with a non-adenoviral vaccine or had their COVID-19 vaccine offered at least 3 months after ChAdOx1.tHIVconsv1 (C1) to avoid interfering with the SARS-CoV-2 vaccine response. There was no selection of participants based on pre-existing neutralising antibodies to human adenovirus serotype 5 or MVA. All participants were tissue-typed for HLA class I and class II alleles using sequence-specific primer PCR.

### Randomisation and masking

Participants were randomly assigned in a 9:2 ratio to receive either the vaccine or the placebo at enrolment according to the randomisation schedule prepared by the statisticians at the Data Coordinating Centre (DCC; Oxus Technologies, Oxford, UK) and described in the CTP section 8.6 (appendix p 57). Upon entry into the data system, participants were automatically given a unique allocation number. This number corresponded to a treatment group in a randomisation list, which was provided solely to the unblinded site pharmacists by the DCC, ensuring that researchers and the enrolling staff were unaware of the treatment group assignment during enrolment. The pharmacists drew the vaccine or placebo into a syringe, which was then covered with tape to conceal the drawn solution from the administering nurse’s view. Once the database was locked, participants were informed of their assignment (vaccine or placebo) upon study completion.

### Procedures

**Figure 1:**
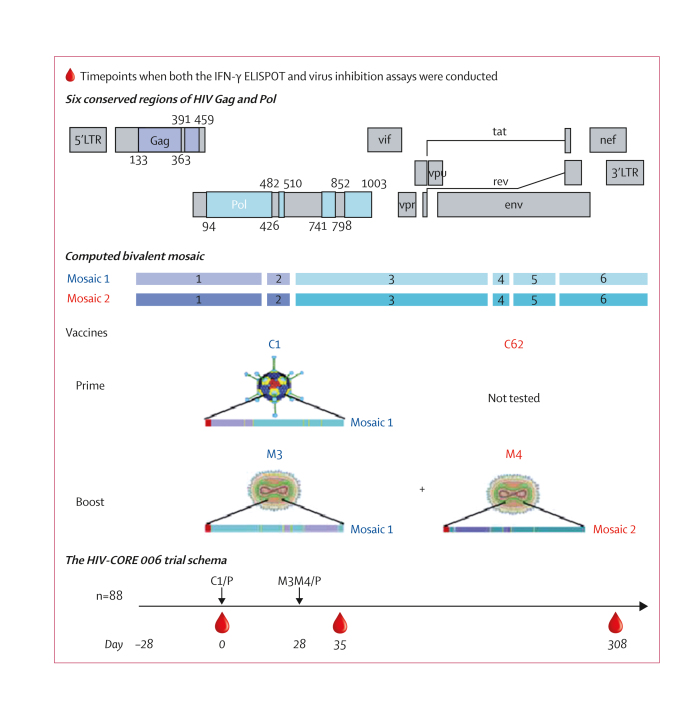
Vaccine design and trial schema Alignments of curated global HIV-1 protein sequences available from the Los Alamos National Laboratory HIV Sequence Database as of September, 2013, were used to compute two mutually complementary mosaic sequences of the complete Gag and Pol proteins and to identify highly conserved regions within them. These regions were arranged into six distint proteins collectively referred to as HIVconsvX and the individual genes were inserted into simian adenovirus or poxvirus vectors to generate vaccines C1, C62 (not tested in HIV-CORE 006 due to delayed manufacturing), M3, and M4. In the HIV-CORE 006 trial, people living without HIV-1 in sub-Saharan Africa received either the vaccine regimen (C1-M3M4; n=72) or placebo (n=16) and were followed up for 40 weeks following the last vaccination. C1=ChAdOx1.tHIVconsv1. C1/P=C1 or placebo. C62=ChAdOx1.HIVconsv62. M3=MVA.tHIVconsv3. M4=MVA.tHIVconsv4. M3M4/P=M3M4 or placebo.

The HIVconsvX T-cell vaccines were designed for global HIV-1 coverage (figure 1). The immunogens were computed as two mutually complementary mosaics of six highly conserved Gag and Pol regions resulting in an 80% match of the vaccine’s potential T-cell epitopes^11^ to those of globally circulating HIV-1 variants of group M.^8^ The mosaic design further enhances the match of the vaccine to the circulating HIV-1 variants, because even functionally conserved regions exhibit a degree of variation at the epitope level. These regions were rearranged into six unique configurations to prevent the generation of strong irrelevant junctional responses observed with the previous immunogen version HIVconsv.^12^ Candidate vaccine components C1 (mosaic 1 with regions ordered 1-2-3-4-5-6), MVA.tHIVconsv3 (M3; mosaic 1 with regions ordered 3-6-2-5-1-4), and MVA.tHIVconsv4 (M4: mosaic 2 with regions ordered 4-1-5-2-6-3) were administered in heterologous prime-boost regimen C1-M3M4 with 28 days between the prime and boost. The follow-up period was 40 weeks (figure 1). The ChAdOx1.HIVconsv62 vaccine was not used in this trial; however, as long as either the prime or boost uses the mosaic pair, recognition of epitope variants is improved compared with a fully monovalent prime-boost.^13^ No inadvertent mRNA splicing out of or into the tHIVconsv1 or HIVconsv62 transgenes was detected, further increasing confidence in the safety of these ChAdOx1-vectored vaccines.^14^

Vaccines were thawed no more than 30 min before injection, kept on ice, and administered into the deltoid muscles on both left and right arms by an intramuscular needle injection. Vaccine recipients were administered with C1 (5 × 10^10^ viral particles divided equally into each arm) at enrolment on day 0 followed by M3 (1 × 10^8^ plaque-forming units [PFUs] into the left arm) and M4 (0·9 × 10^8^ PFUs into the right arm) on day 28. Sterile 0·9% saline was injected as a placebo on days 0 and 28 (figure 1).

### Outcomes

The first primary outcome was the vaccines’ safety assessment. Study visits for safety evaluations occurred 1, 7, 14, and 28 days after each vaccination, with additional visits on days 84, 112, 210, 308, and 336. Safety data included specified solicited symptoms (appendix p 61) collected by diary cards for 7 days after each vaccination, unsolicited adverse events collected for up to 28 days after each vaccination; and serious adverse events collected until the end of the study up to 40 weeks after vaccination. Blood samples for the evaluation of biochemical and haematological parameters were taken at prespecified study visits on days –28, 0, 7, 28, 35, 56, 210 and 336 (appendix p 90). The severity of clinical and laboratory adverse events was assessed according to the scales in the Division of AIDS Table for Grading the Severity of Adult and Paediatric Adverse Events (corrected version 2.1, July, 2017; appendix p 93).

Other outcomes characterised the vaccine-elicited T cells. Thus, the second primary outcome evaluated the C1-M3M4 regimen’s induction of HIVconsvX-specific T-cell responses by assessing the proportion of vaccine recipients who responded to the vaccination. The secondary outcome assessed the magnitude, breadth, and duration of the vaccine-elicited responses measured using HIVconsvX peptide pools in an IFN-γ ELISPOT assay. Exploratory analyses evaluated the HIVconsvX-specific T-cell ability to inhibit HIV-1 replication in virus inhibition assays (VIAs). These analyses used infectious molecular clones of engineered HIV-1 that maintained physiological levels and functions of the Nef protein, a negative regulatory factor that helps HIV-1 to evade immune responses, and produced the *Renilla reniformis* luciferase reporter enzyme used to measure HIV-1 growth. As post-hoc analyses, we performed comparisons of three age subgroups (age 19–25 years, age 26–35 years, and age 36–50 years) and of genders (male or female at birth). For flow cytometry analyses, because it would have been highly labour-intensive to analyse all 72 vaccine recipients, we selected five strong responders to the vaccine regimen in each CRC. Materials and methods for the ELISPOT, flow cytometry, and VIAs are detailed in the appendix (p 2).

### Statistical analysis

Analyses of variance were conducted using GraphPad Prism (version 10.2.0). For safety, all participants who received at least one vaccine or placebo dose were included in the analyses. For immunogenicity, only those participants who received all doses of vaccine or placebo as per protocol were included in the primary and secondary analyses. The primary and secondary immunological results were those obtained using the ELISPOT assay. These results were assumed to be non-Gaussian in distribution, so non-parametric tests were used throughout and medians (IQR range) are presented, unless stated otherwise. The tests used for data analyses are indicated in the figure legends. Two-tailed p values were applied, and a p value of less than 0·05 was regarded as statistically significant. Every effort was made to collect all data in accordance with the protocol. Given that missing data were minimal, no imputation was performed for missing values.

### Role of the funding source

The funder of the study had no role in study design, data collection, data analysis, data interpretation, or writing of the report.

## Results

**Figure 2:**
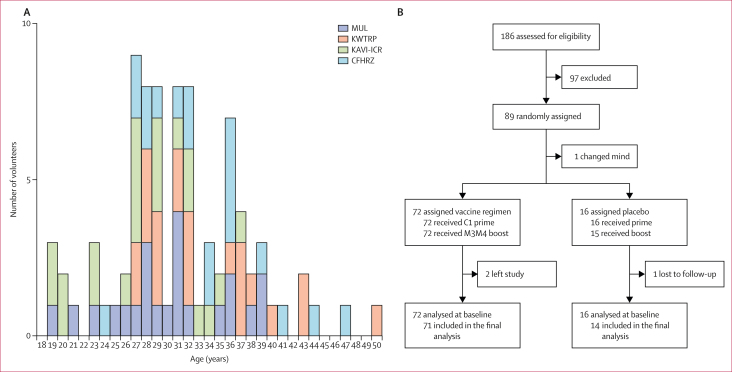
Study population and the CONSORT diagram (A) Age distribution of the study individuals. (B) CONSORT diagram. C1=ChAdOx1.tHIVconsv1. CFHRZ=the Center for Family Health Research Zambia. KAVI-ICR=the KAVI-Institute for Clinical Research. KWTRP=the KEMRI-Wellcome Trust Research Programme. M3M4=MVA.tHIVconsv3 and MVA.tHIVconsv4. MUL=the Medical Research Council/Uganda Virus Research Institute and London School of Hygiene and Tropical Medicine Uganda Research Unit.

The HIV-CORE 006 trial was conducted at four CRCs: at MUL in Masaka, Uganda; at KWTRP in Kilifi and at KAVI-ICR in Nairobi, Kenya; and at CFHRZ in Lusaka, Zambia. Together at the four trial CRCs, 186 healthy adults living without HIV-1 were screened for eligibility, 89 were randomly assigned, and 88 were injected with either the vaccine (n=72) or placebo (n=16; appendix p 19). Median participant age was 30 years (IQR 27–36). Of the 88 participants, 57 (65%) were male and 31 (35%) were female (table 1; appendix p 19). The full list of reasons for screening failure is shown in the appendix (p 17). All enrolled participants completed the protocol except for two: one participant (in the vaccine group) withdrew before the first dosing for reasons unrelated to the trial and was replaced and one participant (in the placebo group) was lost to follow-up. The participant recruitment is captured in the CONSORT diagram (figure 2B).

**Table 1:** Baseline characteristics of participants

	KAVI-ICR (n=22)	MUL (n=22)	KWTRP(n=22)	CFHRZ (n=22)	Overall (n=88)
Age, years	27 (23–32)	31 (27–35)	32 (29–37)	34 (28–36)	31 (28–36)
Height, cm	154–186	148–190	163–182	149–185	148–190
Body mass, kg	46–106	46–91	48–84	44–98	44–106
Sex at birth					
Male	8 (36%)	17 (77%)	21 (91%)	11 (50%)	57 (64%)
Female	14 (64%)	5 (23%)	2 (9%)	11 (50%)	32 (36%)

Data are median (IQR), range, or n (%). CFHRZ=the Center for Family Health Research Zambia. KAVI-ICR=the KAVI-Institute for Clinical Research, University of Nairobi. KWTRP=the KEMRI-Wellcome Trust Research Programme. MUL=the Medical Research Council/Uganda Virus Research Institute and London School of Hygiene and Tropical Medicine Uganda Research Unit.

All three vaccine components (C1, M3, and M4) were well tolerated in the trial population. No serious adverse events were recorded, and the most frequently reported adverse events included pain at the injection site, headache, general malaise, fatigue, and myalgia (table 2). Overall, a total of 302 solicited adverse events were recorded following the C1 vaccination, of which 160 (53%) were classified as local reactogenicity and 142 (47%) as systemic. Among all the solicited adverse events after C1 vaccination, 249 (82%) were grade 1, 53 (18%) were grade 2, and none were grade 3. Following the administration of M3 and M4, 28 days after C1, there was no difference in local reactogenicity between the two vaccines, with a total of 517 adverse events recorded, of which 234 (45%) were local and 283 (55%) systemic. Of these 517 adverse events, 275 (53%) were grade 1, 234 (45%) were grade 2, and eight (2%) were grade 3. All adverse events resolved spontaneously within 72 h. Among the placebo recipients, 31 local and 45 systemic adverse events were recorded (table 2). Overall, the C1-M3M4 regimen was well tolerated.

**Table 2:** Maximum severity of solicited adverse events

	C1 (n=72)			M3M4 (n=72)			Placebo (n=16)			Placebo (n=16)		
	Grade 1	Grade 2	Grade 3	Grade 1	Grade 2	Grade 3	Grade 1	Grade 2	Grade 3	Grade 1	Grade 2	Grade 3
**Local solicited reactogenicity**												
Pain	63	10	0	52	55	0	3	0	0	3	0	0
Tenderness	65	15	0	60	55	0	10	0	0	10	0	0
Erythema	4	0	0	2	1	0	0	0	0	0	0	0
Swelling	2	1	0	8	1	0	1	0	0	1	0	0
**Systemic solicited adverse events**												
Arthralgia	2	1	0	9	3	1	1	0	0	1	0	0
Myalgia	14	3	0	22	19	0	1	0	0	3	0	0
Fatigue	19	3	0	19	19	2	6	1	0	5	0	0
Sweating	7	1	0	14	8	0	0	0	0	2	1	0
Malaise	1	1	0	4	2	0	1	0	0	0	1	0
Vomiting	21	4	0	24	19	2	1	0	0	3	1	0
Nausea	12	1	0	14	11	0	2	1	0	1	1	0
Headache	17	6	0	15	16	2	2	0	0	1	1	0
Chills	11	4	0	15	14	1	1	0	0	1	1	0
Fever	11	3	0	17	11	0	1	1	0	2	1	0

88 volunteers received each of the vaccines once. C1=ChAdOx1.tHIVconsv1. M3M4=MVA.tHIVconsv3 and MVA.tHIVconsv4.

Reported unsolicited adverse events deemed related to vaccinations included upper respiratory tract infections, influenza-like illness, and musculoskeletal abnormalities. There were 25 and four unsolicited adverse events in the vaccine and placebo groups, respectively. These unsolicited adverse events were all of grade 2 in severity, except for one that was of grade 3 in severity. All unsolicited adverse events were short-lived, resolving within 48 h from onset. One grade 3 reactogenicity event was detected in one participant in the placebo group (appendix p 20). The per-protocol grade 3 systemic laboratory abnormality was reviewed by the independent Data Monitoring and Ethics Committee (DMEC), who decided on no specific follow-up action (appendix p 21).

**Figure 3:**
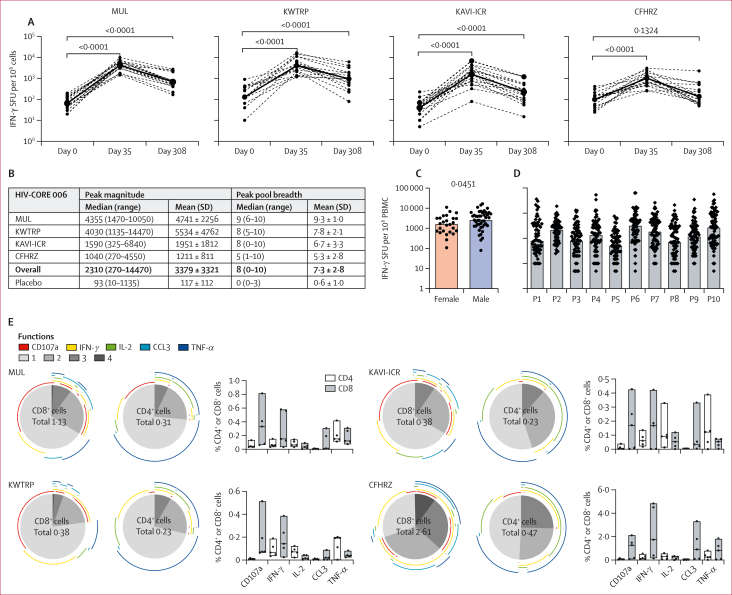

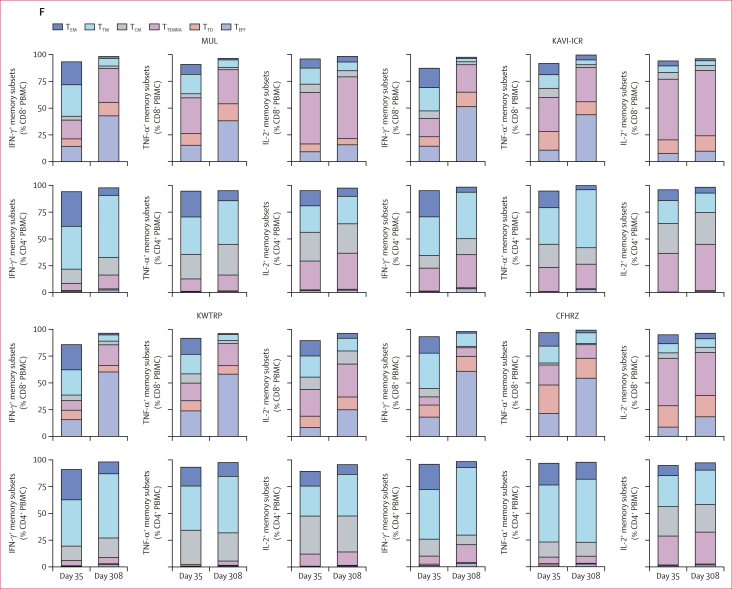
**Strong and broad T-cell responses induced by the HIVconsvX vaccines in individuals without HIV-1** (A) The HIVconsvX vaccine-specific T-cell responses were enumerated in an IFN-γ ELISPOT assay using HIVconsvX peptide pools one to ten on day 0, day 35 (peak), and day 308. The p values reported were calculated using the Wilcoxon matched-pairs signed rank test. (B) A summary of the total peak magnitudes and the number of peptide pools recognised out of 10 at the peak of T-cell responses on day 35 is provided. Medians with individual specific T-cell frequencies on day 35 in female and male vaccine recipients (Mann–Whitney test; C) and for individual HIVconsvX pools (D) are presented. (E) Functionality of HIVconsvX-specific CD8^+^ and CD4^+^ T cells. Five strong responders from each CRC were selected for the polychromatic flow cytometry analysis following stimulation of day 35 peripheral blood mononuclear cells with personalised peptide pools. Average frequencies are depicted as central pie charts illustrating the number of functions of individual cells depicted in shades of grey with functions colour-coded around the periphery (left). Total frequencies for each function are displayed as median (range) boxes indicating individual values (right). For gating, refer to the appendix (p 12). (F) T-cell memory structure. The memory structure of vaccine-elicited T cells was analysed by flow cytometry for their subpopulations defined as T_EM_ (effector memory; CD45RA^Neg^CCR7^Lo^CD27^Neg^), T_TM_ (transitional memory; CD45RA^Neg^CCR7^Lo^CD27^Pos^), T_CM_ (central memory; CD45RA^Neg^CCR7^Hi^CD27^Pos^), T_TEMRA_ (terminal effector memory re-expressing CD45RA^Pos^CCR7^Hi^CD27^Pos^), T_TD_ (terminally differentiated; CD45RA^Pos^CCR7^Lo^CD27^Neg^), CD45RA^Pos^CCR7^Hi^CD27^Pos^), and T_EFF_ (effector; CD45RA^Pos^CCR7^Lo^CD27^Pos^). See appendix for gating (p 12). Graphs show averaged percentages over five strong responders from each CRC. CFHRZ=the Center for Family Health Research Zambia. CRC=clinical research centre. KAVI-ICR=the KAVI-Institute for Clinical Research. KWTRP=the KEMRI-Wellcome Trust Research Programme. MUL=the Medical Research Council/Uganda Virus Research Institute and London School of Hygiene and Tropical Medicine Uganda Research Unit. P=peptide pool. PMBC=peripheral blood mononuclear cell. SFU=spot-forming unit.

The C1-M3M4 regimen was highly immunogenic and significantly (p<0·0001) increased HIV-1-specific responses in 70 (99%) of 71 participants who received all vaccine doses as measured by IFN-γ ELISPOT assay. Across the four CRCs, HIVconsvX-specific T-cell frequencies peaked at a median of 2310 (IQR 1080–4480) spot-forming units (SFUs) per 10^6^ peripheral blood mononuclear cells (PBMCs) and at a mean of 3379 (SD 3321) SFUs per 10^6^ PBMCs (figure 3B). Peak T-cell responses decreased to a median of 500 (IQR 150–950) SFUs per 10^6^ PBMCs over the 40 weeks of follow-up (figure 3A; appendix p 4). A one-way ANOVA Kruskall–Wallis test showed significant variations among the site peaks of total T-cell response magnitudes (p<0·0001). In contrast, responses in all combined recipients of the placebo regimen from the four CRCs peaked at a median of 93 (IQR 43–160) SFUs per 10^6^ PBMCs (figure 3B; appendix p 4). In the vaccine groups, male recipients had significantly higher responses than female recipients (p=0·0451; figure 3C). Vaccine-induced T cells recognised a median of eight (IQR five to ten) of ten peptide pools spanning the HIVconsvX immunogen (appendix p 4) with responses evenly distributed across individual pools (figure 3D). We examined the breadth of epitope recognition within the HIVconsvX immunogen in greater detail (appendix p 8). 10-day pool-expanded short culture T-cell lines (SCTLs) from 53 randomly selected participants, assessed by a laboratory that was masked to group assignment, recognised a mean of 17·7 (SD 14·4) 15-mer peptide pairs per individual stimulating at least 750 IFN-γ SFU per 10^6^ SCTL cells, some of which were overlapping. The identified stimulatory peptide pairs were used to assemble personalised peptide pools for flow cytometry analyses.

As per exploratory outcomes, we assessed the polyfunctionality of the C1-M3M4-elicited CD8^+^ and CD4^+^ T cells characterised for five strong responders from each CRC using personalised peptide pools identified in STCL mapping (appendix p 8). The studied functions included the production of IFN-γ, TNF-α, IL-2, and MIP-1α (also known as CCL3), along with degranulation, which is measured by the surface expression of CD107a and considered equivalent to cytotoxicity (appendix p 2). The vaccine regimen induced polyfunctional T-cell populations with the proportions of individual CD8^+^ T cells displaying one, two, three, and four functions at medians of 66% (IQR 48–72), 24% (20–29), 9% (7–18), and 1% (0·5–5·3) of total CD8^+^ cells, respectively. By contrast, CD4^+^ T cells exhibited such polyfunctionalities at medians of 63% (52–73), 23% (20–30), 10% (1–18) and 0·2% (0·1–0·4) of individual CD4^+^ T cells, respectively. Although these overall summaries are not depicted, for each CRC we show pie charts of the functional distribution (figure 3E, left side) and graphs illustrating the absolute proportions of each function as a percentage of total CD8^+^ and CD4^+^ T cells (figure 3E, right side; see appendix p 12 for the gating strategy). Therefore, the HIVconsvX vaccine-elicited CD8^+^ and CD4^+^ T cells were polyfunctional and capable of proliferation, followed by functional expression upon antigenic stimulation.

As part of an exploratory analysis, five strong responders at each CRC were evaluated for the peak response on day 35 focusing on the composition of the T-cell memory and the evolution of its subpopulations over the study period, which extended to day 308. A clear distinction between the architectures of CD8^+^ and CD4^+^ T-cell memory was readily identified (figure 3F; appendix p 12). Over the 40 weeks of follow-up, CD8^+^ T cells notably expanded the T effector cell (T_EFF_) population while simultaneously decreasing the populations of the T effector memory cell (T_EM_) and transitional memory T cell (T_TM_) populations. Among CD4^+^ T cells, the most significantly expanded and reduced subpopulations were, respectively, the T_TM_ and T_EM_ populations. These features were replicated well in the four CRC populations.

**Figure 4:**
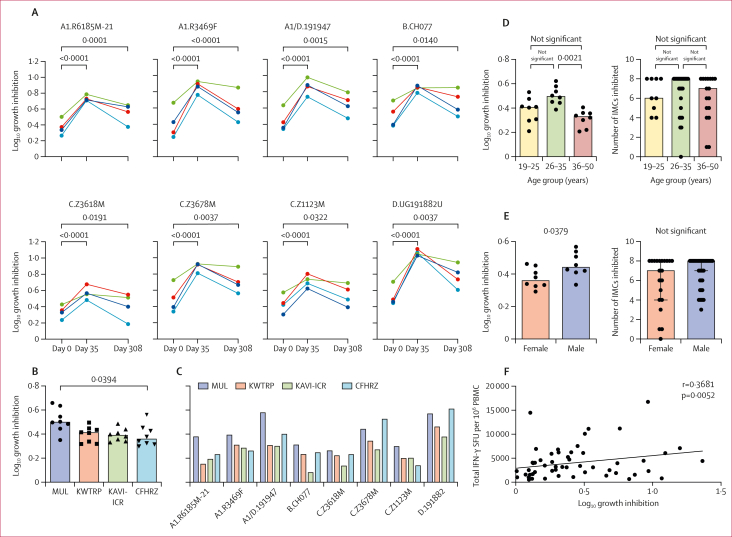
Cross-clade inhibition of HIV-1 growth by vaccine-elicited CD8 T cells All data concerning the inhibition of eight HIV-1 IMCs growth inhibition by vaccine-elicited CD8^+^ effectors in autologous CD4^+^ cells are provided in the appendix (p 14). (A) The graphs show the median vaccine-elicited inhibition by CD8^+^ T cells over eight HIV-1 IMCs for each CRC. The overall inhibition across all CRCs and all viruses was assessed and compared at pre-vaccination (day 0), peak response (day 35), and study conclusion (day 308) using the Friedman test with Dunn’s multiple comparison-adjusted values. p values are indicated above the graphs. (B) A comparison of four CRCs regarding the overall inhibition of eight IMCs on day 35 is presented, showing the medians for each IMC inhibition. The Kruskal–Wallis multiple comparison test with Dunn’s correction found no statistically separable results except for a significant difference between MUL and CFHRZ. (C) The same data from day 35 are presented as medians for each IMC and CRC. Subsequently, vaccine recipients were categorised into three age groups (D) and by gender (E) to compare overall IMC inhibition (left) and the number of inhibited IMCs by ≥0·1 log_10_ (right). Medians are shown alongside individual values with significant p values shown above, obtained through the Mann–Whitney test and the Kruskal–Wallis test with multiple comparison corrections, respectively. (F) The graph illustrates the correlation between peak ELISPOT frequencies and the growth inhibition caused by effector CD8^+^ cells elicited by the C1-M3M4 vaccine regimen applying simple linear regression. CFHRZ=the Center for Family Health Research Zambia. CRC=clinical research centre. IMC=infectious molecular clones. KAVI-ICR=the KAVI-Institute for Clinical Research. KWTRP=the KEMRI-Wellcome Trust Research Programme. MUL=the Medical Research Council/Uganda Virus Research Institute and London School of Hygiene and Tropical Medicine Uganda Research Unit. PBMC=peripheral blood mononuclear cell. SFU=spot-forming unit.

One of the secondary objectives was to assess the breadth of in-vitro inhibition of HIV-1 replication by vaccine-induced CD8^+^ T cells. In this study, VIAs used eight infectious molecular clones (IMCs) of HIV-1, which originated from several countries and represented the global clades A, B, C and D (appendix p 21).^15^^,^^16^ Initially, we detected participant-specific differences in the ability of CD4^+^ T cells to support HIV-1 replication (appendix p 13); this donor-dependent variation in HIV-1 replication concurs with previous reports.^17^ However, HIV-1 replication was sufficient to allow for the assessment of CD8^+^ T-cell mediated inhibition of all but one IMC in one participant with insufficient growth (appendix p 22) out of the 60 randomly selected participants tested among all four CRCs. The VIA laboratory was masked to the individuals’ allocation to vaccine or placebo, and three placebo recipients were included. Non-specifically expanded CD8^+^ T cells mediated broad cross-clade HIV-1 inhibition in most individuals following vaccination, with minimal inhibition observed in participants who received the placebo (figure 4A; appendix p 22). Overall, the HIVconsvX vaccination induced CD8^+^ T cells, that exhibited increased inhibition by at least 0·1 log_10_ of the mean of 6·4 (SD 0·7) IMCs at the peak response on day 35 (p<0·0001), and of the mean of 4·8 (SD 0·2) IMCs at the study conclusion compared with the pre-vaccination cultures. The differences in overall inhibition levels of the eight tested IMCs were significant between the MUL and CFHRZ CRCs (p=0·039; figure 4B), whereas differences in the inhibition of individual IMCs among CRCs were not significant (figure 4C). It is noteworthy that Kenya and Uganda predominantly have circulating clades A and D, whereas Zambia has C. In post-hoc comparisons, only the age group of 26–35 years inhibited HIV-1 significantly more efficiently than the 36–50-year age group (p=0·0021), while other age-based comparisons among vaccine recipients remained statistically inseparable (figure 4D). Additionally, we observed more efficient growth inhibition in male vaccine recipients than in their female counterparts (p=0·0379; figure 4E). Furthermore, a positive correlation was observed between the total IFN-γ ELISPOT frequencies and virus inhibition (p=0·0052; figure 4F). Overall, HIVconsvX-specific T cells inhibited HIV-1 representing four major global clades.

The HLA class I and II alleles of all 84 recruited participants were established genetically, and the frequencies of class I molecules along with the HLA-A and B supertypes were calculated. A total of 95 unique protein sequences were detected, which included 30 (32%) from HLA-A supertypes, 36 (38%) from HLA-B supertypes, and 29 (31%) from HLA-C supertypes. The most frequent alleles for the three class I loci were HLA-A^∗^02:01 and A∗68:02, HLA-B∗35:01 and B∗42:01, and HLA-C∗04:01 and C∗06:02. The HLA class I prevalence differed from that of the White British population and aligned well with previous data from these geographical regions of Africa.^18^ There was no significantly outstanding HLA allele associated with high ELISPOT frequencies or growth inhibition (appendix p 16).

## Discussion

Selective targeting of multiple vulnerable sites on HIV-1 constitutes a rational T-cell vaccine strategy supported by emerging results from several human studies.^2^^,^^3^^,^^8^^,^^19^, ^20^, ^21^ Here, we showed the safety and immunogenicity of experimental HIVconsvX vaccines, which were designed as a bivalent mosaic comprising six functionally conserved Gag and Pol sub-protein regions^8^ in adults living without HIV-1 within affected communities in Kenya, Uganda, and Zambia. The clinical protocol was developed by a multidisciplinary team from four sub-Saharan CRCs with contributions from the priority communities^10^ and assistance from Oxford University, Oxford, UK, and the International AIDS Vaccine Initiative (IAVI, New York, NY, USA). The vaccines exhibited an acceptable safety profile and elicited strong and broad T-cell responses that were polyfunctional, capable of exerting at least one function after proliferation, and inhibited representative HIV-1 isolates from major global clades.

The vaccine-elicited T-cell frequencies ranged between 1040 and 4355 IFN-γ SFUs per 10^6^ PBMCs (figure 3B). The HIV-CORE 006 median frequency represented 53% of that reported in the first-in-human trial in the UK testing the same vaccine regimen.^9^ Lower overall responses in Africa could reflect differences in the participants’ HLA profiles, various genetic factors, nutritional status, and exposure to environmental pathogens (eg, adenovirus serostatus). Nevertheless, the HIVconsvX results from both the UK and sub-Saharan Africa compare favourably with previous vaccines tested for the prevention of HIV-1 acquisition.^22^, ^23^, ^24^, ^25^, ^26^, ^27^ Also notable are the lower responses in female vaccine recipients than in male vaccine recipients (figure 3C), contrasting with the five times higher frequencies of female individuals living with HIV-1 displaying elite HIV-1 control in the absence of antiretroviral treatment compared with male individuals living with HIV-1.^28^ Recipients of the vaccine in HIV-CORE 006 recognised a median of 8 (5–10) out of 10 peptide pools in an IFN-γ ELISPOT assay (figure 3B), which typically detects approximately equal numbers of CD8^+^ and CD4^+^ T-cell specificities.^12^^,^^24^^,^^25^^,^^29^^,^^30^ This immunogenicity is similar to that of the prototype HIVconsv vaccine and other heterologous prime-boost regimens using adenoviral and poxviral vectors,^2^^,^^12^^,^^30^, ^31^, ^32^ as repeated administrations of the same vector tend to be less immunogenic, particularly for T cells.^29^^,^^33^^,^^34^

VIA collectively assesses the protective antiviral functions of CD8^+^ T cells leading to the inhibition of HIV-1 growth in tissue culture and is carried out with various adaptions.^21^^,^^35^, ^36^, ^37^, ^38^, ^39^, ^40^ VIA can compare responses induced by different experimental vaccines to prioritise their further clinical development and estimate the breadth of inhibition across multiple HIV-1 isolates. The broad cross-clade reach observed in the HIV-CORE 006 trial (figure 4; appendix p 22) supported the hypothesis of using conserved protein regions and concurred with the strong abilities of the HIVconsvX protective epitopes in Japanese individuals living with HIV-1 who had no previous or ongoing treatment to suppress the virus.^20^^,^^21^ A prospective study identified an inverse correlation between ex-vivo virus inhibition and the rate of CD4^+^ T-cell decline.^41^ In the AELIX 002 trial**,** vaccine recipients showed capacity for increased virus inhibition compared with placebo recipients, but this was not associated with any outcomes from the analytical treatment interruption (ATI).^2^ Herbert and Goulder proposed that T cells play a role in reducing plasma viral load (pVL) at set points, but their influence is less pronounced in controlling post-treatment viral recrudescence, a process potentially driven more by natural killer cells.^42^

As with our previous vaccine versions, the HIVconsvX vaccines were administered into the deltoid muscles of both arms.^12^^,^^30^ This two-site administration ensured that both arms received the same number of doses and allowed for a separate assessment of the local reactogenicities of M3 and M4. Several experiments in pre-clinical models suggested that parallel injections into multiple anatomical sites could be beneficial for the vigour and breadth of the vaccine-elicited responses.^43^, ^44^, ^45^ In contrast, other studies reported that mixed variants and mixed T-cell or B-cell vaccines induced more potent overall responses than when the components were given at different times or into anatomically separate locations.^46^^,^^47^ The benefits of multisite vaccination were observed in a human study.^48^

Replication-deficient vector ChAdOx1 derived from the chimpanzee adenovirus is used for priming. During the COVID-19 pandemic response, adenoviral vaccines were linked to extremely rare blood clots occurring in approximately four individuals in a million who received the vaccine.^49^ Although it is crucial to understand the risks of thrombotic and cardiovascular complications arising from vaccination and to always monitor the signs of these adverse events of special interest, 3 billion doses of the ChAdOx1 nCoV-19 vaccine were administered to individuals in over 170 countries, saving an estimated 6 million lives. Consequently, we do not foresee that the use of the ChAdOx1 vector will hinder the development of the C1 and C62 vaccine programme.

In conclusion, protective T cells have the potential to profoundly contribute to HIV-1 prevention and cure. We have now been pioneering conserved region T-cell vaccines for nearly two decades, systematically testing and refining their design while ensuring their relevance to the most affected geographical regions. The strengths of the present study include the well characterised vaccine vectors and the recruitment of participants from the areas most affected by the HIV-1 epidemic. The main weakness is the focus on responses in the PBMC, whereas the control of HIV-1 infection will be primarily determined in the lymphoid organs and tissues such as the gut.^50^ We foresee the future development and use of the HIVconsvX vaccines as a potentially key component of a combined package of tools for cure and prevention. The vaccine's effect on pVL as part of an ATI would provide robust evidence of in-vivo viral control and allow breaks from antiretroviral treatment for people living with HIV-1. Even a transient effect on pVL would yield valuable data regarding viral escape mechanisms. In people living without HIV-1, an effective vaccine could serve as a backup for pre-exposure prophylaxis. Data on the safety and immunogenicity of the HIVconsvX vaccines from trials involving people living with HIV-1 will be available in the near future. Results from trials evaluating the contribution of T-cell vaccines to clinical efficacy during ATI will begin to emerge in the coming years.

## Data sharing

De-identified participant data will be made available upon requests directed to TH. The sponsor, chief investigator, and collaborators will review and approve proposals based on scientific merit. After a proposal is approved, data can be shared through a secure online platform after signing a data access agreement.

## Declaration of interests

TH is a co-inventor of the HIVconsvX immunogens protected under EP14846993.5 and PCT/US14/58422 (WO2015048785). All other authors declare no competing interests.
