# Supplementary material for: Safety and broad immunogenicity of HIVconsvX conserved mosaic candidate T-cell vaccines vectored by ChAdOx1 and MVA in HIV-CORE 006: a double-blind, randomised, placebo-controlled phase 1 trial in healthy adults living without HIV-1 in eastern and southern Africa
Source: Lancet Microbe. 2025 Jun;6(6):None. doi: 10.1016/j.lanmic.2024.101041 (PMC12134052; doi:10.1016/j.lanmic.2024.101041)
Supplement: Supplementary appendix [file mmc1.pdf]

THE LANCET
Microbe

Supplementary appendix

This appendix formed part of the original submission and has been peer reviewed.
We post it as supplied by the authors.

Supplement to: Chanda C, Kibengo F, Mutua M, et al. Safety and broad immunogenicity of HIVconsvX conserved mosaic candidate T-cell vaccines vectored by ChAdOx1 and MVA in HIV-CORE 006: a double-blind, randomised, placebo-controlled phase 1 trial in healthy adults living without HIV-1 in eastern and southern Africa. *Lancet Microbe* 2025. https://doi.org/10.1016/j.lanmic.2024.101041

Supplementary materials

Materials and methods 2

Figure S1: **Fresh IFN-γ ELISPOT responses shown for individual study subjects** 4

Figure S2: **Volunteers' HLA class I and peptide pair mapping by STCL** 8

Figure S3: **Gating strategy for *ex vivo* functional analysis of vaccine-elicited T-cells** 12

Figure S4: **Gating strategy for *ex vivo* memory structure of vaccine-elicited T-cells** 12

Figure S5: **Growth if IMCs of HIV-1 in volunteers' $CD4^+$ cells** 13

Figure S6: **Alignment of HIVconsvX mosaics 1 and 2 with the VIA IMCs** 14

Figure S7: **HLA class I allele frequencies and corresponding ELISPOT frequencies and VIA** 16

Table S1: **Reasons for screen failure (by CRCs)** 17

Table S2: **Recruitment summary by sites and overall** 19

Table S3: **Unsolicited AEs reported after administration of the vaccines or placebo** 20

Table S4: **Laboratory abnormalities**. 21

Table S5: **HIV-1 infectious molecular clones (IMCs) used in VIA** 21

Table S6**: Cross-clade HIV inhibition in vitro** 22

Annex 1: **The Study Protocol**

Materials and Methods

Vaccines

The ChAdOx1.tHIVconsv1 (C1) vaccine was manufactured, formulated and vialed under Good Manufacturing Practice (GMP) conditions at Advaxia Biologics (formerly Advent) s.r.l., Pomezia, Italy and was diluted in formulation buffer to $1{\cdot}3x10^{11}$ virus particles (vp)/ml (75 vp/infectious unit). The MVA.tHIVconsv3 (M3) and MVA.tHIVconsv4 (M4) vaccines were manufactured, formulated and vialed under GMP conditions by IDT Biologika GmbH, Dessau-Roβlau, Germany and were diluted in formulation buffer to $3{\cdot}4x10^{8}$ plaque-forming units (PFU)/ml for M3 and $1{\cdot}8x10^{8}$ PFU/ml for M4. All vaccines were stored ≤-60 °C until use.

Peptides

The HIVconsvX peptides used in this trial were the same set as used in all clinical studies testing the HIVconsvX strategy.[1-4] Briefly, 90% pure 15-mer peptides overlapping by 11 amino acids (15/11 aa) (C001-C401) (GenScript Biotech, Oxford, UK) matching the two mosaics were reconstituted to 40 mg/ml in DMSO and diluted to working stock solutions of 4 mg/ml in PBS. For ELISPOT and ICS assays, peptides were divided into ten pools, P1 to P10, containing between 34-47 mostly paired peptides of mosaic 1 and 2 in the same pool in R10 (RPMI 1460 supplemented with 10% FBS, 2 mM L-glutamine, 1 mM sodium pyruvate, 10 mM HEPES and 1% penicillin/streptomycin; Sigma Aldrich) and used at final concentration of 1.5 μg/ml each. GenScript Biotech pooled the peptides. For peptide mapping, 2x-concentrated stocks of individual peptide pairs/peptides were prepared in peptide plates. A pool of Flu/EBV/CMV peptides consisting of 32 previously defined $CD8^+$ T-cell epitopes (CEF; NIH AIDS Research and Reference Program) was reconstituted in DMSO and used at a final concentration of 1 μg/ml as a positive control. All peptide stocks and plates were stored at -80 °C until use.

Isolation and cryopreservation of PBMC

Blood was drawn into heparinized vacutainers (Becton Dickinson) and peripheral blood mononuclear cells (PBMC) were isolated within six hours and either used fresh or frozen for later analyses. After overnight freezing at -80 °C using Nalgene® Mr Frosty containers (Sigma-Aldrich), cryovials were transferred the next morning or the latest within 48 hours into the liquid nitrogen vapour for long-term storage.

Fresh IFN-γ ELISPOT Assay

Fresh *ex vivo* IFN-γ ELISPOT assays were carried out at each of the CRCs as described previously.[10] Peptide stimuli were tested in triplicates, while the no-peptide background had six wells. The median number of SFU in no-peptide wells was subtracted from test wells and the results were expressed as the median (IQR) net SFU/10^6 PBMC. Each plate had an internal control with an NK cell line aiming to give 200 SFU per well. Electronic records for all plates are retained. Both the clinical and laboratory teams were blinded for the vaccine vs. placebo allocation.

Short-term cell lines (STCL) and peptide mapping

STCL and peptide mapping were described previously.[1] Briefly, cryopreserved PBMC were thawed, and an equal volume of R10 plus 50 U/ml benzonase nuclease (Novagen, Paris, France) warmed to 37 °C was added dropwise. Cells were set up at 1-3x10^6 cells/ml in R10 supplemented with 25 ng/ml IL-7 (R&D Systems) and expanded using positive peptide pools identified in the fresh ELISPOT assay for ten days to establish STCL. Cultures were supplemented with 100 IU/ml IL-2 (R&D Systems) on day three, and IL-2 plus fresh culture medium on day seven. On day ten, the cells were washed three times with culture medium, re-suspended in 1 ml of medium and placed with loose lids at 37 °C, 5% CO_2 for 48 hours to rest before an IFN-γ ELISPOT assay restimulating with individual peptide pairs. The ELISPOT assay input was 40,000 cells/well.

Flow cell cytometry

ICS was described before.[1] Cryopreserved PBMC were thawed, resuspended in R10 medium at 10^6 cells/ml and labelled with anti-CD107a-FITC (Biolegend), stimulated with personalized pool of positive peptide pairs identified in the mapping (1·5 μg/ml of each peptide) or 1 μg/ml SEB (positive control) or R10 (negative control) and 1 μg/ml of anti-CD28 and anti-CD49d for 2 hours at 37 °C, 5% CO_2.

Then, 10 µg/ml of GolgiPlug (BD Biosciences) and 0·7 µl/ml of GolgiStop (BD Biosciences) was added for a further 17 hours and the cells were stained with a dead-cell marker (LIVE/DEAD Fixable Aqua stain; Invitrogen) followed by incubation with anti-CD14, CD19 and CD16-BV510 (Biolegend), anti-CD4-PE/Cy7 (BioLegend), anti-CD3-PE/Cy5 (Biolegend), anti-CD8-BV650 (Biolegend) antibodies, permeabilized with 200 µl of Cytofix/Cytoperm solution (BD Biosciences), stained with anti-IFN-γ-V450 (BD Biosciences), anti-TNF-α–PE-Dazzle (Biolegend), anti-IL-2 PE (BD Biosciences), CCL-3-APC (Miltenyi Biotec) fixed in 1% paraformaldehyde and acquired on a BD Fortessa flow cytometer (BD Biosciences). Data analysis was performed using FlowJo (Tree Star) and SPICE (NIAID) software.

As described previously,[10] for memory assays, cells were incubated with 100 µl of a master mix of anti-membrane marker mAbs containing LIVE/DEAD fixable aqua stain, anti-CD14, anti-CD19 and anti-CD16-BV510, anti-CD4 PE/Cy7 anti-CCR7 BV421, anti-CD8 BV650, anti-CD45RA FITC, and anti-CD27 APC (all from Biolegend), permeabilised with Cytofix/Cytoperm, then stained with anti-CD3 PE/Cy5, anti-IFN-γ PE/Dazzle (Biolegend), anti-IL-2 PE and anti-TNF-α APC/Cy7) and analyzed as above.

Virus inhibition assay (VIA)

VIA was performed on frozen PBMC in a blinded fashion as described previously.[1,5] Briefly, cryopreserved PBMC were thawed and the $CD4^+$ and $CD8^+$ T cells were polyclonally antigen-nonspecifically expanded by culture for seven days in R10 supplemented with IL-2 at 50 IU/ml and 0·5 µg/ml bi-specific antibodies (generously provided by J. Wong, Harvard Medical School, Boston, MA, US). Cultures were doubled in volume with fresh R10/IL-2 on days three and six. Culture with bi-specific anti-CD3/CD8 or anti-CD3/CD4 antibodies yielded >90% pure $CD4^+$ or $CD8^+$ T-cell populations, respectively. Expanded $CD4^+$ T cells were infected at a multiplicity of infection of 0·1 with eight separate HIV-1 IMCs, each engineered with the *Renilla reniformis* luciferase (*Luc*R) reporter gene and cultured for three days in R10/IL-2. Autologous $CD8^+$ T cells cultured in parallel were then added at 1:1 effector/target (v/v) ratio and cocultured for an additional five days. Virus replication was quantified by detecting the luciferase enzyme activity in the cell lysates using Renilla-Glo™ Luciferase Assay System (Promega Ltd., UK). Luminescence was measured using a Tecan Infinite M200Pro plate reader (Tecan Ltd., Switzerland) and expressed in relative light units (RLU). RLU data were expressed as $\log_{10}$ values with $\log_{10}$ inhibition mediated by $CD8^+$ T-cells calculated by subtraction of RLU values of $CD4^+/CD8^+$ T-cell co-cultures from RLU values of infected $CD4^+$ T-cell cultures alone. (Relative $\log_{10}$ inhibition=[-$\log_{10}$(CD4+CD8 RLU/CD4 RLU)]. For each trial subject, $CD4^+$ T cells were expanded from the pre-vaccination time point D0 only to act as a common target T-cell population with $CD8^+$ T cells as effectors expanded from D0, D35 and D308. Pre-vaccination $CD8^+$ T-cells served as background controls. The VIA was reproducible by repeated testing across three clinical trial centres in Kenya, Uganda and the United Kingdom, with no significant differences in RLU values or calculated $\log_{10}$ inhibition values across centres.[5]

Method references

1. Borthwick N, Fernandez N, Hayes PJ, et al. Safety and immunogenicity of the ChAdOx1-MVA-vectored conserved mosaic HIVconsvX candidate T-cell vaccines in HIV-CORE 005.2: an open-label, dose-escalation, first-in-man phase 1 trial in adults living without HIV-1 in the UK. *Lancet Microbe* In press.
2. Murakoshi H, Zou C, Kuse N, et al. CD8+ T cells specific for conserved, cross-reactive Gag epitopes with strong ability to suppress HIV-1 replication *Retrovirology* 2018; **15**: 46.
3. Ondondo B, Murakoshi H, Clutton G, et al. Novel Conserved-region T-cell Mosaic Vaccine With High Global HIV-1 Coverage Is Recognized by Protective Responses in Untreated Infection. *Mol Ther* 2016; **24**(4): 832-42.
4. Zou C, Murakoshi H, Kuse N, et al. Effective Suppression of HIV-1 Replication by Cytotoxic T Lymphocytes Specific for Pol Epitopes in Conserved Mosaic Vaccine Immunogens. *J Virol* 2019; **93**: e02142-18.
5. Fernandez N, Hayes P, Makinde J, et al. Assessment of a diverse panel of transmitted/founder HIV-1 infectious molecular clones in a luciferase based CD8 T-cell mediated viral inhibition assay. *Front Immunol* 2022; **13**: 1029029.

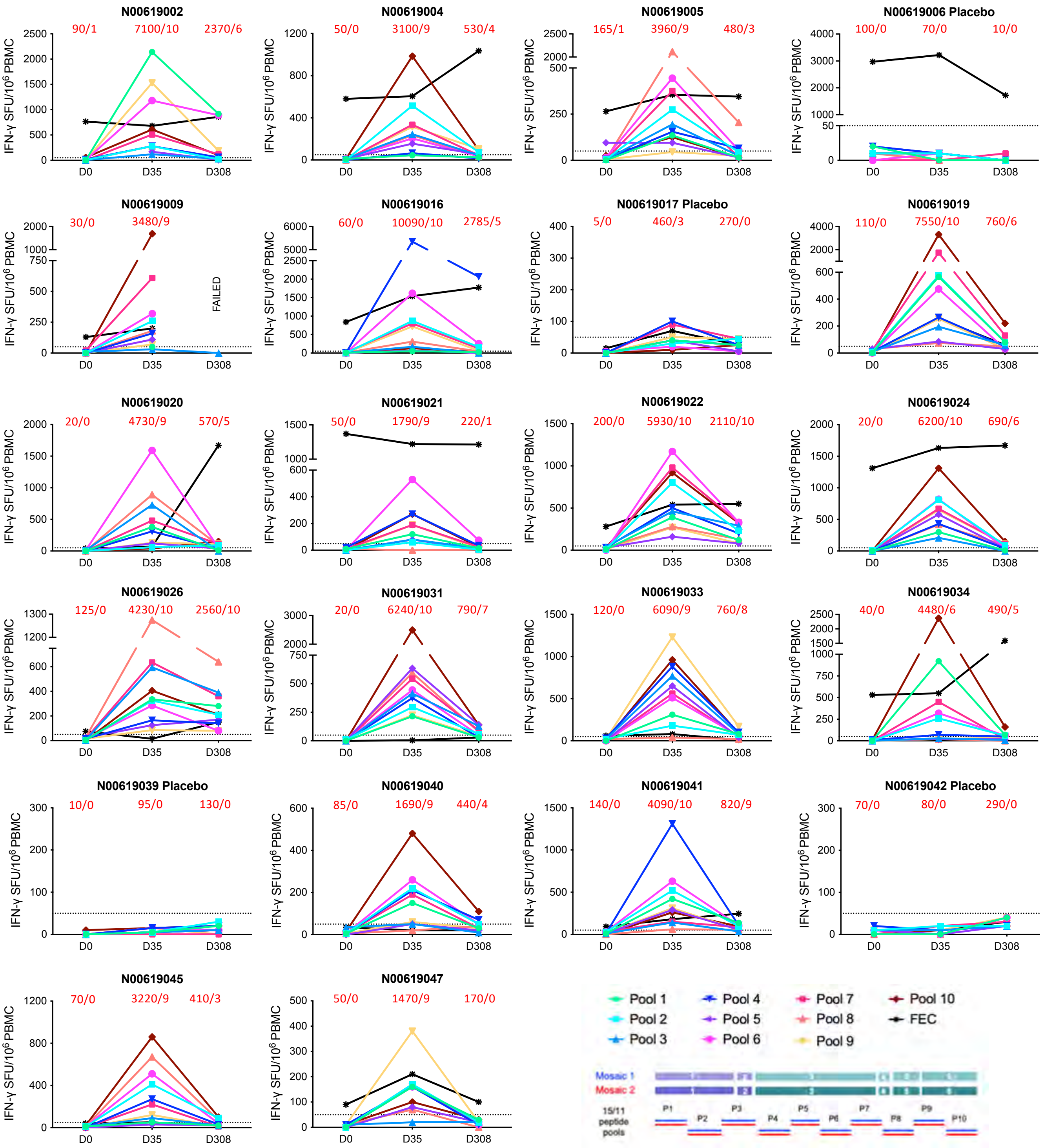

Figure S1A: **Fresh IFN-γ ELISPOT responses shown for individual study subjects (MUL)**
In HIV-CORE 006, 71 healthy HIV-1/2-negative low-risk adults in Eastern and Southern Specific received either vaccine regimen C1-M3M4 or placebo on D0 and D28, and their HIVconsvX-specific T-cell frequencies were determined using an IFN-γ ELISPOT assay and HIVconsvX peptide pools P1-P10 corresponding to the two mosaics 1 and 2 of the vaccines (bottom right). Each graph corresponds to a study individual (ID above) and provides frequencies of HIVconsvX-specific T cells before (D0), at peak responses (D35), and at the end of the study (D308). The red numbers above show the total frequencies (sum of ten pools)/number of responding HIVconsvX pools.

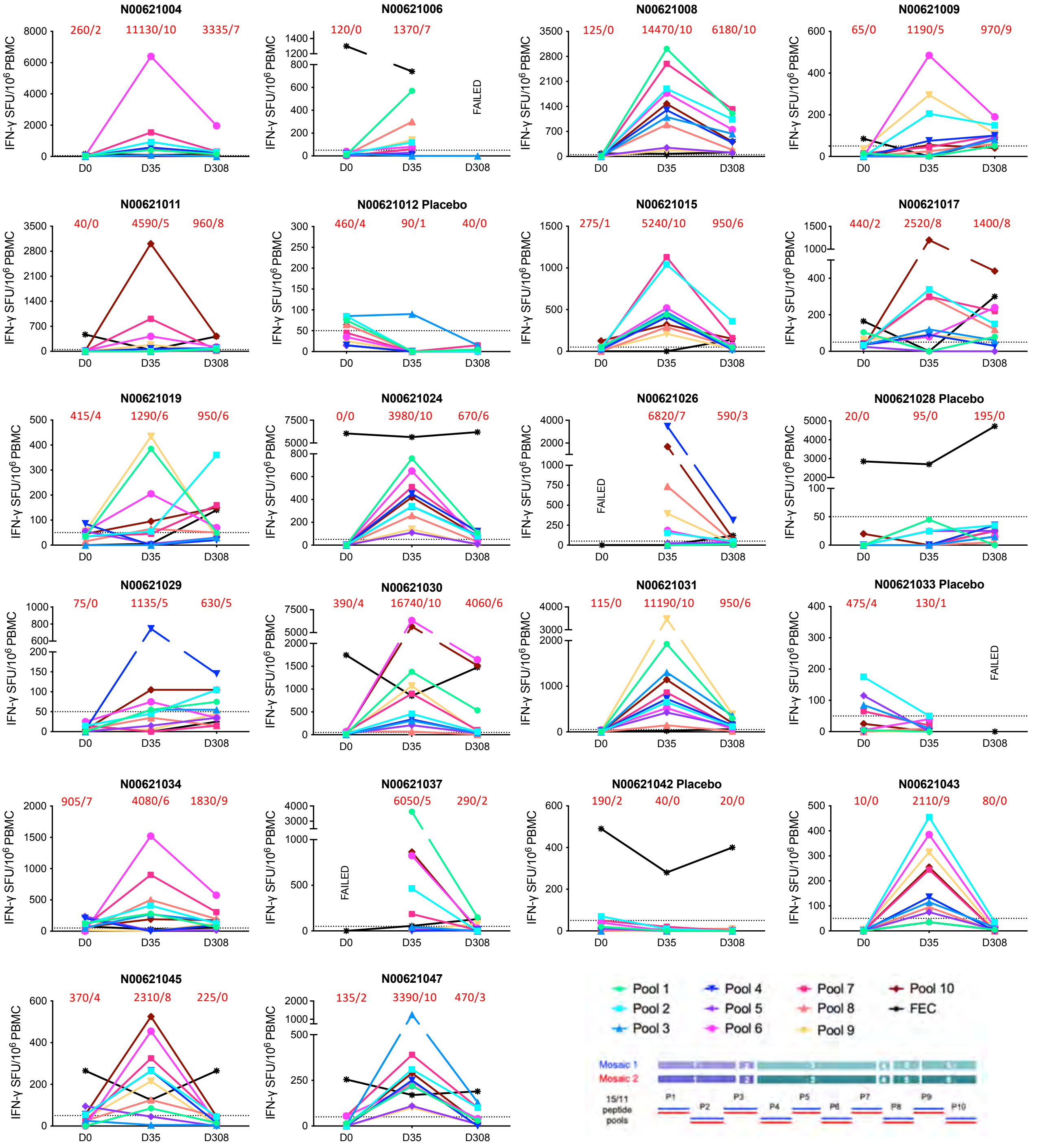

Figure S1B: **Fresh IFN-γ ELISPOT responses shown for individual study subjects (KWTRP)** Continued.

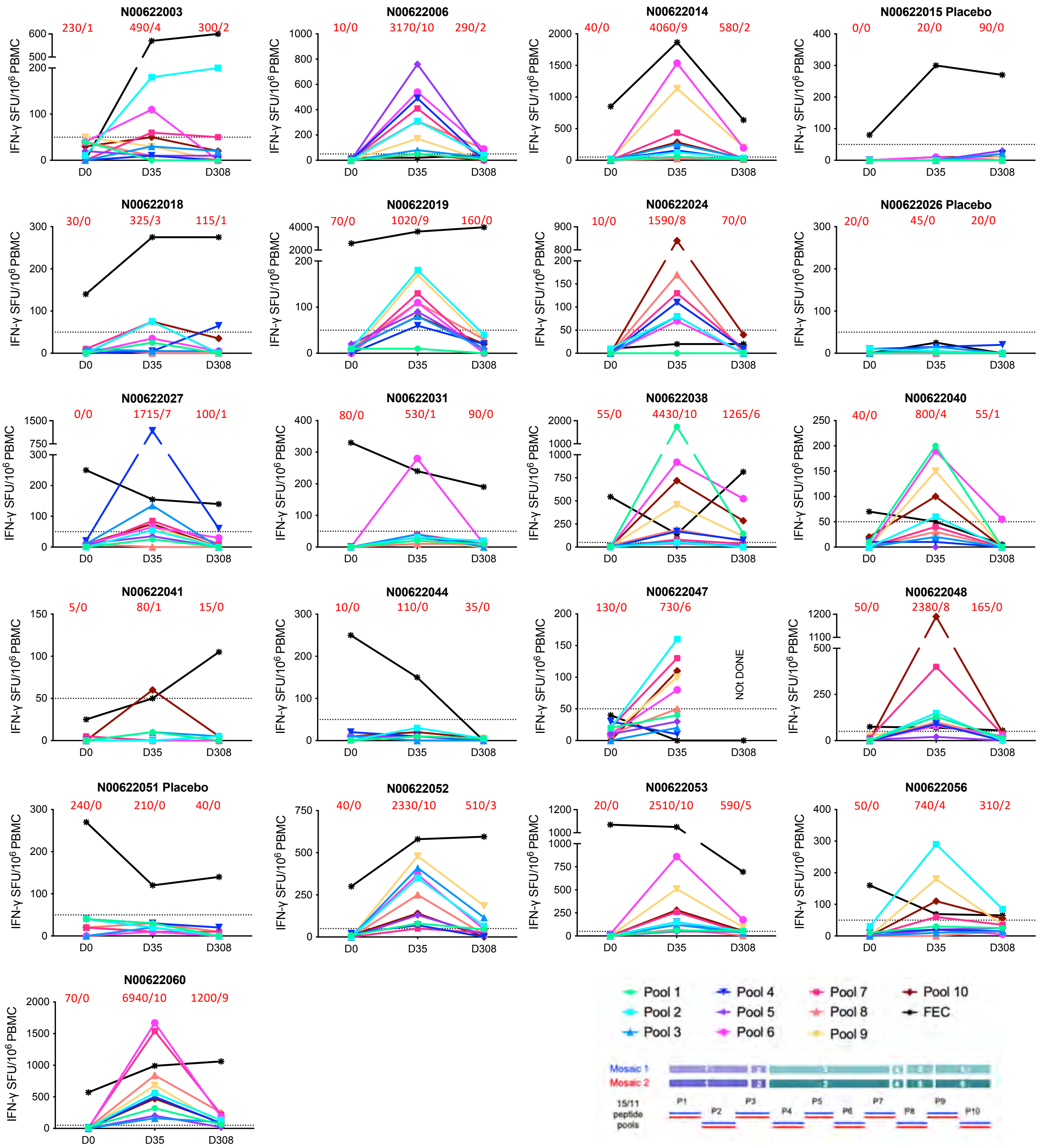

Figure S1C: **Fresh IFN-γ ELISPOT responses shown for individual study subjects (KAVI-ICR)** Continued.

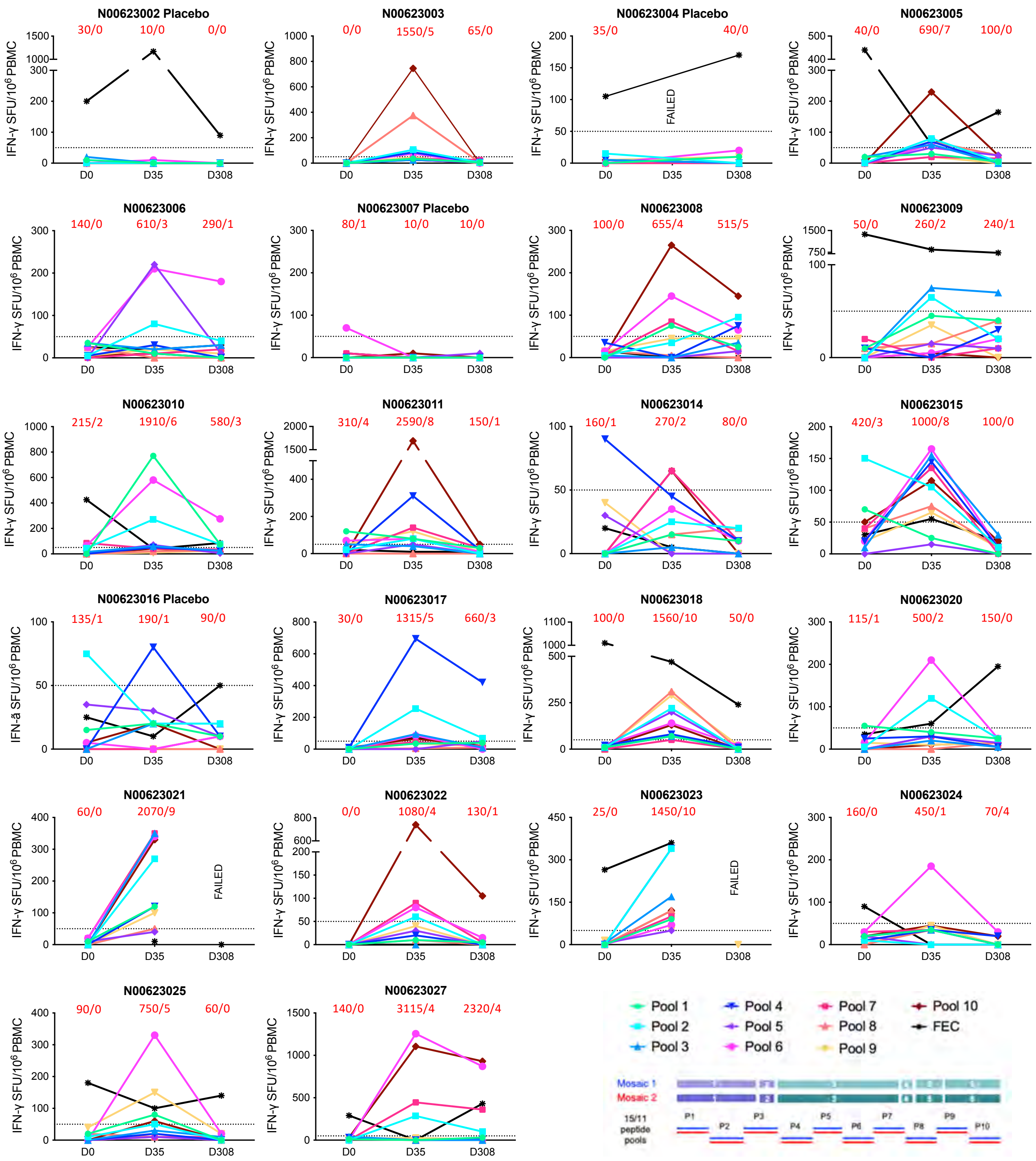

Figure S1D: **Fresh IFN-γ ELISPOT responses shown for individual study subjects (CFHRZ)** Continued.

MUL			N00619002	N00619004	N006190005	N00619009	N00619021	N00619022	N00619026	N00619033	N00619034	N00619039	N00619041	N00619042
		HLA-A	29:02/66:01	02:01/30:02	02:02/02:02	02:01/30:02	03:01/68:02	29:01/68:02	66:02/68:02	74:01/74:01	02:01/30:02	29:0129:02	01:01/74:03	36:01/74:01
		HLA-B	07:02/57:03	08:01/15:03	51:01/58:02	42:01/45:01	07:02/49:01	53:01/56:01	13:02/13:02	58:02/58:02	49:01/57:02	37:01/81:01	39:10/81:01	45:01/53:01
Peptide Sequences		HLA-C	07:01/07:02	02:10/07:01	06:02/16:01	16:01/17:01	07:01/7:02	01:02/04:01	06:02/06:02	06:02/06:02	07:01/18:02	04:01/07:01	04:01/12:03	04:01/06:02
C003 NLQGQMVHQAISPRT/C004 NAQGQMVHQALSPRT	Gag1	P1	1038	100	713	125	0	ND	0	100	63	1288	ND	313
C005 QMVHQAISPRTLNAW/C006 QMVHQALSPRTLNAW			3063	313	250	200	0	ND	13	75	2925	700	ND	250
C007 QAISPRTLNAWVKVI/C008 QALSPRTLNAWVKVV			1288	0	213	100	1513	ND	138	400	675	513	ND	200
C011 NAWVKVIEEKAFSPE/C012 NAWVKVVEEKAFSPE			475	63	863	63	0	ND	0	263	113	538	ND	213
C013 KVIEEKAFSPEVIPM/C014 KVVEEKAFSPEVIPM			588	1738	300	163	13	ND	0	213	213	163	ND	0
C015 EKAFSPEVIPMFTAL/C016 EKAFSPEVIPMFSAL			4663	1088	0	13	313	ND	0	0	50	138	ND	50
C017 SPEVIPMFTALSEGA/C018 SPEVIPMFSALSEGA			0	4350	388	175	0	ND	0	0	0	563	ND	100
C027 NTMLNTVGGHQAAMQ/C028 NMMLNIVGGHQAAMQ			188	0	775	163	0	ND	0	0	125	300	ND	150
C039 AAEWDRVHPVHAGPI/C040 AAEWDRLHPVHAGPI			0	0	675	263	400	ND	0	0	350	450	ND	288
C041 DRVHPVHAGPIAPGQ/C042 DRLHPVHAGPIPPGQ			200	350	950	188	0	ND	0	138	250	450	ND	225
C043 PVHAGPIAPGQMREP/C044 PVHAGPIPPGQMREP			0	1025	350	113	0	ND	138	488	275	200	ND	238
C047 PGQMREPRGSDIAGT		P2	219	2913	0	50	0	63	863	100	113	0	0	156
C048 REPRGSDIAGTTSNL/C049 REPRGSDIAGTTSTL			256	4963	0	25	0	0	700	87	63	44	0	0
C062 PVGDIYKRWIILGLN/C063 PVGEIYKRWIIMGLN			294	3188	0	263	0	188	250	175	663	19	2594	19
C064 IYKRWIILGLNKIVR/C065 IYKRWIIMGLNKIVR			1019	1513	0	238	0	588	163	75	688	19	0	69
C068 GLNKIVRMYSPVSIL/C069 GLNKIVRMYSPTSIL			844	388	175	75	0	1488	288	900	138	269	331	144
C070 IVRMYSPVSILDIRQ/C071 IVRMYSPTSILDIKQ			381	0	0	238	0	238	0	0	150	0	0	0
C072 YSPVSILDIRQGPKE/C073 YSPTSILDIKQGPKE			381	0	88	0	0	538	0	812	138	269	0	56
C074 SILDIRQGPKEPFRD/C075 SILDIKQGPKEPFRD			1931	0	250	25	0	0	0	362	0	231	0	94
C078 PKEPFRDYVDRFFKT/C079 PKEPFRDYVDRFYKT			81	0	300	75	0	738	925	0	13	169	169	194
C080 FRDYVDRFFKTLRAE/C081 FRDYVDRFYKTLRAE			606	188	313	13	0	138	88	25	275	0	1569	94
C082 VDRFFKTLRAEQATQ/C083 VDRFYKTLRAEQASQ			869	11338	2575	163	0	725	1513	975	1538	94	4869	119
C084 FKTLRAEQATQEVKN/C085 YKTLRAEQASQEVKN			94	6863	1563	0	0	700	713	150	900	0	0	31
C086 RAEQATQEVKNWMTD/C087 RAEQASQEVKNWMTE			281	0	0	38	0	638	800	0	25	144	0	0
C088 ATQEVKNWMTDTLLV/C089 ASQEVKNWMTETLLV			906	0	0	0	0	400	288	175	0	0	0	0
C092 MTDTLLVQNANPDCK//C093 MTETLLVQNANPDCK		P3	531	1938	375	0	425	0	0	0	0	119	0	0
C094 LLVQNANPDCKTILR/C095 LLVQNANPDCKTILK			556	2725	125	0	0	0	356	850	0	181	225	0
C100 ILRALGPGATLEEMM/C101 ILKALGPAATLEEMM			844	2738	0	44	838	350	0	0	0	31	325	0
C102 LGPGATLEEMMTACQ/C103 LGPAATLEEMMTACQ			606	2738	0	0	75	0	419	0	0	0	0	0
C111/CGKEGHIAKNCRAPR/C112/CGKEGHLARNCRAPR	Gag2		544	38	0	56	238	775	856	0	75	281	0	0
C113 GHIAKNCRAPRKRGC/C114 GHLARNCRAPRKKGC			556	663	1113	0	425	225	469	0	0	469	238	0
C115 KNCRAPRKRGCWKCG/C116 RNCRAPRKKGCWKCG			856	188	125	69	13	463	0	75	0	156	250	87.5
C129 ANFLGKIWPSHKGRP/C130 ANFLGKIWPSNKGRP			1344	625	1950	619	25	13	1831	1138	0	106	100	0
C131 GKIWPSHKGRPGNFL/C132 GKIWPSNKGRPGNFP			1206	0	0	19	0	538	69	813	0	19	0	0
C135 GRPGNFLQSRPEPTA/C136 GRPGNFPQSRPEPSA			844	2975	0	6	0	38	0	738	138	56	300	0
C139 LPGKWKPKMIGGIGG/C140 LPGRWKPKMIGGIGG	Pol3	P4	0	0	0	69	175	ND	1113	275	175	644	200	ND
C141 WKPKMIGGIGGFIKV			725	1044	594	744	63	ND	863	513	263	956	338	ND
C142 MIGGIGGFIKVKQYD/C143 MIGGIGGFIKVRQYD			0	0	0	656	350	ND	525	1038	525	431	100	ND
C144 IGGFIKVKQYDQILI/C145 IGGFIKVRQYDQIPI			0	0	406	231	338	ND	163	838	175	319	88	ND
C146 IKVKQYDQILIEICG/C147 IKVRQYDQIPIEICG			0	0	1531	206	413	ND	413	863	288	0	138	ND
C148 QYDQILIEICGKKAI/C149 QYDQIPIEICGHKAI			0	0	0	31	188	ND	513	213	313	0	0	ND
C150 ILIEICGKKAIGTVL/C151 IPIEICGHKAIGTVL			0	0	169	131	50	ND	275	488	113	0	63	ND
C154 KAIGTVLVGPTPVNI/C155 KAIGTVLIGPTPVNI			0	0	756	81	0	ND	838	0	288	306	188	ND
C156 TVLVGPTPVNIIGRN/C157 TVLIGPTPVNIIGRN			113	0	794	106	113	ND	925	300	375	669	150	ND
C158 GPTPVNIIGRNMLTQ/C159 GPTPVNIIGRNLLTQ			0	0	0	6	0	ND	650	825	738	194	3938	ND
C160 VNIIGRNMLTQLGCT/C161 VNIIGRNLLTQIGCT			0	0	144	44	238	ND	525	538	250	206	25	ND
C162 GRNMLTQLGCTLNFP/C163 GRNLLTQIGCTLNFP			0	0	0	44	238	ND	113	263	263	44	38	ND
C166 GCTLNFPISPIDTVP/C167 GCTLNFPISPIETVP			250	0	0	6	1925	ND	25	88	125	0	175	ND
C168 NFPISPIDTVPVTLK/C169 NFPISPIETVPVKLK			238	0	0	556	2563	ND	425	213	100	0	1450	ND
C170 SPIDTVPVTLKPGMD/C171 SPIETVPVKLKPGMD			0	0	0	1369	0	ND	213	0	25	0	5200	ND
C172 TVPVTLKPGMDGPRV/C173 TVPVKLKPGMDGPKV			0	0	0	56	0	ND	338	188	138	519	250	ND
C174 TLKPGMDGPRVKQWP/C175 KLKPGMDGPKVKQWP			0	0	0	0	0	ND	100	175	263	0	300	ND
C176 GMDGPRVKQWPLTEE/C177 GMDGPKVKQWPLTEE			0	0	0	231	0	ND	88	0	175	0	225	ND
C178 PRVKQWPLTEEKIKA/C179 PKVKQWPLTEEKIKA			0	0	0	94	88	ND	175	0	0	144	338	ND
C182 TEEKIKALTEICKEM/C183 TEEKIKALVEICTEM		P5	369	406	450	106	550	0	950	200	0	681	0	81
C184 IKALTEICKEMEKEG/C185 IKALVEICTEMEKEG			94	406	563	106	325	0	938	575	50	306	0	144
C186 TEICKEMEKEGKITK/C187 VEICTEMEKEGKISK			419	169	400	169	300	0	1175	88	25	131	0	94
C188 KEMEKEGKITKIGPE/C189 TEMEKEGKISKIGPE			0	6	2100	56	438	0	1338	25	50	306	0	19
C190 KEGKITKIGPENPYN/C191 KEGKISKIGPENPYN			0	31	1950	19	88	0	588	13	13	219	575	194
C192 ITKIGPENPYNTPIF/C193 ISKIGPENPYNTPVF			331	44	538	69	0	0	1150	200	13	331	0	44
C194 GPENPYNTPIFAIKK/C195 GPENPYNTPVFAIKK			0	0	150	81	75	0	1213	475	25	131	0	0
C196 PYNTPIFAIKKKDST/C197 PYNTPVFAIKKKDST			156	4894	350	206	38	0	1238	713	25	0	2563	181
C198 PIFAIKKKDSTKWRK/C199 PVFAIKKKDSTRWRK			0	8069	913	231	600	163	1550	1125	163	494	5125	94
C200 IKKKDSTKWRKLVDF/C201 IKKKDSTRWRKLVDF			469	319	175	306	763	0	1663	525	50	331	0	119
C202 DSTKWRKLVDFRELN/C203 DSTRWRKLVDFRELN			606	81	825	431	600	0	1538	375	138	0	0	156
C204 WRKLVDFRELNKKTQ/C205 WRKLVDFRELNKRTQ			0	5681	2325	269	475	0	1413	838	213	0	2900	231
C206 VDFRELNKKTQDFWE/C207 VDFRELNKRTQDFWE			0	5594	738	356	175	0	825	0	13	456	775	106
C208 ELNKKTQDFWEVQLG/C209 ELNKRTQDFWEVQLG			269	106	50	94	0	0	600	813	25	394	713	69
C210 KTQDFWEVQLGIPHP/C211 RTQDFWEVQLGIPHP			144	1869	0	31	88	0	825	738	13	206	0	31
C212 FWEVQLGIPHPAGLK/C213 FWEVQLGIPHPSGLK			0	906	513	44	0	0	1088	850	150	6	0	106
C214 QLGIPHPAGLKKKKS/C215 QLGIPHPSGLKKKRS			0	0	538	144	38	0	425	838	38	0	0	206
C216 PHPAGLKKKKSVTVL/C217 PHPSGLKKKRSVTVL			0	0	1000	131	200	0	913	650	0	0	0	81
C218 GLKKKKSVTVLDVGD/C219 GLKKKRSVTVLDVGD			156	0	2263	244	1438	0	1075	638	0	244	413	144
C220 KKSVTVLDVGDAYFS/C221 KRSVTVLDVGDAYFS		P6	531	0	338	0	188	0	1244	0	250	63	ND	25
C222 TVLDVGDAYFSVPLD			31	0	363	0	375	0	1544	0	388	125	ND	0
C224 VGDAYFSVPLDESFR/C225 VGDAYFSVPLDKDFR			569	0	550	6	1113	0	1481	381	88	138	ND	313
C226 YFSVPLDESFRKYTA/C227 YFSVPLDKDFRKYTA			344	0	875	6	450	0	1594	206	100	88	ND	0
C228 PLDESFRKYTAFTIP/C229 PLDKDFRKYTAFTIP			519	0	175	6	2488	0	431	0	250	150	ND	13
C230 SFRKYTAFTIPSINN/C231 DFRKYTAFTIPSTNN			756	0	888	69	3325	0	269	0	588	138	ND	0
C232 YTAFTIPSINNETPG/C233 YTAFTIPSTNNETPG			506	0	850	0	313	0	206	0	0	0	ND	100
C236 INNETPGIRYQYNVL/C237 TNNETPGVRYQYNVL			619	0	250	0	450	0	969	0	150	0	ND	0
C238 TPGIRYQYNVLPQGW/C239 TPGVRYQYNVLPMGW			294	2625	275	444	1150	0	1644	1456	300	388	ND	38
C240 RYQYNVLPQGWKGSP/C241 RYQYNVLPMGWKGSP			469	1600	513	369	350	0	644	1156	188	300	ND	263
C242 NVLPQGWKGSPAIFQ/C243 NVLPMGWKGSPAIFQ			406	0	1238	631	725	7788	956	0	163	263	ND	50
C244 QGWKGSPAIFQSSMT/C245 MGWKGSPAIFQCSMT			394	0	488	0	650	0	919	231	325	225	ND	0
C246 GSPAIFQSSMTKILE/C247 GSPAIFQCSMTKILE			406	0	363	119	225	0	856	0	13	138	ND	13
C254 FRAKNPEIVIYQYMD/C255 FRKQNPDIVIYQYMD			0	0	400	0	25	0	931	0	113	0	ND	13
C256 NPEIVIYQYMDDLYV/C257 NPDIVIYQYMDDLYI			0	4600	1150	644	875	563	406	0	2363	238	ND	0
C258 VIYQYMDDLYVGSDL/C259VIYQYMDDLYIGSDL			0	5325	1438	944	850	800	906	0	2513	0	ND	0
C260 YMDDLYVGSDLEIGQ/C261 YMDDLYIGSDLEIGQ			0	2650	438	0	1188	0	344	0	25	25	ND	0
C268 RAKIEELREHLLKWG/C269RTKIEELRQHLLRWG		P7	1813	0	125	0	0	0	2175	325	0	0	0	0
C282 FLWMGYELHPDRWTV/C283 FHWMGYELHPDKWTV			0	513	163	0	0	906	0	275	0	369	419	13
C290 PIQLPEKESWTVNDI/C291 PIVLPEKDSWTVNDI			0	1325	0	0	367	231	25	325	0	0	6	0
C292 PEKESWTVNDIQKLI/C293 PEKDSWTVNDIQKLV			0	5213	138	0	67	119	425	250	0	0	631	0
C294 SWTVNDIQKLIGKLN/C295 SWTVNDIQKLVGKLN			0	11050	613	438	392	319	238	300	0	31	2769	38
C298 KLIGKLNWASQIY/C299 KLVGKLNWASQIY			0	3850	2413	625	0	431	0	0	0	0	69	0
C300 AEIQKQGQDQWTYQI/C301 AEIQKQGQGQWTYQI	Pol4	P8	346	0	0	63	519	375	1563	344	281	188	838	81
C302 KQGQDQWTYQIYQEP/C303 KQGQGQWTYQIYQEP			0	0	25	0	356	213	2550	431	219	163	0	206
C304 DQWTYQIYQEPFKNL/C305 GQWTYQIYQEPYKNL			371	8563	200	75	381	50	575	206	319	625	3413	0
C306 YQIYQEPFKNLKTGK/C307 YQIYQEPYKNLKTGK			196	12538	213	150	356	0	738	431	81	813	0	44
C318 KCQLKGEAMHGQVDC/C319 KCQLKGEAIHGQVDC	Pol5		896	0	0	25	494	163	138	94	219	238	0	56
C334 QEFGIPYNPQSQGVV	Pol6	P9	0	125	681	44	481	563	1300	231	600	588	963	256
C335 IPYNPQSQGVVESMN			131	0	269	0	156	738	1400	219	263	175	725	194
C336 PQSQGVVESMNKELK/C337 PQSQGVVESMNNELK			281	0	0	6	0	388	813	6	200	75	175	281
C338 GVVESMNKELKKIIG/C339 GVVESMNNELKKIIG			244	75	0	0	206	538	813	56	563	0	450	56
C340 SMNKELKKIIGQVRE/C341 SMNNELKKIIGQVRD			769	88	144	0	506	588	588	0	400	50	0	131
C342 ELKKIIGQVREQAEH/C343 ELKKIIGQVRDQAEH			19	163	181	0	531	513	1525	569	0	750	400	56
C344 IIGQVREQAEHLKTA/C345 IIGQVRDQAEHLKTA			169	300	294	0	0	350	1488	94	413	313	788	369
C346 VREQAEHLKTAVQMA/C347 VRDQAEHLKTAVQMA			0	113	106	19	0	713	1113	119	325	338	250	494
C348 AEHLKTAVQMAVFIH/C349 AEHLKTAVQMAVLIH			0	0	0	56	406	113	763	406	400	325	425	156
C350 KTAVQMAVFIHNFKR/C351 KTAVQMAVLIHNFKR			531	0	544	0	0	238	438	944	200	238	588	269
C352 QMAVFIHNFKRKGGI/C353 QMAVLIHNFKRRGGI			419	8363	406	19	244	525	475	5406	1000	438	3575	281
C354 FIHNFKRKGGIGGYS/C355 LIHNFKRRGGIGGYS			456	6475	556	156	119	388	300	2919	50	350	575	269
C358 GGIGGYSAGERIIDI/C359 GGIGGYSAGERIVDI			0	0	0	0	206	0	1300	481	0	0	1000	0
C362 GERIIDIIATDIQTK/C363 GERIVDIIATDIQTR			0	50	0	94	1119	38	113	394	88	388	550	231
C368 QTKELQKQITKIQNF/C369 QTRELQKQIIKIQNF		P10	1113	0	0	113	196	525	0	550	150	556	0	6
C370 LQKQITKIQNFRVYY/C371 LQKQIIKIQNFRVYY			4475	0	0	1363	3083	725	438	1338	113	244	1038	0
C372 ITKIQNFRVYYRDSR/C373 IIKIQNFRVYYRDSR			4438	231	188	800	0	838	163	2413	1425	619	450	19
C374 QNFRVYYRDSRDPIW/C375 QNFRVYYRDSRDPLW			513	0	913	100	0	800	0	613	475	669	1438	0
C376 VYYRDSRDPIWKGPA/C377 VYYRDSRDPLWKGPA			188	0	313	0	0	638	75	850	0	169	0	0
C378 DSRDPIWKGPAKLLW/C379 DSRDPLWKGPAKLLW			200	0	838	0	0	888	0	625	0	0	0	0
C380 PIWKGPAKLLWKGEG/C381 PLWKGPAKLLWRGEG			100	0	0	100	0	2875	0	613	63	569	0	6
C382 GPAKLLWKGEGAVVI/C383 GPAKLLWRGEGAVVI			113	0	25	100	121	1950	0	363	138	344	0	0
C384 LLWKGEGAVVIQDNS/C385 LLWRGEGAVVIQDNS			700	0	0	263	783	2388	525	525	125	681	13	19
C386 GEGAVVIQDNSDIKV/C387 GEGAVVIQDNSEIKV			863	0	0	213	133	1213	0	850	238	831	363	0
C388 VVIQDNSDIKVVPRR/C389 VVIQDNSEIKVVPRR			650	0	0	0	0	850	0	638	ND	169	700	0
C390 DNSDIKVVPRRKVKI/C391 DNSEIKVVPRRKAKI			513	469	563	0	0	1050	0	1088	150	319	0	0
C392 IKVVPRRKVKIIKDY/C393 IKVVPRRKAKIIRDY			300	0	350	0	308	1938	200	1063	0	181	950	0
C394 PRRKVKIIKDYGKQM/C395 PRRKAKIIRDYGKQM			125	0	650	1488	758	1125	0	1388	0	294	0	0
C396 VKIIKDYGKQMAGAD/C397 AKIIRDYGKQMAGDD			350	0	613	263	533	1425	0	3288	200	269	0	44
C398 KDYGKQMAGADCVAG/C399 RDYGKQMAGDDCVAS			100	0	263	300	58	888	300	525	163	381	0	0

Figure S2A: **Volunteers' HLA class I and peptide pair mapping by STCL (MUL)**

Study subject IDs (placebo highlighted yellow) and their genetically determined HLA class I alleles are shown at the top. Frozen PBMCs D35 were thawed and restimulated *in vitro* for ten days separately with each peptide pool, rested and incubated with deconvoluted mosaic peptide pairs (left column) in an IFN-γ ELISPOT assay. The HIV-1 conserved regions and peptide pool numbers are indicated. The numbers show the frequencies of IFN-γ SFU/10^6 STCL. Responses ≥750 SFU/10^6 STCL are heat-scaled for convenience.

KEMRI			N00621004	N00621006	N00621008	N00621011	N00621015	N00621017	N00621024	N00621026	N00621030	N00621031	N00621034	N00621037	N00621042	N00621045	N00621047
		HLA-A	02:02/36:01	11:01/29:02	36:01/36:01	30:02/32:01	01:01/01:01	23:01/30:04	66:02/68:02	29:01/29:02	02:01/30:02	01:09/36:01	23:17/68:02	30:01/32:01	01:02/03:01	02:05/30:01	02:02/68:02
		HLA-B	53:01/73:01	40:06/58:02	58:01/58:01	53:01/81:01	44:03/49:01	07:02/58:02	07:02/58:01	39:10/42:01	07:02/57:03	44:15/53:01	45:01/53:01	08:01/57:03	18:01/58:01	37:01/42:01	13:02/58:02
Peptide Sequences		HLA-C	04:01/15:05	06:02/15:02	07:18/07:18	03:04/08:04	06:02/07:01	06:02/07:02	07:02/07:18	12:03/17:01	04:01/18:02	04:01/04:07	04:01/06:02	03:04/07:01	02:02/03:02	06:02/17:01	06:02/06:02
C003 NLQGQMVHQAISPRT/C004 NAQGQMVHQALSPRT	Gag1	P1	413	0	338	113	0	0	0	300	0	50	0	375	0	0	25
C005 QMVHQAISPRTLNAW/C006 QMVHQALSPRTLNAW			1175	0	3263	188	0	0	0	875	150	75	625	1125	0	0	113
C007 QAISPRTLNAWVKVI/C008 QALSPRTLNAWVKVV			0	63	2150	25	0	0	513	1050	113	100	700	25	0	0	0
C011 NAWVKVIEEKAFSPE/C012 NAWVKVVEEKAFSPE			25	238	63	100	0	0	2075	275	63	438	0	550	0	0	0
C015 EKAFSPEVIPMFTAL/C016 EKAFSPEVIPMFSAL			100	150	38	38	0	0	0	200	338	0	0	3250	0	0	0
C017 SPEVIPMFTALSEGA/C018 SPEVIPMFSALSEGA			613	325	200	38	650	0	0	225	0	50	1150	2450	0	213	613
C023 EGATPQDLNTMLNTV/C024 EGATPQDLNMMLNIV			138	400	0	100	0	0	813	0	425	150	0	150	0	0	0
C037 INEEAAEWDRVHPVH/C038 INEEAAEWDRLHPVH			225	313	13	63	650	0	0	625	0	25	ND	0	163	0	1138
C039 AAEWDRVHPVHAGPI/C040 AAEWDRLHPVHAGPI			2425	2200	0	163	0	0	325	25	300	2500	ND	150	63	0	375
C041 DRVHPVHAGPIAPGQ/C042 DRLHPVHAGPIPPGQ			5725	0	0	0	0	0	25	250	38	100	ND	ND	13	0	0
C047 PGQMREPRGSDIAGT		P2	0	0	0	119	0	0	0	0	1375	0	0	0	0	0	0
C048 REPRGSDIAGTTSNL/C049 REPRGSDIAGTTSTL			0	0	0	44	288	19	281	0	1925	56	0	0	0	0	0
C050 GSDIAGTTSNLQEQI/C051 GSDIAGTTSTLQEQI			38	0	0	0	638	0	169	0	1725	0	0	0	0	319	0
C054 SNLQEQIGWMTSNPP/C055 STLQEQIGWMTNNPP			100	0	0	81	538	19	344	0	788	0	875	0	106	706	0
C056 EQIGWMTSNPPIPVG/C057 EQIGWMTNNPPIPVG			213	0	0	56	663	6	0	0	1363	0	0	0	0	319	75
C058 WMTSNPPIPVGDIYK/C059 WMTNNPPIPVGEIYK			0	0	0	0	0	6	0	0	1288	0	0	0	0	0	0
C060 NPPIPVGDIYKRWII/C061 NPPIPVGEIYKRWII			2800	0	0	0	0	19	6	0	1513	0	1800	0	69	0	0
C062 PVGDIYKRWIILGLN/C063 PVGEIYKRWIIMGLN			300	713	0	206	1238	6	469	0	2688	319	0	0	181	0	0
C064 IYKRWIILGLNKIVR/C065 IYKRWIIMGLNKIVR			488	75	1488	369	2463	6	1269	0	1363	0	425	ND	0	0	950
C066 WIILGLNKIVRMYSP/C067 WIIMGLNKIVRMYSP			1788	0	338	31	763	6	544	225	1963	0	250	ND	131	319	0
C068 GLNKIVRMYSPVSIL/C069 GLNKIVRMYSPTSIL			350	738	3375	81	138	6	1019	0	1763	44	4700	ND	0	281	0
C070 IVRMYSPVSILDIRQ/C071 IVRMYSPTSILDIKQ			138	0	938	156	538	31	169	0	2188	0	3500	ND	0	0	138
C072 YSPVSILDIRQGPKE/C073 YSPTSILDIKQGPKE			138	238	0	56	113	0	0	0	2788	0	300	ND	0	19	0
C074 SILDIRQGPKEPFRD/C075 SILDIKQGPKEPFRD			0	0	0	69	0	6	0	0	1313	0	0	ND	0	0	0
C076 IRQGPKEPFRDYVDR/C077 IKQGPKEPFRDYVDR			113	0	0	6	0	0	119	0	2788	94	ND	ND	0	0	450
C078 PKEPFRDYVDRFFKT/C079 PKEPFRDYVDRFYKT			138	0	2813	69	0	6	0	0	3788	0	ND	ND	44	0	88
C080 FRDYVDRFFKTLRAE/C081 FRDYVDRFYKTLRAE			538	0	2188	0	0	6	244	0	4438	244	ND	ND	156	319	0
C082 VDRFFKTLRAEQATQ/C083 VDRFYKTLRAEQASQ			113	663	700	6	1338	6	2269	0	7913	369	ND	ND	31	0	0
C084 FKTLRAEQATQEVKN/C085 YKTLRAEQASQEVKN			0	0	313	0	588	0	19	0	3638	0	ND	ND	0	0	0
C086 RAEQATQEVKNWMTD/C087 RAEQASQEVKNWMTE			113	0	0	31	0	0	194	0	1738	19	ND	ND	6	0	0
C088 ATQEVKNWMTDTLLV/C089 ASQEVKNWMTETLLV			38	0	0	44	113	19	19	0	1188	0	ND	ND	81	0	288
C090 VKNWMTDTLLVQNAN/C091 VKNWMTETLLVQNAN			213	0	0	0	538	6	0	825	963	0	ND	ND	0	0	0
C092 MTDTLLVQNANPDCK/C093 MTETLLVQNANPDCK		P3	1100	0	100	31	0	0	0	0	0	63	200	0	0	0	0
C094 LLVQNANPDCKTILR/C095 LLVQNANPDCKTILK			350	0	350	6	250	0	144	0	1400	163	400	25	0	0	0
C096 NANPDCKTILRALGP/C097 NANPDCKTILKALGP			413	0	763	0	0	0	244	106	0	0	400	200	0	0	131
C098 DCKTILRALGPGATL/C099 DCKTILKALGPAATL			263	0	6300	106	275	0	169	0	0	0	7900	300	0	0	0
C100 ILRALGPGATLEEMM/C101 ILKALGPAATLEEMM			2625	0	4625	519	50	0	6	31	0	63	6025	0	6	0	0
C109 KCFNCGKEGHIAKNC/C110 KCFNCGKEGHLARNC			1375	0	363	0	13	0	256	56	50	0	0	ND	0	0	0
C113 GHIAKNCRAPRKRGC/C114 GHLARNCRAPRKKGC	Gag2		1050	0	0	6	338	56	31	206	0	0	250	ND	94	0	0
C127 NERQANFLGKIWPSH/C128 TERQANFLGKIWPSN			0	0	0	0	413	0	1181	0	0	250	175	ND	0	0	0
C129 ANFLGKIWPSHKGRP/C130 ANFLGKIWPSNKGRP			725	0	0	0	0	119	7131	206	0	2050	425	ND	6	0	0
C131 GKIWPSHKGRPGNFL/C132 GKIWPSNKGRPGNFP			0	0	138	0	38	0	3506	431	0	375	0	ND	0	325	0
C139 LPGKWKPKMIGGIGG/C140 LPGRWKPKMIGGIGG	Pol3	P4	281	488	0	188	0	13	225	144	150	31	175	750	0	369	38
C141 WKPKMIGGIGGFIKV			144	650	0	175	294	13	588	119	13	406	0	4725	0	0	188
C142 MIGGIGGFIKVKQYD/C143 MIGGIGGFIKVRQYD			181	800	994	113	269	13	788	44	325	1794	25	2050	50	0	25
C144 IGGFIKVKQYDQILI/C145 IGGFIKVRQYDQIPI			31	750	0	113	0	25	338	0	900	456	0	2225	0	0	0
C146 IKVKQYDQILIEICG/C147 IKVRQYDQIPIEICG			1244	775	94	13	0	13	475	244	825	0	75	325	0	269	0
C148 QYDQILIEICGKKAI/C149 QYDQIPIEICGHKAI			219	425	0	38	306	63	288	69	1325	69	25	4000	0	331	100
C150 ILIEICGKKAIGTVL/C151 IPIEICGHKAIGTVL			31	250	294	38	0	13	188	0	1800	31	125	3275	0	319	413
C152 ICGKKAIGTVLVGPT/C153 ICGHKAIGTVLIGPT			0	450	6	88	0	13	438	0	0	0	0	1125	0	169	200
C154 KAIGTVLVGPTPVNI/C155 KAIGTVLIGPTPVNI			19	450	2381	213	494	0	100	0	125	6	0	ND	0	1306	350
C156 TVLVGPTPVNIIGRN/C157 TVLIGPTPVNIIGRN			0	850	1856	150	169	38	338	69	625	0	0	ND	0	919	0
C158 GPTPVNIIGRNMLTQ/C159 GPTPVNIIGRNLLTQ			119	788	0	788	369	25	113	169	500	0	0	ND	0	31	175
C160 VNIIGRNMLTQLGCT/C161 VNIIGRNLLTQIGCT			156	663	56	125	0	25	163	119	950	0	0	ND	0	256	0
C162 GRNMLTQLGCTLNFP/C163 GRNLLTQIGCTLNFP			0	463	256	75	131	88	713	0	1325	131	0	ND	88	0	0
C164 LTQLGCTLNFPISPI/C165 LTQIGCTLNFPISPI			0	425	206	138	0	13	0	94	2075	56	125	ND	0	0	0
C166 GCTLNFPISPIDTVP/C167 GCTLNFPISPIETVP			0	425	319	100	0	63	263	294	2175	0	7700	ND	38	231	0
C168 NFPISPIDTVPVTLK/C169 NFPISPIETVPVKLK			956	188	44	100	0	38	488	494	725	6	10100	ND	0	244	0
C170 SPIDTVPVTLKPGMD/C171 SPIETVPVKLKPGMD			2519	200	694	300	119	63	488	1944	0	0	100	ND	0	0	0
C178 PRVKQWPLTEEKIKA/C179 PKVKQWPLTEEKIKA			0	425	0	0	944	25	138	0	1600	0	ND	ND	50	0	0
C184 IKALTEICKEMEKEG/C185 IKALVEICTEMEKEG		P5	200	44	0	75	313	6	469	50	0	44	550	0	275	0	0
C186 TEICKEMEKEGKITK/C187 VEICTEMEKEGKISK			63	94	6	75	50	6	569	75	163	44	550	850	375	0	0
C198 PIFAIKKKDSTKWRK/C199 PVFAIKKKDSTRWRK			138	0	0	125	0	6	344	150	100	231	625	2925	138	0	0
C200 IKKKDSTKWRKLVDF/C201 IKKKDSTRWRKLVDF			175	69	0	100	0	6	469	25	50	0	25	1675	150	0	0
C208 ELNKKTQDFWEVQLG/C209 ELNKRTQDFWEVQLG			88	31	0	38	0	0	219	0	1625	0	550	ND	225	219	0
C212 FWEVQLGIPHPAGLK/C213 FWEVQLGIPHPSGLK			150	0	0	0	0	44	519	50	1525	169	25	ND	113	0	0
C216 PHPAGLKKKKSVTVL/C217 PHPSGLKKKRSVTVL			75	0	0	13	225	0	1019	200	1200	0	ND	ND	200	656	0
C218 GLKKKKSVTVLDVGD/C219 GLKKKRSVTVLDVGD			125	19	0	100	0	0	469	0	3600	144	ND	ND	0	106	0
C220 KKSVTVLDVGDAYFS/C221 KRSVTVLDVGDAYFS		P6	0	244	2244	125	613	400	350	44	40	0	100	0	0	0	719
C222 TVLDVGDAYFSVPLD			0	494	1219	13	1100	0	213	0	55	0	400	0	0	56	0
C224 VGDAYFSVPLDESFR/C225 VGDAYFSVPLDKDFR			0	81	969	125	438	0	425	94	96	0	300	0	38	356	381
C226 YFSVPLDESFRKYTA/C227 YFSVPLDKDFRKYTA			0	6	369	13	225	0	0	969	192	0	12350	0	0	0	0
C228 PLDESFRKYTAFTIP/C229 PLDKDFRKYTAFTIP			63	269	0	375	325	1700	1175	494	32	0	50	250	13	281	0
C230 SFRKYTAFTIPSINN/C231 DFRKYTAFTIPSTNN			0	756	2081	225	1175	650	0	419	33	100	450	1650	0	319	1419
C232 YTAFTIPSINNETPG/C233 YTAFTIPSTNNETPG			13	344	969	88	75	413	125	219	28	0	550	0	0	669	1031
C238 TPGIRYQYNVLPQGW/C239 TPGVRYQYNVLPMGW			17663	369	2131	125	700	288	0	444	33	625	825	325	38	106	56
C240 RYQYNVLPQGWKGSP/C241 RYQYNVLPMGWKGSP			10763	294	0	0	775	0	0	644	53	850	150	0	38	294	69
C242 NVLPQGWKGSPAIFQ/C243 NVLPMGWKGSPAIFQ			113	381	219	100	375	0	25	294	50	100	2500	0	0	219	606
C244 QGWKGSPAIFQSSMT/C245 MGWKGSPAIFQCSMT			0	469	0	125	475	1200	175	19	31	0	75	2100	0	131	406
C246 GSPAIFQSSMTKILE/C247 GSPAIFQCSMTKILE			0	81	894	50	100	0	0	144	34	0	0	ND	0	156	494
C248 IFQSSMTKILEPFRA/C249 IFQCSMTKILEPFRK			0	231	856	138	350	488	0	169	56	150	0	ND	0	56	31
C252 ILEPFRAKNPEIVIY/C253 ILEPFRKQNPDIVIY			0	81	169	13	0	938	50	69	17	0	225	ND	0	0	19
C254 FRAKNPEIVIYQYMD/C255 FRKQNPDIVIYQYMD			0	106	1006	0	0	163	475	444	26	0	0	ND	88	0	0
C256 NPEIVIYQYMDDLYV/C257 NPDIVIYQYMDDLYI			13	269	5444	75	0	0	350	0	90	25	4275	ND	163	569	981
C258 VIYQYMDDLYVGSDL/C259VIYQYMDDLYIGSDL			188	644	5394	213	25	0	225	219	108	0	3625	ND	0	1194	694
C262 LYVGSDLEIGQHRAK/C263 LYIGSDLEIGQHRTK		P7	13	0	269	0	0	656	0	319	663	38	163	875	0	469	0
C264 SDLEIGQHRAKIEEL/C265 SDLEIGQHRTKIEEL			113	50	0	6	0	356	281	0	1138	38	300	825	88	706	619
C266 IGQHRAKIEELREHL/C267IGQHRTKIEELRQHL			100	0	1119	0	0	44	0	0	0	75	50	900	125	556	44
C268 RAKIEELREHLLKWG/C269RTKIEELRQHLLRWG			0	0	3356	6	331	306	944	0	900	275	1675	ND	238	606	19
C270 EELREHLLKWGFTTP/C271 EELRQHLLRWGFTTP			88	0	469	31	1206	181	6	219	275	13	175	350	163	631	0
C272 EHLLKWGFTTPDKKH/C273 QHLLRWGFTTPDKKH			88	0	856	44	131	0	244	319	0	138	0	ND	163	119	0
C276 TTPDKKHQKEPPFLW/C277 TTPDKKHQKEPPFHW			25	0	781	0	0	206	131	0	375	0	25	ND	0	0	0
C280 KEPPFLWMGYELHPD/C281 KEPPFHWMGYELHPD			0	0	169	0	0	319	181	0	800	350	825	ND	188	981	456
C282 FLWMGYELHPDRWTV/C283 FHWMGYELHPDKWTV			88	0	81	56	531	119	0	0	1950	50	750	ND	50	569	0
C284 GYELHPDRWTVQPIQ/C285 GYELHPDKWTVQPIV			63	0	869	19	0	231	0	69	200	38	0	ND	138	406	319
C288 WTVQPIQLPEKESWT/C289 WTVQPIVLPEKDSWT			0	0	444	6	0	31	156	0	300	88	1325	ND	88	1419	0
C290 PIQLPEKESWTVNDI/C291 PIVLPEKDSWTVNDI			0	0	1019	0	0	769	131	69	1000	13	150	ND	0	581	0
C294 SWTVNDIQKLIGKLN/C295 SWTVNDIQKLVGKLN			175	0	781	56	0	31	56	269	3025	388	125	ND	13	1169	906
C296 NDIQKLIGKLNWASQ/C297 NDIQKLVGKLNWASQ			0	0	2344	6	0	44	431	69	3375	163	575	ND	50	669	1656
C298 KLIGKLNWASQIY/C299 KLVGKLNWASQIY			250	0	31	594	1131	519	0	419	2075	25	100	ND	0	681	919
C300 AEIQKQGQDQWTYQI/C301 AEIQKQGQGQWTYQI	Pol4	P8	494	350	225	0	failed	0	244	38	1533	0	300	0	0	644	425
C302 KQGQDQWTYQIYQEP/C303 KQGQGQWTYQIYQEP			244	825	263	44	failed	0	494	0	1483	0	613	0	0	0	175
C304 DQWTYQIYQEPFKNL/C305 GQWTYQIYQEPYKNL			644	650	2550	194	failed	200	844	0	308	175	3400	300	0	0	938
C306 YQIYQEPFKNLKTGK/C307 YQIYQEPYKNLKTGK			1081	175	2375	181	failed	0	2281	0	933	0	8675	0	0	0	738
C308 QEPFKNLKTGKYA/C309 QEPYKNLKTGKYA			0	225	0	131	failed	0	44	0	1208	0	150	400	0	469	75
C310 FNLPPIVAKEIVANC/C311 FNLPPVVAKEIVASC	Pol5		644	638	50	6	failed	0	44	0	2058	0	200	0	0	0	13
C312 PIVAKEIVANCDKCQ/C313 PVVAKEIVASCDKCQ			406	613	225	69	failed	0	0	0	2058	13	425	225	0	0	500
C314 KEIVANCDKCQLKGE/C315 KEIVASCDKCQLKGE			281	375	150	0	failed	0	219	88	1683	0	50	ND	0	0	275
C316 ANCDKCQLKGEAMHG/C317 ASCDKCQLKGEAIHG			381	0	400	6	failed	0	231	263	1458	13	50	ND	0	0	200
C318 KCQLKGEAMHGQVDC/C319 KCQLKGEAIHGQVDC			206	600	0	94	failed	0	619	288	1208	0	350	ND	0	0	625
C320 KGEAMHGQVDCSPGI/C321 KGEAIHGQVDCSPGI			0	238	0	144	failed	0	31	63	3033	0	25	ND	106	0	213
C322 MHGQVDCSPGIWQLD/C323 IHGQVDCSPGMWQLD			206	438	163	44	failed	13	206	63	758	0	0	ND	0	0	563
C324 VDCSPGIWQLDCTHL/C325 VDCSPGMWQLDCTHL			6	13	113	94	failed	13	0	0	1783	0	125	ND	0	0	0
C328 QLDCTHLEGKVILVA/C329 QLDCTHLEGKIILVA			0	275	0	0	failed	13	319	0	1333	113	50	ND	0	0	0
C332 GKVILVAVHVASGY/C333 GKIILVAVHVASGY			281	125	175	0	failed	0	544	0	2608	0	250	ND	0	0	63
C334 QEFGIPYNPQSQGVV	Pol6	P9	1004	144	0	0	269	0	69	88	413	388	900	0	56	450	850
C335 IPYNPQSQGVVESMN			379	319	281	38	494	6	206	325	1225	200	0	475	6	513	0
C336 PQSQGVVESMNKELK/C337 PQSQGVVESMNNELK			254	0	0	88	394	31	119	88	0	625	275	800	119	550	0
C338 GVVESMNKELKKIIG/C339 GVVESMNNELKKIIG			592	0	0	50	81	0	0	200	938	288	0	1025	0	350	1175
C340 SMNKELKKIIGQVRE/C341 SMNNELKKIIGQVRD			0	0	644	125	0	0	0	0	775	150	0	175	0	0	675
C342 ELKKIIGQVREQAEH/C343 ELKKIIGQVRDQAEH			142	6	0	175	0	0	469	0	1225	475	25	200	0	0	350
C344 IIGQVREQAEHLKTA/C345 IIGQVRDQAEHLKTA			179	19	0	238	269	0	169	0	1388	375	0	2375	0	1375	1300
C346 VREQAEHLKTAVQMA/C347 VRDQAEHLKTAVQMA			142	194	0	38	631	0	344	0	1650	275	50	ND	169	175	525
C348 AEHLKTAVQMAVFIH/C349 AEHLKTAVQMAVLIH			42	269	0	25	6	44	419	275	1200	275	125	ND	94	175	275
C350 KTAVQMAVFIHNFKR/C351 KTAVQMAVLIHNFKR			142	356	0	0	406	0	-6	0	800	488	625	ND	0	475	0
C352 QMAVFIHNFKRKGGI/C353 QMAVLIHNFKRRGGI			129	244	0	213	469	0	1019	725	2163	2100	600	ND	319	88	3500
C354 FIHNFKRKGGIGGYS/C355 LIHNFKRRGGIGGYS			679	206	0	50	44	0	344	175	600	1238	0	ND	44	663	0
C362 GERIIDIIATDIQTK/C363 GERIVDIIATDIQTR			0	0	0	125	1206	0	569	0	2900	825	0	ND	231	788	0
C364 IDIIATDIQTKELQK/C365 VDIIATDIQTRELQK			567	0	0	13	906	0	244	0	1900	150	150	ND	56	675	413
C366 ATDIQTKELQKQITK/C367 ATDIQTRELQKQIIK			467	0	0	38	431	0	294	0	1050	0	475	ND	431	88	0
C368 QTKELQKQITKIQNF/C369 QTRELQKQIIKIQNF		P10	50	0	969	625	1625	0	0	63	663	0	713	5000	0	488	281
C370 LQKQITKIQNFRVYY/C371 LQKQIIKIQNFRVYY			225	0	844	2088	1525	0	1331	263	2425	763	1338	5425	0	238	44
C372 ITKIQNFRVYYRDSR/C373 IIKIQNFRVYYRDSR			188	0	1419	2013	1700	0	56	0	425	450	688	575	31	1125	744
C374 QNFRVYYRDSRDPIW/C375 QNFRVYYRDSRDPLW			2063	0	619	238	1825	0	0	88	0	163	588	100	0	1238	106
C376 VYYRDSRDPIWKGPA/C377 VYYRDSRDPLWKGPA			1800	0	0	163	950	0	69	213	0	0	138	0	0	763	0
C378 DSRDPIWKGPAKLLW/C379 DSRDPLWKGPAKLLW			0	0	381	0	325	0	119	38	0	350	663	1675	0	0	481
C380 PIWKGPAKLLWKGEG/C381 PLWKGPAKLLWRGEG			0	0	2406	0	0	0	0	163	0	0	1913	350	0	725	1881
C382 GPAKLLWKGEGAVVI/C383 GPAKLLWRGEGAVVI			163	63	0	0	0	0	0	213	0	0	0	0	0	0	869
C384 LLWKGEGAVVIQDNS/C385 LLWRGEGAVVIQDNS			350	0	331	175	25	0	269	88	1425	0	263	ND	269	0	506
C386 GEGAVVIQDNSDIKV/C387 GEGAVVIQDNSEIKV			63	0	0	425	1450	0	0	0	275	50	88	ND	119	188	681
C388 VVIQDNSDIKVVPRR/C389 VVIQDNSEIKVVPRR			0	0	56	0	1750	25	0	138	0	125	113	ND	331	338	31
C390 DNSDIKVVPRRKVKI/C391 DNSEIKVVPRRKAKI			0	0	0	25	1075	0	0	0	0	275	138	ND	19	50	44
C392 IKVVPRRKVKIIKDY/C393 IKVVPRRKAKIIRDY			63	0	0	600	525	25	0	0	0	313	363	ND	0	950	919
C394 PRRKVKIIKDYGKQM/C395 PRRKAKIIRDYGKQM			0	0	0	50	0	0	169	788	1125	613	613	ND	0	1625	19
C396 VKIIKDYGKQMAGAD/C397 AKIIRDYGKQMAGDD			0	0	56	0	50	0	0	638	1925	1188	488	ND	0	663	0
C398 KDYGKQMAGADCVAG/C399 RDYGKQMAGDDCVAS			150	25	0	13	0	0	0	0	1200	50	0	ND	0	0	444
C400 KQMAGADCVAGRQDED/C401 KQMAGDDCVASRQDED			225	0	0	0	1025	0	0	213	900	0	113	ND	0	0	356

Figure S2B: **Volunteers' HLA class I and peptide pair mapping by STCL (KWTRP)**

KAVI-ICR			N00622003	N00622006	N00622014	N00622015	N00622019	N00622024	N00622026	N00622027	N00622044	N00622047	N00622048	N00622051
		HLA-A	01:01/02:01	02:05/68:02	02:01/02:01	03:01/29:02	02:01/02:01	30:01/66:01	23:01/30:01	02:05/68:02	03:01/03:01	36:01/66:01	30:02/68:02	30:04/36:01
		HLA-B	15:03/27:26	15:10/40:12	15:03/53:01	15:10/35:01	45:01/53:01	42:01/58:02	42:01/81:01	07:02/58:01	47:03/49:01	53:01/58:02	15:10/42:01	45:01/58:02
Peptide Sequences		HLA-C	02:02/02:10	03:04/04:04	02:10/04:01	03:04/04:01	04:01/16:01	06:02/17:01	17:01/18:01	07:01/07:02	07:01/07:01	06:02/06:02	03:04/17:01	08:02/16:01
C003 NLQGQMVHQAISPRT/C004 NAQGQMVHQALSPRT	Gag1	P1	125	63	0	0	0	1050	0	0	225	0	275	0
C005 QMVHQAISPRTLNAW/C006 QMVHQALSPRTLNAW			0	38	0	0	0	2388	0	225	188	0	425	0
C007 QAISPRTLNAWVKVI/C008 QALSPRTLNAWVKVV			13	25	138	0	0	1000	63	313	0	0	313	0
C009 PRTLNAWVKVIEEKA/C010 PRTLNAWVKVVEEKA			113	38	238	0	0	1050	0	0	0	0	100	0
C023 EGATPQDLNTMLNTV/C024 EGATPQDLNMMLNIV			175	50	0	0	0	775	25	0	25	0	638	0
C029 NTVGGHQAAMQMLKD/C030 NIVGGHQAAMQMLKE			100	38	500	0	0	1300	0	938	188	0	363	0
C039 AAEWDRVHPVHAGPI/C040 AAEWDRLHPVHAGPI			150	50	563	0	0	1400	0	0	125	0	188	0
C047 PGQMREPRGSDIAGT		P2	150	0	81	463	1394	0	0	0	0	0	56	0
C048 REPRGSDIAGTTSNL/C049 REPRGSDIAGTTSTL			138	0	319	75	2806	0	0	0	88	0	0	0
C052 AGTTSNLQEQIGWMT/C053 AGTTSTLQEQIGWMT			0	0	1056	0	0	0	0	0	25	0	0	0
C056 EQIGWMTSNPPIPVG/C057 EQIGWMTNNPPIPVG			100	0	0	0	0	0	0	0	38	1400	369	0
C062 PVGDIYKRWIILGLN/C063 PVGEIYKRWIIMGLN			0	4225	581	0	6256	0	0	0	0	3013	119	0
C064 IYKRWIILGLNKIVR/C065 IYKRWIIMGLNKIVR			688	788	919	0	3481	250	0	0	0	2638	0	0
C066 WIILGLNKIVRMYSP/C067 WIIMGLNKIVRMYSP			300	200	1494	0	1194	2288	0	0	263	613	119	0
C068 GLNKIVRMYSPVSIL/C069 GLNKIVRMYSPTSIL			150	13	1944	438	494	325	25	0	0	0	431	0
C070 IVRMYSPVSILDIRQ/C071 IVRMYSPTSILDIKQ			0	0	1344	0	656	0	0	81	0	0	31	0
C072 YSPVSILDIRQGPKE/C073 YSPTSILDIKQGPKE			88	0	1356	200	294	0	75	544	213	0	0	0
C078 PKEPFRDYVDRFFKT/C079 PKEPFRDYVDRFYKT			150	50	1631	63	419	0	0	0	125	0	119	0
C080 FRDYVDRFFKTLRAE/C081 FRDYVDRFYKTLRAE			700	0	1381	0	606	375	0	206	63	0	0	0
C082 VDRFFKTLRAEQATQ/C083 VDRFYKTLRAEQASQ			3375	0	481	0	369	1063	0	0	863	1688	81	0
C084 FKTLRAEQATQEVKN/C085 YKTLRAEQASQEVKN			538	0	1019	0	831	0	0	581	813	0	0	0
C086 RAEQATQEVKNWMTD/C087 RAEQASQEVKNWMTE			63	0	1444	0	281	0	0	781	338	0	519	0
C088 ATQEVKNWMTDTLLV/C089 ASQEVKNWMTETLLV			38	0	1319	150	544	0	150	206	0	0	0	0
C094 LLVQNANPDCKTILR/C095 LLVQNANPDCKTILK		P3	0	0	263	69	969	0	0	0	0	25	350	0
C119 RGCWKCGREGHQMKD/C120 KGCWKCGKEGHQMKD	Gag2		0	13	0	419	381	0	96	788	663	850	38	0
C129 ANFLGKIWPSHKGRP/C130 ANFLGKIWPSNKGRP			0	63	13	306	0	744	283	0	1150	3575	325	350
C131 GKIWPSHKGRPGNFL/C132 GKIWPSNKGRPGNFP			0	13	0	0	431	0	133	0	625	1450	0	0
C141 WKPKMIGGIGGFIKV	Pol3	P4	394	13	550	0	2481	779	163	644	238	613	0	550
C142 MIGGIGGFIKVKQYD/C143 MIGGIGGFIKVRQYD			431	13	138	131	1094	1517	125	0	263	713	163	688
C144 IGGFIKVKQYDQILI/C145 IGGFIKVRQYDQIPI			294	0	188	119	1194	2954	125	881	225	375	0	450
C146 IKVKQYDQILIEICG/C147 IKVRQYDQIPIEICG			169	113	450	0	1419	1642	0	431	388	738	363	613
C148 QYDQILIEICGKKAI/C149 QYDQIPIEICGHKAI			ND	0	388	206	1094	804	0	556	75	500	538	788
C150 ILIEICGKKAIGTVL/C151 IPIEICGHKAIGTVL			44	463	438	0	1081	792	0	231	300	563	100	488
C152 ICGKKAIGTVLVGPT/C153 ICGHKAIGTVLIGPT			69	38	338	0	1306	954	0	131	13	138	0	0
C154 KAIGTVLVGPTPVNI/C155 KAIGTVLIGPTPVNI			306	813	113	0	294	0	263	6094	13	300	0	0
C156 TVLVGPTPVNIIGRN/C157 TVLIGPTPVNIIGRN			419	688	625	119	494	604	0	5744	525	413	0	463
C158 GPTPVNIIGRNMLTQ/C159 GPTPVNIIGRNLLTQ			494	13	538	0	969	1854	125	194	213	975	0	513
C160 VNIIGRNMLTQLGCT/C161 VNIIGRNLLTQIGCT			31	63	450	0	631	1204	75	219	63	363	150	0
C162 GRNMLTQLGCTLNFP/C163 GRNLLTQIGCTLNFP			81	25	213	6	481	1617	0	456	0	0	588	250
C164 LTQLGCTLNFPISPI/C165 LTQIGCTLNFPISPI			44	13	438	0	469	2467	0	419	25	363	0	0
C166 GCTLNFPISPIDTVP/C167 GCTLNFPISPIETVP			94	50	225	106	469	767	125	356	113	238	0	13
C174 TLKPGMDGPRVKQWP/C175 KLKPGMDGPKVKQWP			231	13	0	0	244	1342	75	0	175	163	0	0
C176 GMDGPRVKQWPLTEE/C177 GMDGPKVKQWPLTEE			231	0	400	119	244	929	0	319	25	338	0	0
C178 PRVKQWPLTEEKIKA/C179 PKVKQWPLTEEKIKA			144	0	638	0	306	917	0	0	363	0	0	0
C184 IKALTEICKEMEKEG/C185 IKALVEICTEMEKEG		P5	69	25	0	119	0	1013	0	0	0	0	0	46
C188 KEMEKEGKITKIGPE/C189 TEMEKEGKISKIGPE			31	0	781	256	0	88	0	0	0	0	0	0
C196 PYNTPIFAIKKKDST/C197 PYNTPVFAIKKKDST			44	63	0	0	2075	213	0	0	0	200	0	0
C198 PIFAIKKKDSTKWRK/C199 PVFAIKKKDSTRWRK			31	388	0	69	3425	675	300	569	50	1738	31	0
C200 IKKKDSTKWRKLVDF/C201 IKKKDSTRWRKLVDF			106	38	69	206	0	788	88	281	0	438	0	8
C202 DSTKWRKLVDFRELN/C203 DSTRWRKLVDFRELN			419	663	0	144	0	788	288	0	775	0	0	0
C204 WRKLVDFRELNKKTQ/C205 WRKLVDFRELNKRTQ			231	7125	0	119	25	1038	50	0	550	0	0	133
C208 ELNKKTQDFWEVQLG/C209 ELNKRTQDFWEVQLG			231	13	0	206	25	900	0	0	0	38	0	0
C218 GLKKKKSVTVLDVGD/C219 GLKKKRSVTVLDVGD			31	213	0	0	0	1325	113	0	ND	0	606	733
C220 KKSVTVLDVGDAYFS/C221 KRSVTVLDVGDAYFS		P6	0	25	0	50	463	988	238	106	0	450	544	238
C222 TVLDVGDAYFSVPLD			206	100	256	400	500	1875	263	444	200	713	481	663
C224 VGDAYFSVPLDESFR/C225 VGDAYFSVPLDKDFR			119	150	244	150	1075	1988	488	256	363	675	1031	513
C226 YFSVPLDESFRKYTA/C227 YFSVPLDKDFRKYTA			44	138	556	225	538	2550	650	194	0	1075	1131	325
C228 PLDESFRKYTAFTIP/C229 PLDKDFRKYTAFTIP			81	175	381	463	713	2375	475	906	113	763	694	313
C230 SFRKYTAFTIPSINN/C231 DFRKYTAFTIPSTNN			1206	63	3131	563	588	1163	375	256	0	1138	781	425
C232 YTAFTIPSINNETPG/C233 YTAFTIPSTNNETPG			494	75	2569	263	375	588	275	544	225	625	744	338
C234 TIPSINNETPGIRYQ/C235 TIPSTNNETPGVRYQ			31	50	256	463	613	438	150	44	0	800	806	238
C236 INNETPGIRYQYNVL/C237 TNNETPGVRYQYNVL			0	38	119	150	263	325	450	519	63	588	294	438
C238 TPGIRYQYNVLPQGW/C239 TPGVRYQYNVLPMGW			31	113	769	225	1300	2200	225	406	875	813	656	425
C240 RYQYNVLPQGWKGSP/C241 RYQYNVLPMGWKGSP			181	225	644	50	1188	1988	363	531	788	513	1081	63
C242 NVLPQGWKGSPAIFQ/C243 NVLPMGWKGSPAIFQ			44	25	494	75	200	1013	500	444	288	0	1019	763
C244 QGWKGSPAIFQSSMT/C245 MGWKGSPAIFQCSMT			0	63	269	188	525	1625	238	444	0	0	806	125
C246 GSPAIFQSSMTKILE/C247 GSPAIFQCSMTKILE			0	38	81	288	625	1763	288	269	0	13	706	213
C248 IFQSSMTKILEPFRA/C249 IFQCSMTKILEPFRK			31	113	19	275	538	875	175	494	38	0	419	0
C250 SMTKILEPFRAKNPE/C251 SMTKILEPFRKQNPD			0	25	106	63	500	450	25	156	0	0	106	900
C254 FRAKNPEIVIYQYMD/C255 FRKQNPDIVIYQYMD			81	38	44	75	163	363	50	344	313	0	356	800
C256 NPEIVIYQYMDDLYV/C257 NPDIVIYQYMDDLYI			2081	875	6344	125	700	488	250	656	4238	88	381	0
C258 VIYQYMDDLYVGSDL/C259VIYQYMDDLYIGSDL			3969	1513	5681	175	725	1238	400	381	5163	0	269	300
C260 YMDDLYVGSDLEIGQ/C261 YMDDLYIGSDLEIGQ			56	13	456	138	650	1088	200	0	313	88	306	1075
C262 LYVGSDLEIGQHRAK/C263 LYIGSDLEIGQHRTK		P7	13	0	1369	0	0	0	194	0	0	0	0	246
C264 SDLEIGQHRAKIEEL/C265 SDLEIGQHRTKIEEL			138	0	1456	0	344	0	19	406	0	0	0	108
C266 IGQHRAKIEELREHL/C267IGQHRTKIEELRQHL			13	6	1119	0	0	0	219	0	0	0	138	433
C268 RAKIEELREHLLKWG/C269RTKIEELRQHLLRWG			125	0	1269	0	0	0	306	0	0	1688	0	0
C270 EELREHLLKWGFTTP/C271 EELRQHLLRWGFTTP			50	0	1106	0	0	0	156	0	0	0	0	0
C272 EHLLKWGFTTPDKKH/C273 QHLLRWGFTTPDKKH			0	0	1206	106	0	0	31	0	0	0	75	0
C274 KWGFTTPDKKHQKEP/C275 RWGFTTPDKKHQKEP			0	0	1294	0	0	0	94	0	0	0	113	171
C278 KKHQKEPPFLWMGYE/C279 KKHQKEPPFHWMGYE			0	506	1306	0	44	0	19	0	0	0	0	0
C280 KEPPFLWMGYELHPD/C281 KEPPFHWMGYELHPD			38	81	1306	0	169	0	206	0	0	100	0	0
C282 FLWMGYELHPDRWTV/C283 FHWMGYELHPDKWTV			225	31	1581	0	219	850	69	344	0	175	0	83
C284 GYELHPDRWTVQPIQ/C285 GYELHPDKWTVQPIV			38	19	1719	0	306	0	94	0	0	63	0	8
C286 HPDRWTVQPIQLPEK/C287 HPDKWTVQPIVLPEK			225	0	1744	0	906	0	119	0	0	75	13	208
C290 PIQLPEKESWTVNDI/C291 PIVLPEKDSWTVNDI			50	106	1131	0	481	0	0	0	0	0	213	0
C294 SWTVNDIQKLIGKLN/C295 SWTVNDIQKLVGKLN			0	281	631	231	3431	2225	69	494	0	75	0	0
C296 NDIQKLIGKLNWASQ/C297 NDIQKLVGKLNWASQ			88	94	481	0	1756	0	131	0	0	175	0	383
C298 KLIGKLNWASQIY/C299 KLVGKLNWASQIY			350	344	0	0	644	0	0	0	0	1425	2400	0
C300 AEIQKQGQDQWTYQI/C301 AEIQKQGQGQWTYQI	Pol4	P8	219	25	288	69	1025	838	38	19	0	931	56	281
C302 KQGQDQWTYQIYQEP/C303 KQGQGQWTYQIYQEP			81	13	0	431	738	613	163	756	0	556	181	231
C304 DQWTYQIYQEPFKNL/C305 GQWTYQIYQEPYKNL			694	0	363	219	2113	275	100	744	0	1156	0	0
C306 YQIYQEPFKNLKTGK/C307 YQIYQEPYKNLKTGK			44	25	1300	219	2238	863	238	294	375	494	0	0
C312 PIVAKEIVANCDKCQ/C313 PVVAKEIVASCDKCQ	Pol5		394	38	0	119	1038	400	13	0	0	469	69	206
C314 KEIVANCDKCQLKGE/C315 KEIVASCDKCQLKGE			281	0	63	356	900	713	0	94	0	281	219	381
C334 QEFGIPYNPQSQGVV	Pol6	P9	200	0	288	163	0	1463	0	0	100	663	319	638
C336 PQSQGVVESMNKELK/C337 PQSQGVVESMNNELK			38	13	63	0	156	263	88	456	438	275	906	575
C348 AEHLKTAVQMAVFIH/C349 AEHLKTAVQMAVLIH			138	25	0	188	244	1225	38	0	0	188	0	138
C352 QMAVFIHNFKRKGGI/C353 QMAVLIHNFKRRGGI			1413	25	513	600	1681	800	0	231	2763	963	469	0
C354 FIHNFKRKGGIGGYS/C355 LIHNFKRRGGIGGYS			650	50	550	975	1894	763	113	556	2125	600	1019	325
C360 GYSAGERIIDIIATD/C361 GYSAGERIVDIIATD			0	38	238	13	981	0	0	169	0	250	181	175
C362 GERIIDIIATDIQTK/C363 GERIVDIIATDIQTR			113	138	950	0	3681	925	113	194	175	925	331	550
C368 QTKELQKQITKIQNF/C369 QTRELQKQIIKIQNF		P10	0	100	1050	0	369	0	181	619	ND	181	81	475
C370 LQKQITKIQNFRVYY/C371 LQKQIIKIQNFRVYY			169	150	1000	244	681	38	119	594	ND	1606	44	575
C372 ITKIQNFRVYYRDSR/C373 IIKIQNFRVYYRDSR			131	138	675	131	819	1700	144	1031	ND	1019	481	850
C374 QNFRVYYRDSRDPIW/C375 QNFRVYYRDSRDPLW			319	63	1538	0	1819	2075	94	256	ND	1131	119	775
C376 VYYRDSRDPIWKGPA/C377 VYYRDSRDPLWKGPA			256	113	1663	94	806	725	256	281	ND	244	256	475
C378 DSRDPIWKGPAKLLW/C379 DSRDPLWKGPAKLLW			44	75	1513	19	981	0	94	0	ND	294	69	363
C380 PIWKGPAKLLWKGEG/C381 PLWKGPAKLLWRGEG			44	113	950	169	544	0	106	356	ND	394	131	475
C382 GPAKLLWKGEGAVVI/C383 GPAKLLWRGEGAVVI			0	50	188	169	619	1775	156	144	ND	169	6	625
C386 GEGAVVIQDNSDIKV/C387 GEGAVVIQDNSEIKV			56	38	1888	431	844	0	69	394	ND	206	269	413
C388 VVIQDNSDIKVVPRR/C389 VVIQDNSEIKVVPRR			69	38	0	56	1044	150	44	0	ND	0	69	513
C390 DNSDIKVVPRRKVKI/C391 DNSEIKVVPRRKAKI			331	63	0	144	1594	1275	56	281	ND	219	144	250
C392 IKVVPRRKVKIIKDY/C393 IKVVPRRKAKIIRDY			856	38	2300	169	1419	2150	0	0	ND	744	331	538
C394 PRRKVKIIKDYGKQM/C395 PRRKAKIIRDYGKQM			294	88	0	0	806	1525	206	0	ND	244	731	238
C396 VKIIKDYGKQMAGAD/C397 AKIIRDYGKQMAGDD			156	25	0	219	669	925	106	381	ND	69	94	138
C398 KDYGKQMAGADCVAG/C399 RDYGKQMAGDDCVAS			31	50	1888	456	369	750	119	0	ND	294	181	0
C400 KQMAGADCVAGRQDED/C401 KQMAGDDCVASRQDED			6	25	1188	0	394	0	69	0	ND	319	106	0

Figure S2C: **Volunteers' HLA class I and peptide pair mapping by STCL (KAVI-ICR)**

CFHRZ

Peptide Sequences			N00623003	N00623005	N00623006	N00623008	N00623010	N00623011	N00623015	N00623017	N00623018	N00623020	N00623021	N00623022	N00623023	N00623025	N00623027
		HLA-A	01:01/66:01	03:01/23:01	23:01/36:01	23:01/30:02	02:01/34:02	02:02/32:01	23:01/68:02	02:05/36:01	02:01/02:02	23:01/74:01	30:01/74:01	29:02/30:02	02:02/68:02	02:01/6:01	03:01/30:02
		HLA-B	39:10/53:01	15:03/42:01	41:01/53:01	45:01/57:03	35:01/57:02	15:16/45:01	15:10/53:01	53:01/58:01	15:03/51:01	14:02/35:01	45:01/53:01	08:01/18:01	14:01/15:16	49:01/3:01	14:02/35:01
		HLA-C	04:01/12:03	02:10/17:01	04:01/08:02	06:02/18:02	04:01/18:02	06:02/16:01	04:01/16:01	04:01/07:18	02:10/16:01	04:01/08:02	04:01/06:02	02:10/07:01	08:02/16:01	04:01/07:01	06:02/08:02
C001 PIVQNLQGQMVHQAI/C002 PIVQNAQGQMVHQAL	Gag1	P1	0	50	0	150	0	788	63	63	313	125	263	0	188	413	300
C005 QMVHQAISPRTLNAW/C006 QMVHQALSPRTLNAW			0	0	0	200	10263	138	425	1113	75	175	250	0	325	163	350
C007 QAISPRTLNAWVKVI/C008 QALSPRTLNAWVKVV			38	0	0	0	2338	275	500	350	538	125	375	0	0	138	288
C015 EKAFSPEVIPMFTAL/C016 EKAFSPEVIPMFSAL			288	200	13	325	25	388	250	1688	0	0	0	0	175	400	150
C017 SPEVIPMFTALSEGA/C018 SPEVIPMFSALSEGA			88	1000	0	38	0	363	225	263	88	250	338	0	463	563	263
C027 NTMLNTVGGHQAAMQ/C028 NMMLNIVGGHQAAMQ			313	200	150	150	0	0	613	463	100	200	138	0	413	900	238
C029 NTVGGHQAAMQMLKD/C030 NIVGGHQAAMQMLKE			288	0	0	0	0	538	25	388	525	175	7312*	0	188	350	163
C033 AMQMLKDTINEEAAE/C034 AMQMLKETINEEAAE			88	0	0	50	25	413	0	63	775	25	250	0	238	450	200
C043 PVHAGPIAPGQMREP/C044 PVHAGPIPPGQMREP			0	0	300	188	0	0	138	0	375	250	4575*	0	0	288	0
C052 AGTTSNLQEQIGWMT/C053 AGTTSTLQEQIGWMT		P2	138	150	0	38	13	1025	0	92	0	0	106	0	0	169	175
C058 WMTSNPPIPVGDIYK/C059 WMTNNPPIPVGEIYK			0	150	0	13	3588	0	0	0	0	250	0	31	0	0	725
C060 NPPIPVGDIYKRWII/C061 NPPIPVGEIYKRWII			963	75	744	0	6113	625	0	492	0	300	344	2306	0	994	650
C062 PVGDIYKRWIILGLN/C063 PVGEIYKRWIIMGLN			13	325	0	238	0	650	0	242	0	4775	144	2706	0	169	125
C064 IYKRWIILGLNKIVR/C065 IYKRWIIMGLNKIVR			238	200	0	1313	250	350	213	17	0	5000	331	81	38	156	225
C066 WIILGLNKIVRMYSP/C067 WIIMGLNKIVRMYSP			213	750	0	1013	288	375	588	0	0	275	0	0	0	556	88
C068 GLNKIVRMYSPVSIL/C069 GLNKIVRMYSPTSIL			313	275	0	75	188	7775	0	267	0	825	6	0	650	306	788
C070 IVRMYSPVSILDIRQ/C071 IVRMYSPTSILDIKQ			188	350	506	213	288	3550	0	92	0	250	444	106	250	0	375
C076 IRQGPKEPFRDYVDR/C077 IKQGPKEPFRDYVDR			188	325	131	88	63	1050	0	0	0	150	194	381	38	394	313
C078 PKEPFRDYVDRFFKT/C079 PKEPFRDYVDRFYKT			388	650	0	13	813	825	25	17	0	150	0	0	288	0	388
C080 FRDYVDRFFKTLRAE/C081 FRDYVDRFYKTLRAE			338	200	0	0	988	1825	2463	0	425	0	44	0	263	594	213
C082 VDRFFKTLRAEQATQ/C083 VDRFYKTLRAEQASQ			1038	275	156	13	3975	4050	2338	1767	2050	3800	994	2056	1613	4694	1313
C084 FKTLRAEQATQEVKN/C085 YKTLRAEQASQEVKN			713	25	231	0	675	150	488	217	0	800	0	631	113	1594	625
C088 ATQEVKNWMTDTLLV/C089 ASQEVKNWMTETLLV			13	300	0	163	38	575	0	0	0	Not done	394	0	0	269	63
C098 DCKTILRALGPGATL/C099 DCKTILKALGPAATL		P3	0	1150	0	0	163	8819	19	6	156	0	81	275	706	0	0
C100 ILRALGPGATLEEMM/C101 ILKALGPAATLEEMM			131	1700	0	0	25	2756	419	294	0	1400	281	375	444	175	0
C115 KNCRAPRKRGCWKCG/C116 RNCRAPRKKGCWKCG	Gag2		31	0	0	113	0	806	0	94	19	225	231	0	0	0	0
C127 NERQANFLGKIWPSH/C128 TERQANFLGKIWPSN			56	0	0	0	788	0	56	0	106	200	0	150	0	0	119
C129 ANFLGKIWPSHKGRP/C130 ANFLGKIWPSNKGRP			131	275	0	0	1025	3106	256	94	256	350	1106	125	0	113	644
C131 GKIWPSHKGRPGNFL/C132 GKIWPSNKGRPGNFP			0	0	25	0	288	2231	131	0	81	150	381	100	0	0	156
C135 GRPGNFLQSRPEPTA/C136 GRPGNFPQSRPEPSA			6	0	0	0	0	0	0	294	456	150	0	250	0	1013	6
C139 LPGKWKPKMIGGIGG/C140 LPGRWKPKMIGGIGG	Pol3	P4	0	288	0	344	81	0	6	0	13	0	388	0	106	381	0
C141 WKPKMIGGIGGFIKV			0	638	0	31	319	113	69	0	300	0	375	0	69	644	463
C142 MIGGIGGFIKVKQYD/C143 MIGGIGGFIKVRQYD			0	275	0	81	494	575	281	144	213	0	813	0	119	919	388
C146 IKVKQYDQILIEICG/C147 IKVRQYDQIPIEICG			0	400	169	231	231	1100	406	1669	900	750	713	231	94	769	300
C150 ILIEICGKKAIGTVL/C151 IPIEICGHKAIGTVL			0	1275	56	94	69	38	0	419	688	0	0	269	231	144	25
C152 ICGKKAIGTVLVGPT/C153 ICGHKAIGTVLIGPT			0	925	0	19	56	425	131	319	700	0	175	256	94	106	0
C154 KAIGTVLVGPTPVNI/C155 KAIGTVLIGPTPVNI			8	575	0	0	94	1425	119	6319	0	0	138	44	14431	519	0
C156 TVLVGPTPVNIIGRN/C157 TVLIGPTPVNIIGRN			0	200	44	0	381	850	94	5281	63	0	450	56	9744	156	363
C158 GPTPVNIIGRNMLTQ/C159 GPTPVNIIGRNLLTQ			0	0	106	0	294	0	831	169	75	0	425	156	331	356	163
C166 GCTLNFPISPIDTVP/C167 GCTLNFPISPIETVP			0	350	0	106	56	0	0	119	838	0	338	544	594	119	75
C168 NFPISPIDTVPVTLK/C169 NFPISPIETVPVKLK			3108	4475	6	156	81	900	194	344	888	100	175	0	806	6	213
C170 SPIDTVPVTLKPGMD/C171 SPIETVPVKLKPGMD			2458	4650	0	44	94	175	44	194	113	400	513	0	119	306	100
C174 TLKPGMDGPRVKQWP/C175 KLKPGMDGPKVKQWP			0	350	269	6	319	0	219	0	113	0	463	44	0	119	75
C176 GMDGPRVKQWPLTEE/C177 GMDGPKVKQWPLTEE			0	1350	144	494	81	325	194	119	313	0	363	194	19	181	0
C178 PRVKQWPLTEEKIKA/C179 PKVKQWPLTEEKIKA			0	775	19	281	0	0	119	69	0	1350	463	419	0	181	38
C194 GPENPYNTPIFAIKK/C195 GPENPYNTPVFAIKK		P5	81	0	0	0	0	231	0	Not done	844	0	50	0	169	94	0
C196 PYNTPIFAIKKKDST/C197 PYNTPVFAIKKKDST			556	25	19	0	1469	356	19	Not done	0	0	250	0	769	306	119
C198 PIFAIKKKDSTKWRK/C199 PVFAIKKKDSTRWRK			756	75	294	69	769	44	231	Not done	169	0	125	0	656	419	119
C202 DSTKWRKLVDFRELN/C203 DSTRWRKLVDFRELN			6	2588	0	6	231	531	44	Not done	0	0	0	0	0	156	69
C204 WRKLVDFRELNKKTQ/C205 WRKLVDFRELNKRTQ			131	50	281	0	544	956	206	Not done	0	0	0	194	456	931	0
C206 VDFRELNKKTQDFWE/C207 VDFRELNKRTQDFWE			431	75	206	0	644	881	169	Not done	0	163	0	294	331	1369	144
C218 GLKKKKSVTVLDVGD/C219 GLKKKRSVTVLDVGD			31	13275	0	0	31	1731	519	Not done	144	0	0	0	406	106	44
C220 KKSVTVLDVGDAYFS/C221 KRSVTVLDVGDAYFS		P6	13	194	75	431	1250	369	0	75	0	1725	294	0	0	0	3194
C222 TVLDVGDAYFSVPLD			38	56	0	781	738	894	0	63	206	600	69	0	231	119	1194
C224 VGDAYFSVPLDESFR/C225 VGDAYFSVPLDKDFR			138	1469	213	494	150	1181	56	663	106	0	319	0	269	81	944
C226 YFSVPLDESFRKYTA/C227 YFSVPLDKDFRKYTA			0	7644	2750	819	6788	269	0	400	219	2750	1456	0	219	369	8331
C228 PLDESFRKYTAFTIP/C229 PLDKDFRKYTAFTIP			100	10169	300	206	600	944	0	113	394	600	0	131	269	0	106
C230 SFRKYTAFTIPSINN/C231 DFRKYTAFTIPSTNN			238	1906	450	306	100	619	0	438	1994	0	0	0	194	0	6
C232 YTAFTIPSINNETPG/C233 YTAFTIPSTNNETPG			213	106	63	519	350	19	0	413	1456	0	0	31	119	31	169
C234 TIPSINNETPGIRYQ/C235 TIPSTNNETPGVRYQ			0	0	0	369	225	994	6	0	6	325	281	0	6	144	0
C236 INNETPGIRYQYNVL/C237 TNNETPGVRYQYNVL			163	81	0	856	488	244	31	163	19	275	44	0	394	0	0
C238 TPGIRYQYNVLPQGW/C239 TPGVRYQYNVLPMGW			100	306	363	606	825	419	706	1538	494	0	144	81	944	1131	0
C240 RYQYNVLPQGWKGSP/C241 RYQYNVLPMGWKGSP			0	2731	88	794	1513	144	506	1188	581	0	0	106	1006	1106	0
C242 NVLPQGWKGSPAIFQ/C243 NVLPMGWKGSPAIFQ			413	631	263	831	688	0	0	13	119	0	144	0	356	0	69
C244 QGWKGSPAIFQSSMT/C245 MGWKGSPAIFQCSMT			100	8306	163	294	288	594	0	0	56	0	106	0	269	381	0
C246 GSPAIFQSSMTKILE/C247 GSPAIFQCSMTKILE			150	756	88	381	150	194	0	13	56	0	0	0	256	0	56
C252 ILEPFRAKNPEIVIY/C253 ILEPFRKQNPDIVIY			50	606	0	381	150	94	0	113	9931	0	0	0	0	0	294
C254 FRAKNPEIVIYQYMD/C255 FRKQNPDIVIYQYMD			88	206	0	494	188	269	0	13	5269	375	0	2331	0	156	5419
C256 NPEIVIYQYMDDLYV/C257 NPDIVIYQYMDDLYI			263	1081	138	1619	1800	6269	181	913	8944	525	544	2906	2944	506	6281
C258 VIYQYMDDLYVGSDL/C259VIYQYMDDLYIGSDL			488	781	63	2019	1400	3844	56	1113	8369	0	1794	6	3156	1694	0
C266 IGQHRAKIEELREHL/C267IGQHRTKIEELRQHL		P7	0	338	0	0	0	925	0	0	69	0	150	0	256	0	88
C268 RAKIEELREHLLKWG/C269RTKIEELRQHLLRWG			38	988	0	75	1738	4600	0	0	456	0	113	0	644	0	0
C282 FLWMGYELHPDRWTV/C283 FHWMGYELHPDKWTV			0	0	0	250	125	700	138	0	2056	0	50	50	344	19	113
C292 PEKESWTVNDIQKLI/C293 PEKDSWTVNDIQKLV			138	150	0	250	1000	413	350	38	306	0	188	0	419	106	125
C294 SWTVNDIQKLIGKLN/C295 SWTVNDIQKLVGKLN			163	300	0	150	2638	4763	350	238	969	13	425	0	544	381	0
C296 NDIQKLIGKLNWASQ/C297 NDIQKLVGKLNWASQ			213	425	0	13	63	9188	0	13	844	0	0	0	969	0	100
C298 KLIGKLNWASQIY/C299 KLVGKLNWASQIY			38	0	0	513	225	0	25	13	6519	0	13	5050	19	0	4413
C302 KQGQDQWTYQIYQEP/C303 KQGQGQWTYQIYQEP	Pol4	P8	0	356	0	0	250	1519	131	0	81	0	138	50	0	31	0
C304 DQWTYQIYQEPFKNL/C305 GQWTYQIYQEPYKNL			175	356	81	106	1063	419	169	150	0	1219	25	0	2163	0	0
C306 YQIYQEPFKNLKTGK/C307 YQIYQEPYKNLKTGK			175	369	506	56	1225	931	194	1788	0	1719	263	325	4563	81	0
C310 FNLPPIVAKEIVANC/C311 FNLPPVVAKEIVASC	Pol5		0	1494	44	0	513	781	294	0	19	0	0	150	113	106	0
C312 PIVAKEIVANCDKCQ/C313 PVVAKEIVASCDKCQ			50	381	0	0	138	1406	119	0	19	0	238	88	125	144	6
C314 KEIVANCDKCQLKGE/C315 KEIVASCDKCQLKGE			88	0	56	0	163	831	81	0	0	0	213	0	0	6	0
C316 ANCDKCQLKGEAMHG/C317 ASCDKCQLKGEAIHG			0	631	169	56	63	744	44	125	0	0	50	0	0	219	0
C318 KCQLKGEAMHGQVDC/C319 KCQLKGEAIHGQVDC			100	256	0	131	100	394	19	0	169	0	188	0	125	0	0
C320 KGEAMHGQVDCSPGI/C321 KGEAIHGQVDCSPGI			38	0	0	106	175	481	31	100	0	0	100	0	75	0	0
C326 PGIWQLDCTHLEGKV/C327 PGMWQLDCTHLEGKV			0	231	0	0	63	781	106	0	0	0	0	0	0	0	0
C328 QLDCTHLEGKVILVA/C329 QLDCTHLEGKIILVA			125	256	0	0	100	2381	56	0	44	0	88	0	0	0	0
C332 GKVILVAVHVASGY/C333 GKIILVAVHVASGY			75	656	0	56	263	956	219	13	0	0	150	175	0	19	0
C335 IPYNPQSQGVVESMN	Pol6	P9	838	0	388	0	88	775	44	38	269	0	469	113	0	38	100
C344 IIGQVREQAEHLKTA/C345 IIGQVRDQAEHLKTA			138	0	300	19	163	525	56	1388	231	0	169	200	569	0	225
C346 VREQAEHLKTAVQMA/C347 VRDQAEHLKTAVQMA			100	0	0	0	25	175	19	0	231	0	331	125	444	138	50
C352 QMAVFIHNFKRKGGI/C353 QMAVLIHNFKRRGGI			113	333	1075	269	1675	50	2194	2738	0	200	344	750	2169	913	338
C354 FIHNFKRKGGIGGYS/C355 LIHNFKRRGGIGGYS			88	1508	375	94	1775	163	1744	1913	4781	0	681	1013	1544	463	113
C356 FKRKGGIGGYSAGER/C357 FKRRGGIGGYSAGER			113	1533	0	69	0	250	119	0	5131	0	506	213	0	0	263
C362 GERIIDIIATDIQTK/C363 GERIVDIIATDIQTR			338	133	88	456	0	1100	656	0	0	750	369	588	0	0	38
C364 IDIIATDIQTKELQK/C365 VDIIATDIQTRELQK			138	0	913	44	0	175	331	338	381	0	444	213	169	0	163
C366 ATDIQTKELQKQITK/C367 ATDIQTRELQKQIIK			0	1033	63	194	0	50	181	0	431	575	369	613	0	0	88
C368 QTKELQKQITKIQNF/C369 QTRELQKQIIKIQNF		P10	0	0	0	0	444	1425	0	38	119	0	0	0	0	463	81
C370 LQKQITKIQNFRVYY/C371 LQKQIIKIQNFRVYY			113	1031	0	31	656	3525	113	50	506	650	213	2863	356	300	1631
C372 ITKIQNFRVYYRDSR/C373 IIKIQNFRVYYRDSR			238	1669	13	431	956	4650	725	13	6906	1563	363	3688	694	50	1956
C374 QNFRVYYRDSRDPIW/C375 QNFRVYYRDSRDPLW			438	544	63	356	444	663	388	638	369	0	0	0	6	500	69
C376 VYYRDSRDPIWKGPA/C377 VYYRDSRDPLWKGPA			63	419	0	231	394	1025	63	113	419	0	0	0	156	75	119
C378 DSRDPIWKGPAKLLW/C379 DSRDPLWKGPAKLLW			88	19	0	231	731	0	63	0	319	0	0	0	0	0	56
C380 PIWKGPAKLLWKGEG/C381 PLWKGPAKLLWRGEG			100	644	0	219	519	2113	0	25	469	213	0	0	2581	0	0
C386 GEGAVVIQDNSDIKV/C387 GEGAVVIQDNSEIKV			25	131	0	94	381	1250	163	0	194	413	0	188	331	0	69
C388 VVIQDNSDIKVVPRR/C389 VVIQDNSEIKVVPRR			0	169	0	0	206	675	150	0	381	0	0	0	119	0	56
C390 DNSDIKVVPRRKVKI/C391 DNSEIKVVPRRKAKI			50	31	213	119	1406	125	913	0	344	213	88	0	69	750	0
C392 IKVVPRRKVKIIKDY/C393 IKVVPRRKAKIIRDY			50	4069	250	131	994	1200	1125	0	8631	0	213	0	56	650	81
C394 PRRKVKIIKDYGKQM/C395 PRRKAKIIRDYGKQM			100	11244	0	144	206	463	375	163	3481	663	0	0	256	0	0
C396 VKIIKDYGKQMAGAD/C397 AKIIRDYGKQMAGDD			50	7844	0	44	206	400	125	0	131	38	0	0	106	0	0

Figure S2D: **Volunteers' HLA class I and peptide pair mapping by STCL (CFHRZ)**

- 6000+
- 5000-5999
- 4000-4999
- 3000-3999
- 2000-2999
- 1000-1999
- 750-999

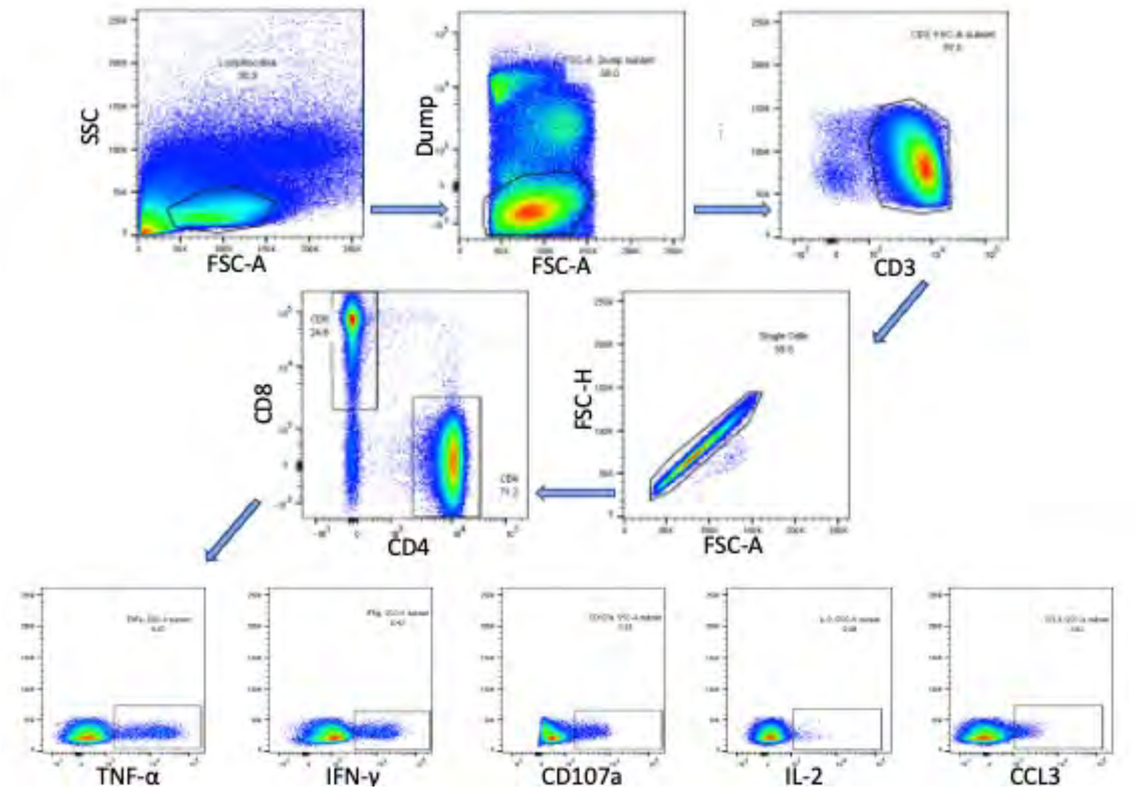

Figure S3: **Gating strategy for *ex vivo* functional analysis of vaccine-elicited T-cells**
Frozen PBMC of D35 were thawed and restimulated overnight with personalized peptide pools defined in figure S2.

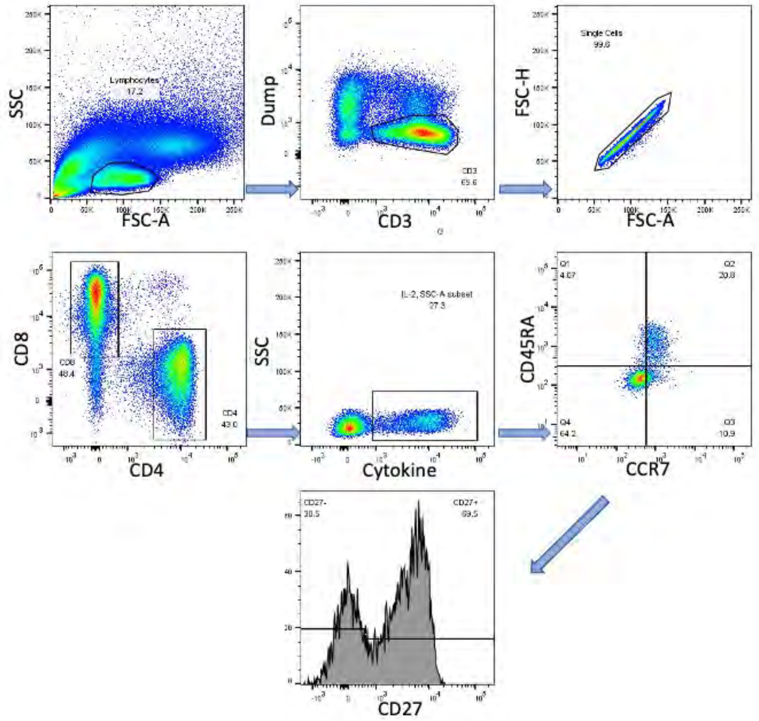

Figure S4: **Gating strategy for *ex vivo* memory structure of vaccine-elicited T-cells**
Frozen PBMC of D35 and personalized peptide pools defined in figure S2 were used for analysis.

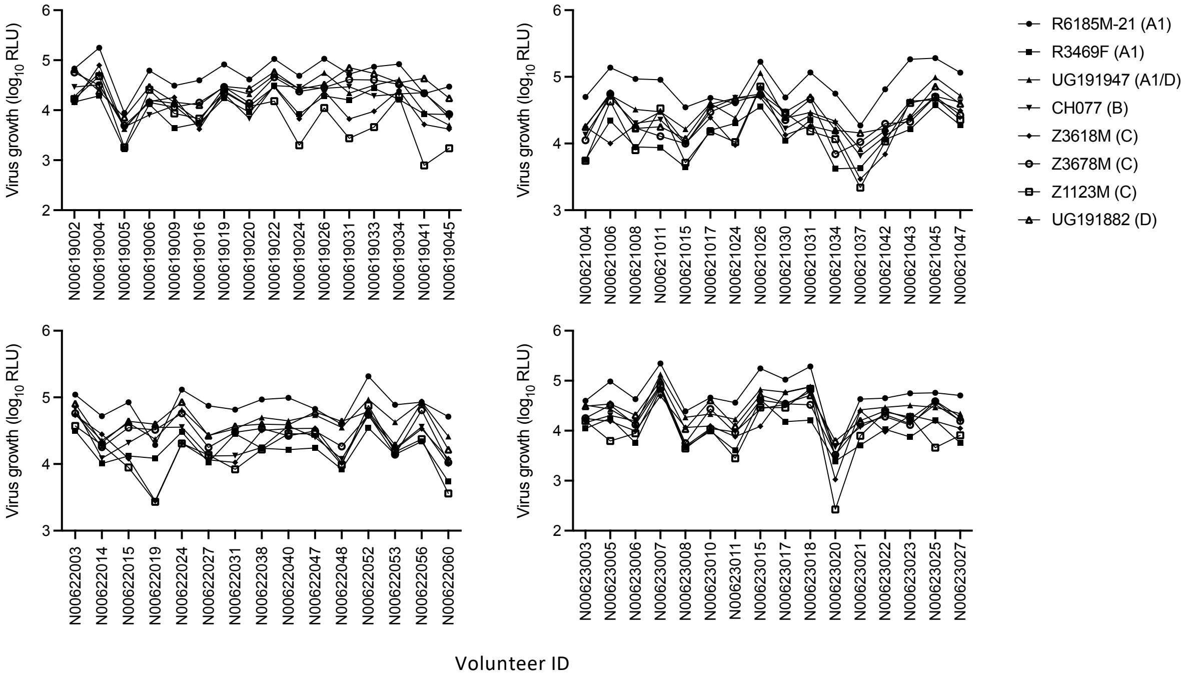

Figure S5: **Growth of IMCs of HIV-1 in volunteers' $CD4^+$ cells**
Thawed PBMC from D0 were expanded in the presence of bi-specific anti-CD3/CD8 mAbs for seven days to generate a $CD4^+$-enriched cell population, infected at MOI 0·01 with a panel of eight HIV-1 IMCs expressing the luciferase enzyme and cultured for a further five days. Virus growth was quantified by *LucR* enzyme in the infected cell lysates following the addition of an appropriate substrate. Luminescence is expressed in relative light units (RLU).

Region 1 – Gag1

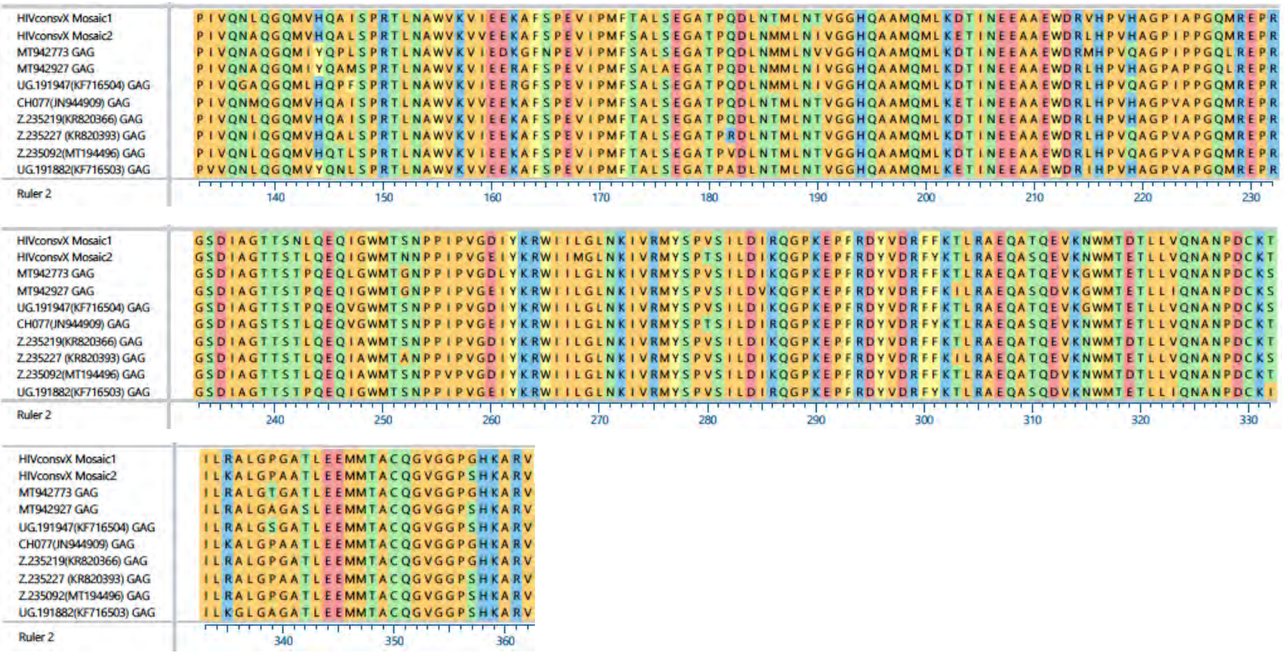

Region 2 – Gag2

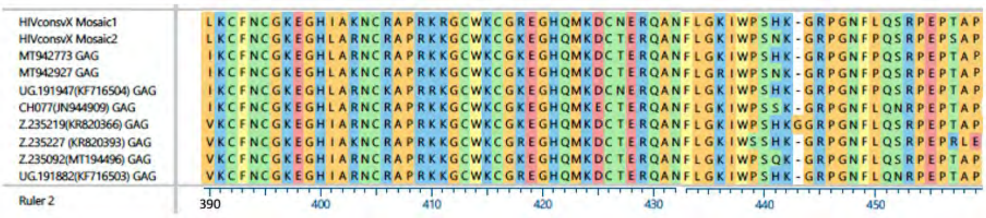

Region 3 – Pol1

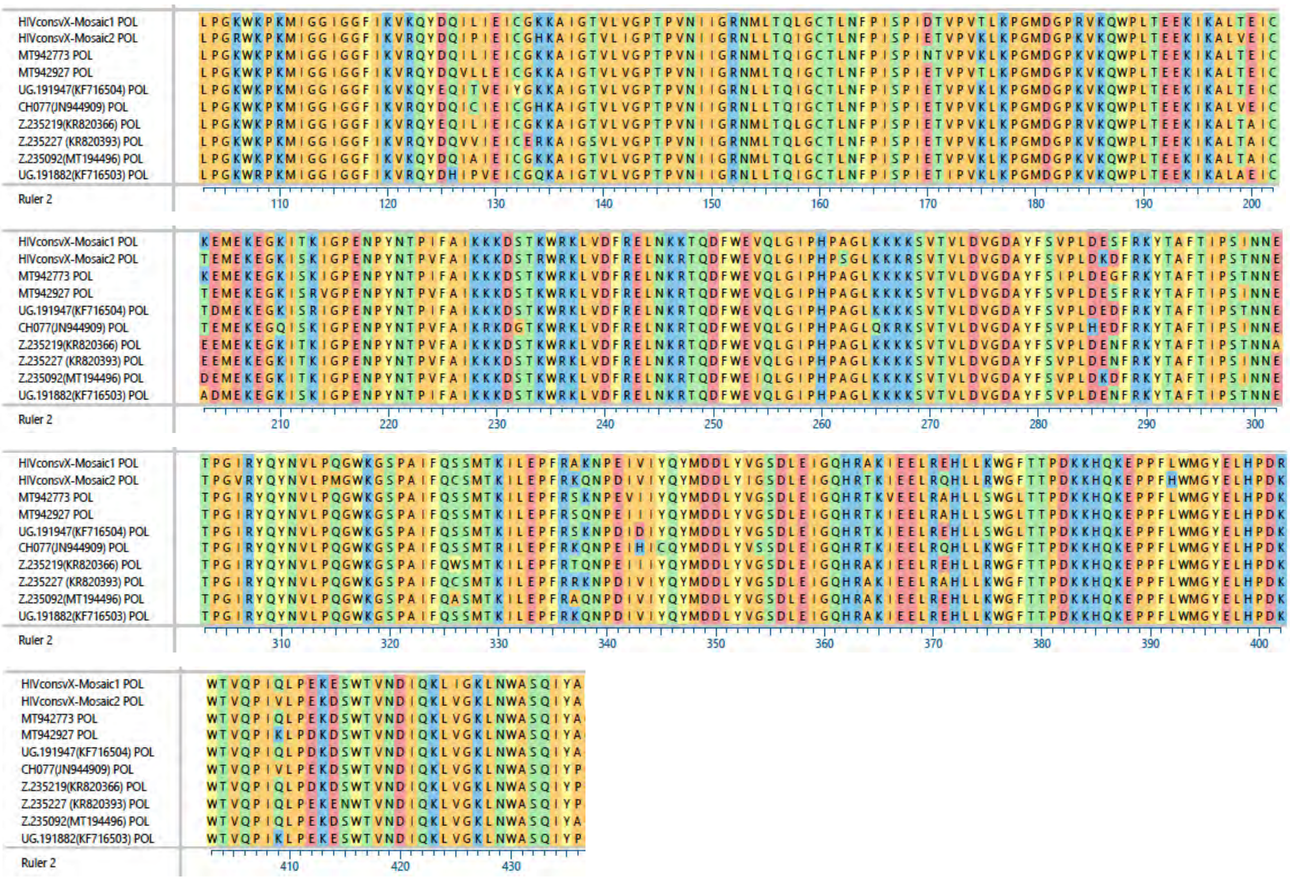

Figure S6: Alignment of HIVconsvX mosaics 1 and 2 with the VIA IMCs

Region 4 – Pol2

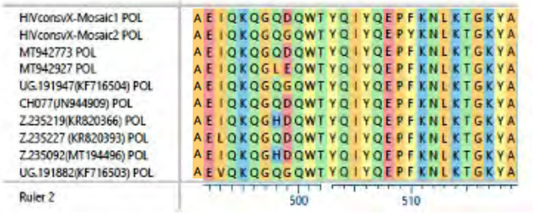

Region 5 – Pol3

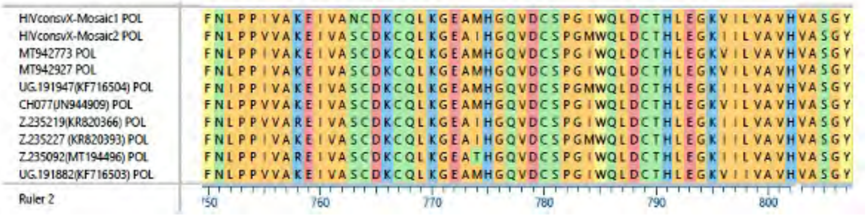

Region 6 – Pol4

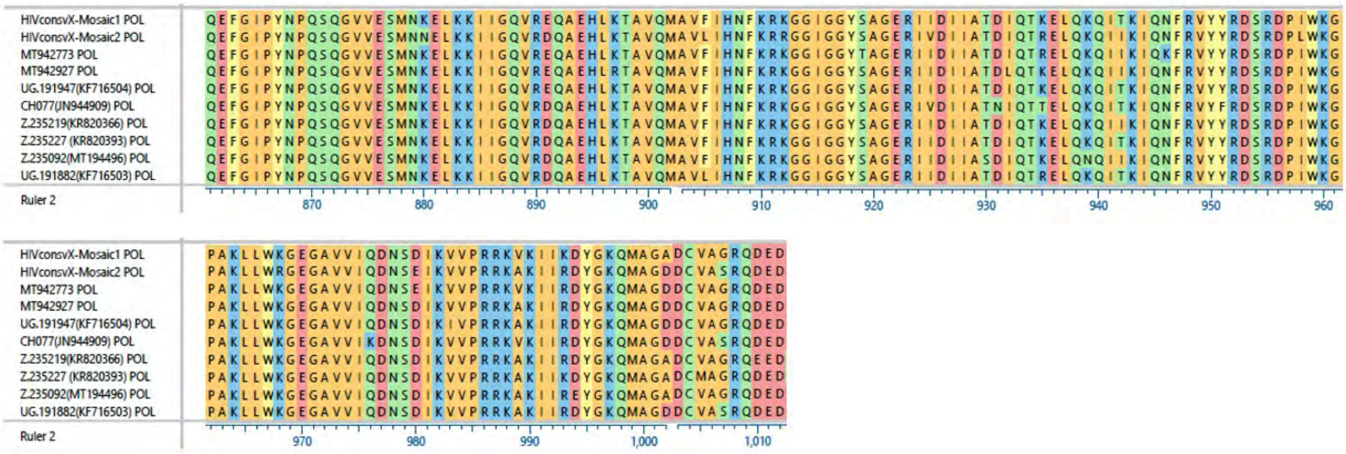

Figure S6: Alignment of HIVconsvX mosaics 1 and 2 with the VIA IMCs.

Continued. The amino acid residues were colour-coded into physico-chemical sub-groups: orange - hydrophobic non-polar with alkyl group; yellow - hydrophobic non-polar with aromatic group; green - hydrophilic polar neutral; red - hydrophilic polar acidic; and blue - hydrophilic polar basic.

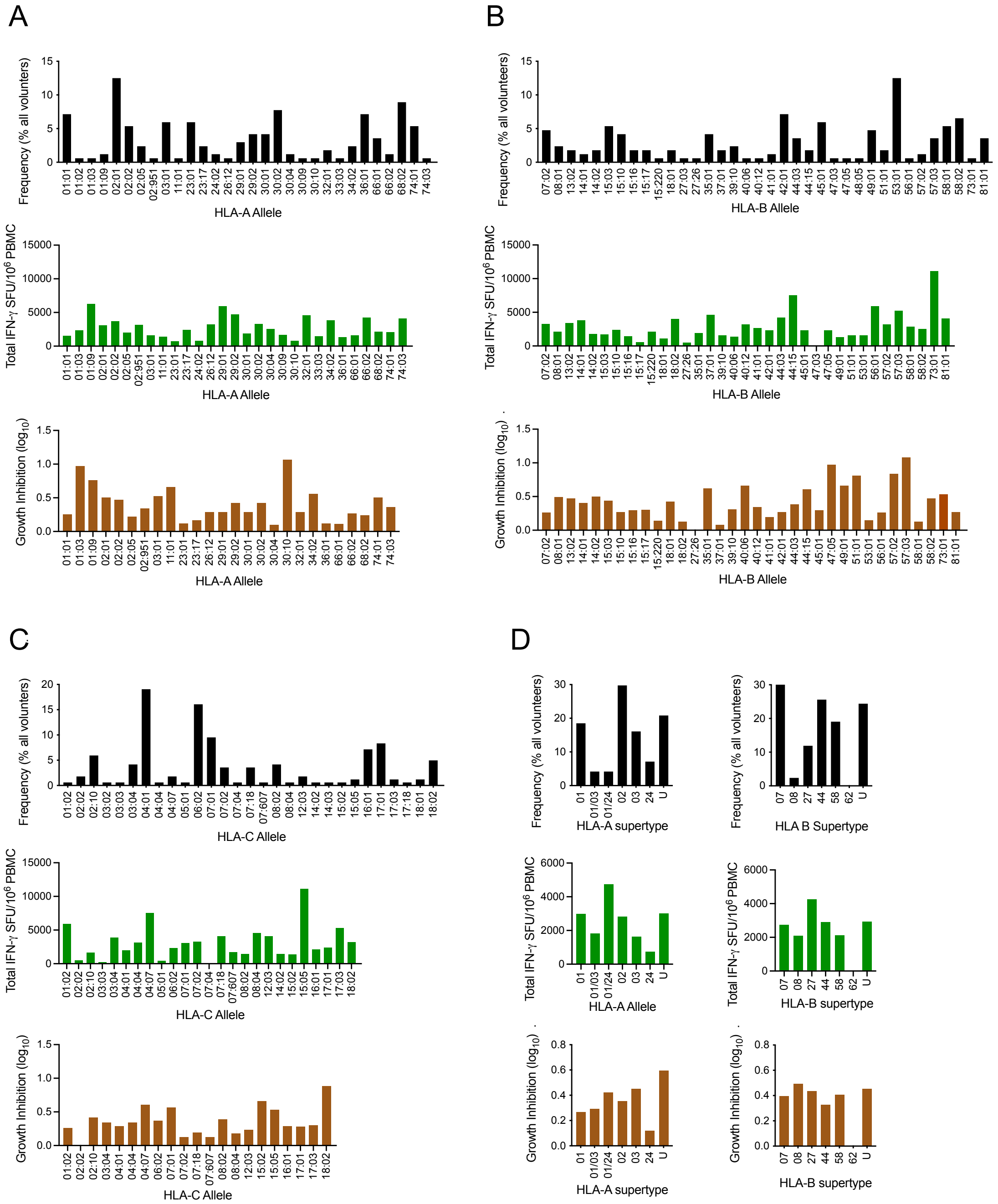

Figure S7: **HLA class I allele frequencies (black) and corresponding ELISPOT frequencies (green) and VIA (brown)**

HLA alleles were determined genetically using ssp PCR (IMGM Laboratories GmbH, Germany). HLA type (A-C) and supertype (D) frequencies for all study volunteers are shown, whereby U - unclassified supertype (black). Also shown are the frequencies of IFN-γ ELISPOT frequencies (green) and growth inhibition (brown) associated with each HLA allele. No comparisons achieved statistical significance.

Reason					
**Inclusion criteria (values included are for those who selected NO)**	**KAVI-ICR**	**MUL**	**KWTRP**	**CFHRZ**	**Overall**
Healthy male or female individual, as assessed by a medical history, physical exam, and laboratory tests?	**24 (39%)**	**13 (28%)**	**23 (45%)**	**3 (10%)**	**63 (33%)**
At low risk of HIV infection and willing to maintain low-risk behaviour for the duration of the trial. Individuals from key populations are not excluded provided they are assessed to be at low risk of HIV infection at screening and are willing to maintain low-risk behaviour during the study.	**2 (3%)**	**9 (19%)**	**7 (14%)**	0 (0%)	**18 (9%)**
At least 18 years of age on the day of screening and have not reached their 51st birthday on the day of the first vaccination?	0 (0%)	0 (0%)	0 (0%)	0 (0%)	0 (0%)
Willing and able to give informed consent for participation in the trial before any study-related procedures are performed. Volunteers will pass an Assessment of Understanding before signing the consent form.	0 (0%)	0 (0%)	0 (0%)	0 (0%)	0 (0%)
Willing to comply with the requirements of the protocol and be available for follow up for the planned duration of the study.	0 (0%)	0 (0%)	**1 (2%)**	0 (0%)	**1 (1%)**
Willing to undergo HIV testing, risk reduction counselling and receive HIV test results.	0 (0%)	0 (0%)	0 (0%)	0 (0%)	0 (0%)
All sexually active males (unless anatomically sterile or in a monogamous relationship with a female partner who uses a documented non-barrier method of birth control) must be willing to use an effective method of contraception (such as consistent condom use) from the day of first vaccination until 4 months after the last vaccination.	0 (0%)	**1 (2%)**	0 (0%)	0 (0%)	**1 (1%)**
If a female of childbearing potential, willing to use an effective non-barrier method of contraception (hormonal contraceptive or intrauterine device) from at least 2 weeks prior to first vaccination until at least 4 months after the last study vaccination. If not of childbearing potential: postmenopausal (>45 years of age with amenorrhea for at least 2 years, or any age with amenorrhea for at least 6 months and a serum follicle stimulating hormone [FSH] level>40 IU/L) or surgically sterile: no additional contraception required.	**1 (2%)**	0 (0%)	**1 (2%)**	0 (0%)	**2 (1%)**
All female volunteers must be willing to undergo urine pregnancy tests at time points indicated in the Schedule of Procedures and must test negative prior to each study vaccination.	**1 (2%)**	0 (0%)	0 (0%)	0 (0%)	**1 (1%)**
Willing to forego donations of blood or any other tissues during the trial and, for those who test positive for HIV antibodies due to vaccination (vaccine-induced seropositivity/ reactivity), until the anti-HIV antibody titres become undetectable.	0 (0%)	0 (0%)	0 (0%)	0 (0%)	0 (0%)

**Exclusion criteria (values included are for those who selected YES)**	**KAVI-ICR**	**MUL**	**KWTRP**	**CFHRZ**	**Overall**
Confirmed HIV-1 or HIV-2 infection	**1 (2%)**	**4 (9%)**	0 (0%)	0 (0%)	**5 (3%)**
Receipt of any vaccine in the previous 28 days or planned receipt within 28 days of Investigational Medicinal Product. Volunteers who expect to receive any adenoviral vectored vaccines within the next three months after ChAdOx1.tHIVconsv1 vaccine administration should not participate due to the risk of immune interference with the study vaccine.	**2 (3%)**	0 (0%)	0 (0%)	0 (0%)	**2 (1%)**
Participation in another clinical trial of an Investigational Medicinal Product currently, within the previous 3 months or expected participation during the study.	0 (0%)	0 (0%)	0 (0%)	0 (0%)	0 (0%)
Previous receipt of another investigational HIV vaccine candidate or any adenoviral vectored vaccine (Note: receipt of an HIV vaccine placebo will not exclude a volunteer from participation if documentation is available and the Medical Monitor gives approval).	0 (0%)	0 (0%)	0 (0%)	0 (0%)	0 (0%)
Receipt of blood transfusion or blood-derived products within the previous 4 months or expectation of receiving blood products during the study period.	0 (0%)	0 (0%)	0 (0%)	0 (0%)	0 (0%)
Receipt of immunoglobulin products within the previous 3 months.	0 (0%)	0 (0%)	0 (0%)	0 (0%)	0 (0%)
If female, pregnant or planning a pregnancy during the period of enrolment until 4 months after the last study vaccination; or lactating.	**1 (2%)**	0 (0%)	**1 (2%)**	0 (0%)	**2 (1%)**
Any confirmed or suspected history of immunodeficiency including recurrent severe infections.	0 (0%)	0 (0%)	0 (0%)	0 (0%)	0 (0%)
Use of systemic corticosteroids for >14 days (use of topical or inhaled steroids is permitted) within the previous 6 months.	0 (0%)	0 (0%)	0 (0%)	0 (0%)	0 (0%)
Immunosuppressive, anti-cancer, anti-tuberculosis or other medications considered significant by the investigator within the previous 6 months.	0 (0%)	0 (0%)	0 (0%)	0 (0%)	0 (0%)
History of splenectomy.	0 (0%)	0 (0%)	0 (0%)	0 (0%)	0 (0%)
History of autoimmune disease	0 (0%)	0 (0%)	0 (0%)	0 (0%)	0 (0%)
Immunosuppressive, anti-cancer, anti-tuberculosis or other medications considered significant by the investigator within the previous 6 months.	0 (0%)	0 (0%)	0 (0%)	0 (0%)	0 (0%)
History of splenectomy.	0 (0%)	0 (0%)	0 (0%)	0 (0%)	0 (0%)
History of autoimmune disease	0 (0%)	0 (0%)	0 (0%)	0 (0%)	0 (0%)
Bleeding disorder diagnosed by a physician (e.g., factor deficiency, coagulopathy or platelet disorder that requires special precautions). (Note: a volunteer that states that he or she has easy bruising or bleeding but does not have a formal diagnosis and has intramuscular injections and blood draws without any adverse experience is eligible).	0 (0%)	0 (0%)	0 (0%)	0 (0%)	0 (0%)

Table S1: **Reasons for screen failure (by CRCs)**

Reason	KAVI-ICR	MUL	KWTRP	CFHRZ	Overall
History of myocarditis, pericarditis, cardiomyopathy, congestive heart failure with permanent sequelae, or clinically significant arrhythmia (including any arrhythmia requiring medication, treatment, or clinical follow up).	0 (0%)	0 (0%)	0 (0%)	0 (0%)	0 (0%)
History of cancer, except basal cell carcinoma of the skin.	0 (0%)	0 (0%)	0 (0%)	0 (0%)	0 (0%)
Asthma that is not well controlled.	0 (0%)	0 (0%)	0 (0%)	0 (0%)	0 (0%)
History of allergic disease or reactions likely to be exacerbated by any component of the vaccine.	**1 (2%)**	0 (0%)	0 (0%)	0 (0%)	**1 (1%)**
History of severe local or systemic reactogenicity to vaccines (e.g., anaphylaxis, respiratory difficulty, angioedema).	0 (0%)	**1 (2%)**	0 (0%)	0 (0%)	**1 (1%)**
History of Guillain-Barre syndrome.	0 (0%)	0 (0%)	0 (0%)	0 (0%)	0 (0%)
Confirmed diagnosis of active or chronic hepatitis B, hepatitis C, active syphilis and/or active tuberculosis.	**1 (2%)**	**3 (6%)**	**2 (4%)**	**1 (3%)**	**7 (4%)**
Seizure disorder: an individual who has had a seizure in the last 3 years is excluded (Not excluded: an individual with a history of seizures who has neither required medication nor had a seizure for three or more years).	0 (0%)	0 (0%)	0 (0%)	0 (0%)	0 (0%)
History of serious psychiatric conditions or any psychiatric condition that precludes compliance with the protocol. Specifically excluded are persons with psychoses, ongoing risk for suicide, or history of suicide attempt or gesture within the past 3 years.	0 (0%)	0 (0%)	0 (0%)	0 (0%)	0 (0%)
Substance abuse disorder that precludes compliance with the protocol as assessed by the investigator.	**1 (2%)**	0 (0%)	**2 (4%)**	0 (0%)	**3 (2%)**
Any clinically significant acute or chronic medical condition that is considered unstable/progressive, or in the opinion of the Investigator, may either put the volunteer at risk because of participation in the study, or may influence the result of the study, or the volunteer's ability to participate in the study.	**1 (2%)**	0 (0%)	**12 (24%)**	0 (0%)	**13 (7%)**
Haematology 9.1 Haemoglobin - <9·5 g/dl in females; <11·0 g/dl in males	**1 (2%)**	0 (0%)	0 (0%)	0 (0%)	**1 (1%)**
Absolute Neutrophil Count (ANC) - ≤1,000/mm^3	0 (0%)	0 (0%)	**1 (2%)**	**1 (3%)**	**2 (1%)**
Absolute Lymphocyte Count (ALC) - ≤650/mm^3	0 (0%)	0 (0%)	0 (0%)	0 (0%)	0 (0%)
Platelets - < 100,000 cells/mm^3.	0 (0%)	0 (0%)	0 (0%)	0 (0%)	0 (0%)
Chemistry 9·4 Creatinine >1·1 x upper limit of normal (ULN).	**1 (2%)**	0 (0%)	**10 (20%)**	0 (0%)	**11 (6%)**
AST >1.25 x ULN	**1 (2%)**	0 (0%)	0 (0%)	0 (0%)	**1 (1%)**
ALT >1.25 x ULN	0 (0%)	0 (0%)	0 (0%)	**1 (3%)**	**1 (1%)**
Clinically significant abnormal dipstick confirmed by microscopy:9.7 Protein = 2+ or more.	**3 (5%)**	**1 (2%)**	**1 (2%)**	0 (0%)	**5 (3%)**
Blood = 2+ or more (not due to menses)	**2 (3%)**	0 (0%)	**4 (8%)**	0 (0%)	**6 (3%)**
Any other finding which in the opinion of the investigators would significantly increase the risk of having an adverse outcome from participating in the study.	**4 (6%)**	0 (0%)	**27 (53%)**	**1 (3%)**	**32 (17%)**
If, in the opinion of the Principal Investigator, it is not in the best interest of the volunteer to participate in the trial.	0 (0%)	**1 (2%)**	**27 (53%)**	0 (0%)	**28 (15%)**

Table S1: **Reasons for screen failure (by CRCs).**
Continued.

Site	Volunteers screened	Females screened	Males screened	Volunteers screened out	Volunteers enrolled	Females enrolled	Males enrolled
MUL	47 (100%)	18 (38%)	29 (62%)	25 (53%)	22 (47%)	5 (11%)	17 (36%)
KWTRP	50 (100%)	9 (18%)	41 (82%)	27 (54%)	23 (46%)	2 (4%)	21 (42%)
KAVI-ICR	62 (100%)	32 (52%)	30 (48%)	34 (55%)	22 (35%)	14 (23%)	8 (13%)
CFHRZ	27 (100%)	12 (44%)	15 (56%)	5 (19%)	22 (81%)	11 (41%)	11 (41%)
**Total**	**186 (100%)**	**71 (38%)**	**115 (62%)**	**91 (49%)**	**89 (48%)**	**32 (17%)**	**57 (31%)**

Table S2: **Recruitment summary by CRCs and overall**

Volunteer ID	AE description	Severity	Onset days after vaccination	Days between onset and resolution	Relation to IP	Outcome
N00619004	ABNORMAL VAGINAL DISCHARGE SYNDROME	Grade 2	26	12	No relationship	Resolved, no residual effects
N00619006	UPPER RESPIRATORY TRACT INFECTION	Grade 2	16	7	No relationship	Resolved, no residual effects
N00619006	MALARIA	Grade 2	14	9	No relationship	Resolved, no residual effects
N00619024	HEADACHE	Grade 2	24	10	No relationship	Resolved, no residual effects
N00619024	CHILLS	Grade 2	24	10	No relationship	Resolved, no residual effects
N00619041	SOFT TISSUE INJURY	Grade 2	26	11	No relationship	Resolved, no residual effects
N00619041	RIGHT ANKLE SPRAIN	Grade 2	26	8	No relationship	Resolved, no residual effects
N00619042	UNSPECIFIC FEBRILE ILLNESS	Grade 2	13	3	Unlikely	Resolved, no residual effects
N00619047	TONSILLITIS	Grade 2	20	2	No relationship	Resolved, no residual effects
N00621006	ARTHRITIS	Grade 1	8	11	No relationship	Resolved, no residual effects
N00621006	BILATERAL LOWER LIMB MUSCLE PAINS	Grade 1	15	18	No relationship	Resolved, no residual effects
N00622006	COUGH	Grade 1	5	7	No relationship	Resolved, no residual effects
N00622019	UPPER RESPIRATORY TRACT INFECTION	Grade 2	2	5	No relationship	Resolved, no residual effects
N00622027	VAGINAL CANDIDIASIS	Grade 1	26	5	No relationship	Resolved, no residual effects
N00622040	TOOTH ACHE	Grade 2	19	3	No relationship	Resolved, no residual effects
N00622044	FATIGUE	Grade 1	11	3	Possible (Yes expected)	Resolved, no residual effects
N00622044	HEADACHE	Grade 1	11	0	Possible (Yes expected)	Resolved, no residual effects
N00622048	CELLULITIS	Grade 2	10	11	No relationship	Resolved, no residual effects
N00623003	HEADACHE	Grade 2	18	1	No relationship	Resolved, no residual effects
N00623003	UPPER RESPIRATORY TRACT INFECTION	Grade 2	23	6	No relationship	Resolved, no residual effects
N00623005	ARTHRALGIA OF THE KNEES	Grade 2	12	2	No relationship	Resolved, no residual effects
N00623008	ACUTE ENTERITIS	Grade 2	2	3	Possible (Not expected)	Resolved, no residual effects
N00623016	FLU LIKE ILLNESS	Grade 3	26	8	Unlikely	Resolved, no residual effects
N00623017	BILATERAL EYE PAIN	Grade 1	1	1	Unlikely	Resolved, no residual effects
N00623021	PAIN AND DISCOLORATION AT VENEPUNCTURE SITE - RIGHT FOREARM	Grade 2	7	3	No relationship	Resolved, no residual effects
N00623021	UPPER RESPIRATORY TRACT INFECTION	Grade 1	20	6	No relationship	Resolved, no residual
N00623022	UPPER RESPIRATORY TRACT INFECTION	Grade 1	11	3	Unlikely	Resolved, no residual effects
N00623023	FLU LIKE ILLNESS	Grade 2	25	1	No relationship	Resolved, no residual effects
N00623024	HEADACHE	Grade 1	28	11	No relationship	Resolved, no residual effects

Highlighted in yellow are placebo recipients.

Table S3: **Unsolicited AEs reported after administration of the vaccines or placebo**

Volunteer ID	Days from vaccination	Category	Test	Result	Units	Severity
N00619019	19	Haematology	Platelet	$117x10^9$	cells/L	Grade 1
N00619019	28	Haematology	Platelet	$121x10^9$	cells/L	Grade 1
N00619019	47	Haematology	Platelet	$97x10^9$	cells/L	Grade 2
N00619019	50	Haematology	Platelet	$111x10^9$	cells/L	Grade 1
N00619019	132	Haematology	Platelet	$104x10^9$	cells/L	Grade 1
N00619024	37	Haematology	Platelet	$123x10^9$	cells/L	Grade 1
N00622040	27	Biochemistry	Bilirubin	59·1	mg/dL	Grade 1
N00623005	1	Haematology	Lymphocytes	$0{\cdot}35x10^9$	cells/L	Grade 3
N00623011	7	Haematology	White Blood Cell	$2{\cdot}36x10^9$	cells/L	Grade 1
N00623011	28	Haematology	White Blood Cell	$2{\cdot}41x10^9$	cells/L	Grade 1
N00623011	184	Haematology	White Blood Cell	$2{\cdot}27x10^9$	cells/L	Grade 1

Table S4: **Laboratory abnormalities**

No.	IMC-LucR.6ATRi	Clade	Country of isolation	Year of virus isolation	Type	GenBank
1	R.175019	A	Rwanda	2005	T/F	MT942773
2	R.175090	A	Rwanda	2010	T/F	MT942927
3	UG.191947	A/D	Uganda	2007	FSI	KF716504
4	CH077	B	USA	2006	T/F	JN944909
5	Z.235219	C	Zambia	2007	T/F	KR820366
6	Z.235227	C	Zambia	2009	T/F	KR820393
7	Z.235092	C	Zambia	2008	T/F	MT194496
8	UG.191882	D	Uganda	2009	FSI	KF716503

Table S5: **HIV-1 infectious molecular clones (IMCs) employed in VIA**
T/F – transmitted/founder virus; FSI – Fiebig stage I

Volunteer Magn/Pools	Visit day	Log₁₀ RLU reduction for CD4+/CD8+ T-cell co-cultures compared with CD4+ T cells alone								IMC inhibited per subject ≥0·1 log₁₀	
		A R.175019	A R.175090	A/D UG.191947	B CH077	C Z.235219	C Z.235227	C Z.235092	D UG.191882	D35	D308
**N00619002**	**D0**	0·92	0·59	0·93	0·94	0·56	0·79	0·71	0·85		
**7100/10**	**D35**	1·39	1·71	1·34	2·47	2·22	2·30	1·57	2·13	8	
	**D308**	1·23	1·55	1·32	2·14	1·72	1·84	1·42	1·77		8
**N00619004**	**D0**	0·15	-0·05	0·07	0·02	-0·01	-0·02	-0·10	-0·06		
**3100/9**	**D35**	0·50	0·86	1·22	0·59	0·38	0·54	0·30	0·91	8	
	**D308**	0·38	0·30	0·41	0·29	0·06	0·21	0·05	0·40		8
**N00619005**	**D0**	-0·10	-0·10	0·09	0·24	-0·10	0·17	-0·22	0·35		
**3960/9**	**D35**	0·58	1·01	0·60	0·98	0·00	1·30	0·53	1·23	8	
	**D308**	0·47	0·72	0·46	0·67	-0·05	0·81	-0·02	0·99		7
**N00619006 P**	**D0**	0·47	0·56	0·51	0·53	0·26	0·61	0·43	0·85		
**70/0**	**D35**	0·42	0·40	0·40	0·36	0·17	0·49	0·33	0·77	0	
	**D308**	0·44	0·51	0·48	0·32	0·23	0·52	0·38	0·84		0
**N00619009**	**D0**	0·14	0·16	0·20	0·27	0·12	0·34	0·07	0·34		
**3480/9**	**D35**	0·30	0·40	0·88	0·45	0·14	0·74	0·09	1·21	6	
	**D308**	0·32	0·34	0·62	0·34	0·18	0·48	0·19	0·75		6
**N00619016**	**D0**	0·64	0·52	0·34	0·39	0·26	0·60	0·30	0·46		
**10090/9**	**D35**	1·05	1·12	0·83	0·91	0·59	1·06	0·94	1·03	8	
	**D308**	0·94	0·87	0·83	0·77	0·52	0·97	0·83	0·96		8
**N00619019**	**D0**	0·80	0·85	0·76	0·90	0·48	0·84	0·80	0·78		
**7550/10**	**D35**	1·58	1·02	1·61	1·09	1·09	1·33	0·93	1·20	8	
	**D308**	0·99	0·94	1·14	0·94	0·58	1·02	0·88	0·97		5
**N00619020**	**D0**	0·34	0·32	0·47	0·36	0·35	0·38	0·36	0·48		
**4730/9**	**D35**	0·71	0·95	1·11	0·70	0·54	0·78	0·62	1·02	8	
	**D308**	0·63	0·60	0·70	0·55	0·37	0·48	0·39	0·79		6
**N00619022**	**D0**	0·30	0·54	0·39	0·43	0·33	0·39	0·44	0·55		
**5930/10**	**D35**	0·55	0·70	0·70	0·56	0·71	0·69	0·66	0·82	8	
	**D308**	0·28	0·55	0·38	0·48	0·40	0·43	0·36	0·58		0
**N00619024**	**D0**	0·24	0·06	0·24	0·36	0·09	0·20	0·15	0·29		
**6200/10**	**D35**	0·56	0·52	0·78	0·67	0·23	0·51	0·33	0·73	8	
	**D308**	0·19	0·12	0·17	0·37	-0·02	0·11	-0·04	0·29		0
**N00619026**	**D0**	0·48	0·52	0·58	0·70	0·39	0·59	0·59	0·62		
**4230//10**	**D35**	0·94	0·76	1·11	0·99	0·64	0·71	0·95	0·86	8	
	**D308**	0·83	0·77	1·01	0·71	0·36	0·57	0·62	0·56		3
**N00619031**	**D0**	0·70	0·54	0·52	0·61	0·46	0·71	0·68	0·83		
**6240/10**	**D35**	0·88	0·91	1·11	0·88	1·09	1·15	0·81	1·41	8	
	**D308**	0·76	0·68	0·80	0·71	0·58	0·90	0·76	1·03		6
**N00619033**	**D0**	0·49	0·56	0·53	0·55	0·38	0·62	0·48	0·62		
**6090/9**	**D35**	2·36	0·82	1·90	0·76	0·37	1·28	0·80	1·45	7	
	**D308**	1·84	0·48	1·46	0·44	0·21	0·82	0·43	1·01		4
**N00619034**	**D0**	0·28	0·33	0·27	0·29	0·10	0·33	0·23	0·39		
**4480/6**	**D35**	0·44	1·13	0·85	1·52	1·59	1·48	0·63	1·46	8	
	**D308**	0·29	0·51	0·48	0·58	0·61	0·67	0·35	0·82		7
**N00619041**	**D0**	0·43	0·43	0·36	0·56	0·41	0·63	ivg	0·54		
**4090/10**	**D35**	1·15	0·42	1·00	1·06	0·67	0·56	ivg	0·90	5	
	**D308**	0·91	0·49	0·77	0·77	0·42	0·55	ivg	0·69		4

The 1st column shows volunteer ID (placebos highlighted yellow) and ELISPOT data (Magn/Pools - total magnitude as SFU/10^6 PBMC/Number of recognized pools). P – placebo. Dark red – inhibition relative to day 0 increased by more than ≥1·0 log₁₀; medium red - inhibition relative to day 0 increased by <1·0 log₁₀ to ≥0·1 log₁₀; light red - inhibition relative to day 0 increased by <0·1 log₁₀; no colour – no increase in inhibition relative to pre-vaccination· nd – no data; ivg – insufficient virus growth.

Table S6A: **Cross-clade HIV inhibition *in vitro* (MUL)**

Volunteer Magn/Pools	Visit day	Log10 RLU reduction for CD4+/CD8+ T-cell co-cultures compared with CD4+ T cells alone								IMC inhibited per subject ≥0·1 log10	
		A R.175019	A R.175090	A/D UG.191947	B CH077	C Z.235219	C Z.235227	C Z.235092	D UG.191882	D35	D308
N00621004	D0	0·60	0·45	0·45	0·63	-0·01	0·43	0·01	0·46		
11130/10	D35	0·67	1·30	0·76	1·50	0·65	0·86	0·61	0·93	7	
	D308	0·76	1·19	0·74	1·19	0·54	0·87	0·62	0·88		8
N00621006	D0	0·16	0·19	0·23	0·31	0·11	0·25	0·47	0·37		
1370/7	D35	0·83	0·91	1·18	0·52	0·30	0·68	1·11	1·46	8	
	D308	0·26	0·31	0·37	0·34	0·19	0·28	0·61	0·43		5
N00621008	D0	0·37	0·21	0·64	0·67	0·50	0·40	0·32	0·36		
14470/10	D35	0·48	0·25	0·80	0·74	0·73	0·71	0·19	0·47	6	
	D308	0·37	0·27	0·58	0·63	0·55	0·59	0·09	0·33		1
N00621011	D0	0·49	0·42	0·57	0·84	0·36	0·59	0·36	0·86		
4590/5	D35	0·59	0·60	1·11	0·91	0·54	0·89	0·52	1·33	7	
	D308	0·56	0·52	0·78	0·74	0·41	0·70	0·31	1·05		5
N00621015	D0	0·22	0·22	0·29	0·49	0·08	0·51	0·07	0·37		
5240/10	D35	0·61	0·53	0·87	0·86	0·41	0·58	0·53	1·01	7	
	D308	0·42	0·42	0·60	0·74	0·33	0·56	0·33	0·74		7
N00621017	D0	0·70	0·77	0·67	0·87	0·62	0·79	0·86	0·78		
2520/8	D35	0·80	0·85	0·76	0·74	0·84	0·82	1·05	0·97	4	
	D308	0·78	0·76	0·68	0·72	0·85	0·75	0·96	0·69		2
N00621024	D0	0·70	0·83	0·64	1·09	0·57	0·79	0·73	0·84		
3980/10	D35	0·83	0·90	0·85	1·36	0·68	0·92	0·80	1·00	5	
	D308	0·77	0·93	0·70	1·14	0·66	0·88	0·83	0·93		2
N00621026	D0	0·58	0·89	0·75	0·72	0·67	0·80	0·85	0·76		
6820/7	D35	0·94	1·25	1·02	0·95	0·80	1·31	1·03	1·13	8	
	D308	0·66	0·84	0·74	0·61	0·66	0·73	0·92	0·67		0
N00621030	D0	0·15	0·23	0·40	0·47	0·22	0·44	0·64	0·49		
16740/10	D35	0·34	1·03	0·90	0·83	1·43	1·26	0·74	1·17	8	
	D308	0·38	1·02	1·07	1·04	1·53	1·34	0·91	1·46		8
N00621031	D0	0·30	0·30	0·23	0·31	0·18	0·25	0·43	0·38		
11190/10	D35	2·07	0·70	1·68	0·63	1·30	1·00	0·76	1·15	8	
	D308	0·89	0·54	0·85	0·35	0·73	0·55	0·59	0·67		7
N00621034	D0	0·30	0·21	0·42	0·53	0·33	0·13	0·32	0·45		
4080/6	D35	0·45	0·41	0·59	0·54	0·39	0·47	0·41	0·54	4	
	D308	0·41	0·32	0·51	0·55	0·44	0·35	0·47	0·60		7
N00621037	D0	0·32	0·24	0·23	0·28	0·23	0·48	0·44	0·54		
8050/5	D35	0·84	1·46	0·61	1·43	1·41	1·74	1·12	1·57	8	
	D308	0·52	0·84	0·36	1·17	1·02	1·24	0·72	1·09		8
N00621042 P	D0	0·62	0·58	0·42	0·56	0·37	0·55	0·41	0·42		
40/0	D35	0·11	0·19	0·15	0·18	0·08	0·13	0·10	0·17	0	
	D308	0·67	0·59	0·51	0·52	0·25	0·46	0·31	0·30		0
N00621043	D0	0·61	0·53	0·52	0·65	0·19	0·60	0·52	0·42		
2110/9	D35	0·70	0·60	0·60	0·76	0·24	0·71	0·63	0·56	3	
	D308	0·89	0·68	0·78	0·83	0·41	0·85	0·80	0·62		8
N00621045	D0	0·82	0·81	0·94	1·03	0·59	0·82	0·74	0·85		
2310/8	D35	0·67	0·91	0·85	0·82	0·41	1·16	0·90	1·11	4	
	D308	0·78	0·87	0·91	0·93	0·50	0·80	0·68	0·74		0
N00621047	D0	0·78	0·52	0·70	0·77	0·53	0·52	0·68	0·58		
3390/10	D35	1·19	0·92	1·19	1·28	1·00	0·92	1·17	1·05	8	
	D308	1·26	0·89	1·10	1·05	0·96	1·02	1·18	1·00		8

Table S6B: **Cross-clade HIV inhibition** ***in vitro*** **(KWTRP)**

Volunteer	Visit	Log_{10} RLU reduction for $CD4^+/CD8^+$ T-cell co-cultures compared with $CD4^+$ T cells alone								IMC inhibited per subject	
		A	A	A/D	B	C	C	C	D	≥0·1 log_{10}	
Magn/Pools	day	R.175019	R.175090	UG.191947	CH077	Z.235219	Z.235227	Z.235092	UG.191882	D35	D308
**N00622003**	**D0**	0·46	0·70	0·53	0·68	0·53	0·64	0·55	0·72		
**490/4**	**D35**	0·44	0·78	0·62	0·69	0·42	0·63	0·48	0·79	0	
	**D308**	0·54	0·80	0·58	0·73	0·39	0·65	0·54	0·77		1
**N00622006**	**D0**	0·94	0·81	0·88	0·85	0·72	0·81	0·87	0·71		
**3170/10**	**D35**	1·17	1·16	1·22	1·14	1·14	1·08	1·37	1·09	8	
	**D308**	1·18	1·03	0·90	1·04	0·83	0·96	1·20	0·90		7
**N00622014**	**D0**	0·52	0·74	0·57	0·74	0·52	0·73	0·58	0·75		
**4060/9**	**D35**	0·60	1·06	0·84	0·77	0·58	0·71	0·78	0·96	4	
	**D308**	0·65	0·94	0·69	0·88	0·59	0·81	0·74	0·90		6
**N00622015 P**	**D0**	1·08	0·91	0·95	1·01	0·52	1·04	0·70	1·04		
**20/0**	**D35**	0·84	0·70	0·72	0·82	0·35	0·77	0·47	0·83	0	
	**D308**	0·90	0·70	0·74	0·83	0·42	0·85	0·50	0·83		0
**N00622019**	**D0**	0·51	0·67	0·68	0·66	0·35	0·69	0·57	0·69		
**1020/9**	**D35**	0·71	0·64	0·79	0·64	0·37	0·83	0·46	0·69	3	
	**D308**	0·37	0·53	0·43	0·52	0·11	0·49	0·31	0·45		0
**N00622024**	**D0**	0·66	0·71	0·57	0·73	0·43	1·22	0·56	1·03		
**1590/8**	**D35**	0·83	0·95	0·92	0·98	0·32	0·75	0·54	0·82	4	
	**D308**	0·78	0·85	0·70	0·82	0·47	0·90	0·60	0·94		3
**N00622027**	**D0**	0·94	0·83	0·84	1·04	0·55	0·90	0·68	0·86		
**1715/7**	**D35**	1·05	0·90	0·97	1·08	0·69	1·02	0·83	1·00	5	
	**D308**	1·10	0·95	1·01	1·08	0·70	0·95	0·80	0·95		6
**N00622031**	**D0**	0·48	0·64	0·50	0·56	0·25	0·62	0·24	0·56		
**530/1**	**D35**	1·59	0·75	1·68	0·64	0·27	0·65	0·23	1·05	4	
	**D308**	0·65	0·66	0·60	0·53	0·23	0·54	0·25	0·58		2
**N00622038**	**D0**	0·42	0·49	0·59	0·62	0·43	0·75	0·26	0·62		
**4430/10**	**D35**	1·08	1·82	1·35	2·02	2·40	2·49	1·52	2·15	8	
	**D308**	0·76	1·24	0·93	1·38	1·47	1·60	0·73	1·52		8
**N00622040**	**D0**	0·69	0·42	0·57	0·45	0·33	0·58	0·54	0·54		
**800/4**	**D35**	0·78	1·43	0·87	1·95	1·79	1·84	1·34	1·66	7	
	**D308**	0·78	0·85	0·85	1·23	1·01	1·27	0·83	1·01		7
**N00622047**	**D0**	0·54	0·68	0·50	0·64	0·19	0·64	0·32	0·65		
**730/6**	**D35**	0·71	0·77	0·55	0·64	0·34	1·15	0·57	0·68	4	
	**D308**	nd	nd	nd	nd	nd	nd	nd	nd		
**N00622048**	**D0**	0·50	0·60	0·75	0·70	0·37	0·79	0·73	0·99		
**2380/8**	**D35**	0·75	0·89	1·41	0·73	0·53	1·28	0·82	1·71	6	
	**D308**	0·50	0·62	0·89	0·60	0·38	0·88	0·66	1·20		3
**N00622052**	**D0**	0·52	0·46	0·68	0·61	0·53	0·67	0·60	0·62		
**2330/10**	**D35**	1·33	1·42	1·69	1·63	1·42	1·52	1·57	1·72	8	
	**D308**	1·08	1·14	1·35	1·40	1·22	1·18	1·37	1·45		8
**N00622053**	**D0**	0·84	0·66	0·80	0·79	0·34	0·73	0·65	0·70		
**2510/10**	**D35**	1·64	1·52	1·78	1·32	0·88	1·35	1·11	1·44	8	
	**D308**	1·18	0·95	1·24	1·11	0·55	0·97	0·87	1·04		8
**N00622056**	**D0**	0·48	0·68	0·78	0·70	0·46	0·92	0·62	0·80		
**740/4**	**D35**	0·67	1·00	0·98	0·93	0·58	1·25	0·68	1·12	7	
	**D308**	0·60	0·85	0·91	0·89	0·59	1·15	0·72	1·00		8
**N00622060**	**D0**	0·36	0·29	0·46	0·42	0·27	0·27	0·10	0·39		
**6940/10**	**D35**	0·51	0·43	0·65	0·42	0·23	0·40	0·70	1·04	6	
	**D308**	0·55	0·44	0·64	0·50	0·33	0·41	0·43	0·69		6

Table S6C: **Cross-clade HIV inhibition *in vitro* (KAVI-ICR)**

Volunteer Magn/Pools	Visit day	Log$_{10}$ RLU reduction for CD4$^+$/CD8$^+$ T-cell co-cultures compared with CD4$^+$ T cells alone								IMC inhibited per subject ≥0·1 log$_{10}$	
		A R.175019	A R.175090	A/D UG.191947	B CH077	C Z.235219	C Z.235227	C Z.235092	D UG.191882	D 35	D308
**N00623003**	**D0**	0·14	0·16	0·17	0·38	-0·10	0·21	0·04	0·62		
**1550/5**	**D35**	0·27	0·24	0·35	0·42	0·04	0·42	0·10	0·56	5	
	**D308**	0·15	0·17	0·29	0·42	-0·02	0·43	0·07	0·60		2
**N00623006**	**D0**	0·21	0·16	0·23	0·40	-0·05	0·30	0·13	0·40		
**610/3**	**D35**	0·14	0·15	0·25	0·33	0·18	0·30	0·15	0·38	1	
	**D308**	0·36	0·28	0·24	0·45	0·04	0·34	0·20	0·50		3
**N00623008**	**D0**	-0·06	0·04	0·10	0·14	-0·01	0·01	0·10	0·21		
**655/4**	**D35**	0·17	0·92	0·74	1·12	0·93	0·79	0·69	1·17	8	
	**D308**	-0·02	0·15	0·35	0·42	0·14	0·25	0·22	0·49		7
**N00623010**	**D0**	0·35	0·24	0·35	0·38	0·25	0·48	0·40	0·47		
**1910/6**	**D35**	0·81	1·04	0·73	1·30	1·24	1·37	0·59	1·14	8	
	**D308**	0·53	0·44	0·44	0·72	0·65	0·69	0·47	0·64		7
**N00623011**	**D0**	0·75	0·24	0·56	0·38	0·18	0·39	0·06	0·48		
**2590/8**	**D35**	1·02	0·48	0·96	0·67	0·52	0·81	0·23	0·77	8	
	**D308**	0·94	0·46	0·85	0·56	0·23	0·54	0·11	0·57		5
**N00623015**	**D0**	0·81	0·64	0·70	0·80	0·32	0·65	0·80	0·55		
**1000/8**	**D35**	0·87	0·74	0·84	0·89	0·48	0·81	0·87	0·60	4	
	**D308**	0·78	0·58	0·70	0·70	0·35	0·65	0·75	0·44		0
**N00623017**	**D0**	0·27	0·23	0·36	0·35	0·43	0·47	0·61	0·43		
**1315/5**	**D35**	0·71	0·76	0·71	0·72	0·47	0·74	0·68	0·70	6	
	**D308**	0·36	0·35	0·39	0·50	0·32	0·46	0·49	0·62		3
**N00623018**	**D0**	0·37	0·21	0·34	0·52	0·42	0·27	0·45	0·40		
**1560/10**	**D35**	1·07	1·20	0·99	1·43	0·84	1·45	1·49	1·23	8	
	**D308**	0·66	0·84	0·79	0·94	0·57	0·98	1·23	0·97		8
**N00623020**	**D0**	0·16	0·33	0·24	0·32	0·01	0·32	ivg	0·45		
**500/2**	**D35**	1·01	0·54	1·00	0·56	0·26	1·22	ivg	1·07	7	
	**D308**	0·38	0·42	0·47	0·47	0·19	0·57	ivg	0·61		6
**N00623021**	**D0**	0·06	0·01	0·07	-0·02	0·10	0·03	0·02	0·01		
**2070/9**	**D35**	0·57	0·13	0·87	0·48	0·21	0·79	0·12	0·62	8	
	**D308**	nd	nd	nd	nd	nd	nd	nd	nd		
**N00623022**	**D0**	0·32	0·55	0·51	0·65	0·15	0·53	0·49	0·58		
**1080/4**	**D35**	0·54	0·98	1·24	0·84	0·23	1·05	0·90	1·53	7	
	**D308**	0·34	0·57	0·65	0·60	0·12	0·56	0·49	0·68		2
**N00623025**	**D0**	0·72	0·60	0·66	0·93	0·86	0·82	0·57	0·72		
**750/5**	**D35**	0·79	0·86	0·69	0·79	0·78	0·73	0·48	0·76	1	
	**D308**	0·63	0·59	0·59	0·73	0·70	0·70	0·49	0·56		0
**N00623027**	**D0**	0·22	0·25	0·33	0·37	0·24	0·34	0·30	0·41		
**3115/4**	**D35**	0·44	0·52	0·80	0·48	0·48	1·63	1·54	1·07	8	
	**D308**	0·41	0·43	0·54	0·47	0·15	0·54	0·69	0·78		7

Table S7D: **Cross-clade HIV inhibition *in vitro* (CFHRZ)**

Trial Title: A Phase 1 Trial of ChAdOx1- and MVA-vectored Conserved Mosaic HIV-1 Vaccines in Healthy, Adult HIV-1-negative Volunteers in Eastern and Southern Africa.

Internal Reference Number / Short title: HIV-CORE 006

Ethics Ref: OXTREC Ref: 56-19

Date and Version No: version 4.0, 01 December 2021

Chief Investigator: Dr Paola Cicconi, MD, PhD
The Jenner Institute
University of Oxford
Centre for Clinical Vaccinology and Tropical Medicine (CCVTM)
Churchill Hospital, Old Road
Oxford OX3 7LE
United Kingdom
Phone: +44 (0)1865 611413
Email: paola.cicconi@ndm.ox.ac.uk

Chief Non-Clinical Investigator: Professor Tomáš Hanke, BSc, MSc, PhD
The Jenner Institute
University of Oxford
Old Road Campus Research Building
Roosevelt Drive
Oxford OX3 7DQ
United Kingdom
Phone: +44 (0)1865 617630
Email: tomas.hanke@ndm.ox.ac.uk

Principal Investigators: Professor Walter Jaoko, MBChB, MTropMed, PhD,
Department of Medical Microbiology
University of Nairobi
Second Floor, PO Box 19676-00202
Off Ngong Road, Nairobi
Kenya
Phone: +254-2-2717694 / +254 727555254
Email: wjaoko@kaviuon.org

William Kilembe, MBChB, MSc,
Center for Family Health Research in Zambia
B22/F737 Mwembelelo Road
Emmasdale, Lusaka
Zambia
Phone: +260 966862787/ +260 966851820
Email: wkilembe@rzhrg-mail.org

Prof. Eduard Sanders, MD, MPH, PhD
KEMRI/ Wellcome Trust Research Programme
Hospital Road
P.O. Box 230-80108 Kilifi,
Kenya.
Phone: +254 723593762
Email: ESanders@kemri-wellcome.org

Pontiano Kaleebu, MBChB, PhD
MRC/UVRI & LSHTM, Uganda Research Unit
P.O.BOX 49 Entebbe,
Uganda.
Tel: +256 417 704 103
E-mail: Pontiano.Kaleebu@mrcuganda.org

Sponsor: University of Oxford, United Kingdom

Funder: European and Developing Countries Clinical Trials Partnership

TABLE OF CONTENTS

1 KEY TRIAL CONTACTS ... 7
2 SYNOPSIS ... 11
ABBREVIATIONS ... 14
3 BACKGROUND AND RATIONALE ... 15
4 OBJECTIVES AND OUTCOME MEASURES ... 21
5 TRIAL DESIGN ... 22
6 VOLUNTEER IDENTIFICATION ... 23
6.1 Study Population ... 23
6.2 Inclusion Criteria ... 23
6.3 Exclusion Criteria ... 23
6.4 Recruitment of Volunteers ... 25
7 TRIAL VISITS ... 25
7.1 Screening and Eligibility Assessment ... 25
7.2 Investigational Medicinal Product Administration Visit ... 26
7.2.1 Vaccination Visit ... 26
7.2.2 Post Vaccination Visits ... 27
7.3 Additional Follow-up Visits ... 28
7.4 Final Study Visit or Early Termination Visit ... 28
7.5 Unscheduled Visits ... 28
8 STUDY PROCEDURES ... 28
8.1 Informed Consent Process ... 28
8.1.1 Consent for Future Use of Samples ... 30
8.2 Medical History and Physical Examination ... 30
8.3 HIV Testing and HIV- test Counselling ... 30
8.4 Combination prevention of HIV acquisition ... 31
8.5 Family Planning Counselling ... 31
8.6 Randomisation, blinding and unblinding ... 32
8.7 Un-blinding Procedure for Individual Volunteer ... 32
8.8 Sample Handling ... 32
9 INVESTIGATIONAL MEDICINAL PRODUCT (IMP) ... 33
9.1 IMP Description ... 33
9.2 Shipment and Storage of IMP ... 34

9.3 Preparation of Investigational Medicinal Product (IMP) ... 35
9.4 Administration of Investigational Medicinal Product ... 35
9.5 Accountability and Disposal of the Investigational Medicinal Product ... 35
10 ASSESSMENTS ... 36
10.1 Safety Assessments ... 36
10.1.1 Local reactogenicity ... 36
10.1.2 Systemic reactogenicity ... 36
10.1.3 Unsolicited Adverse Events ... 36
10.1.4 Assessment of Lymph Nodes ... 36
10.1.5 Vital Signs ... 37
10.1.6 Concomitant Medications ... 37
10.1.7 Routine laboratory parameters ... 37
10.1.8 Specific screening tests ... 38
10.2 Immunogenicity Assessments ... 38
10.2.1 Cellular Responses ... 38
10.2.2 Antibody Responses ... 38
10.2.3 PBMC, Serum and Plasma Storage ... 38
10.3 Other Assessments ... 39
10.3.1 HLA Typing ... 39
10.3.2 Microbiome Testing ... 39
10.3.3 HIV testing ... 39
10.3.4 Pregnancy Test ... 39
10.3.5 HIV Risk Assessment ... 39
10.3.6 Social Impact Assessment ... 39
11 SAFETY REPORTING ... 40
11.1 Definitions ... 40
11.2 Causality ... 41
11.3 Assessment of Severity of Adverse Events ... 42
11.4 Procedures for Recording Adverse Events ... 42
11.5 Reporting Procedures for Serious Adverse Events ... 42
11.6 Expectedness ... 43
11.7 SUSAR Reporting ... 43
11.8 Clinical Management of Adverse Events ... 43
11.9 Pregnancy ... 44
11.10 Intercurrent HIV Infection ... 44

11.11 SAFETY OVERSIGHT 45
11.11.1 Protocol Safety Review Team (PSRT) 45
11.11.2 Data Monitoring and Ethics Committee (DMEC) 45
11.11.3 Content of Interim Safety Review 45
11.11.4 Criteria for Pausing the Study 46
12 MANAGEMENT OF HIV ISSUES DURING AND FOLLOWING STUDY 46
12.1 HIV Testing 46
12.2 Social Discrimination as a Result of an Antibody Response to Vaccine 47
12.3 HIV Infection 47
12.3.1 Counselling 47
12.3.2 Referral for Support /Care 47
13 DISCONTINUATION OF VACCINATIONS AND/OR WITHDRAWAL FROM STUDY 48
13.1 Discontinuations of Vaccinations 48
13.2 Deferral of Vaccinations 49
13.3 Follow up after Discontinuation of Further Vaccinations 49
13.4 Withdrawal from the Study (Early Termination) 50
14 STATISTICS 50
14.1 Description of Statistical Methods 50
14.1.1 Analysis of demographics 50
14.1.2 Analysis of safety 50
14.1.3 Analysis of immunogenicity 51
14.2 The Number of Volunteers 51
14.3 The Level of Statistical Significance 53
14.4 Criteria for the Termination of the Trial 53
14.5 Procedure for Accounting for Missing, Unused, and Spurious Data. 53
14.6 Analysis Populations 53
14.6.1 Safety Population 53
14.6.2 Per Protocol Population (PP Population) 53
14.6.3 Unblinded Volunteers 54
14.7 Procedures for Reporting any Deviation(s) from the Original Statistical Plan 54
15 DATA MANAGEMENT 54
15.1 Source Data 54
15.2 Access to Data 54
15.3 Data Recording and Record Keeping 54
15.4 DATA ENTRY AT STUDY SITE 54

16 QUALITY ASSURANCE PROCEDURES 55
17 DATA AND BIOLOGICAL MATERIAL 55
18 ADMINISTRATIVE STRUCTURE 55
18.1 Study Supervision 55
18.2 Study Monitoring 55
18.3 Investigator's Records 56
19 SERIOUS BREACHES 56
20 ETHICAL AND REGULATORY CONSIDERATIONS 57
20.1 Declaration of Helsinki Guidelines for Good Clinical Practice 57
20.2 Approvals 57
20.3 Reporting 57
20.4 Volunteer Confidentiality 57
20.5 Expenses and Benefits 57
21 FINANCE AND INSURANCE 58
21.1 Funding 58
21.2 Indemnity 58
21.3 Insurance 58
22 PUBLICATION POLICY 58
23 REFERENCES 60
24 APPENDIX A: SCHEDULE OF PROCEDURES 64
25 APPENDIX B: AMENDMENT HISTORY 67
26 APPENDIX C: ADVERSE EVENT SEVERITY ASSESSMENT TABLE 68

1 KEY TRIAL CONTACTS

**Sponsor**		University of Oxford,<br>Research Services<br>University Offices<br>Wellington Square, Oxford, OX1 2JD<br>Email: oxtrec@admin.ox.ac.uk
**Clinical Trials Unit**		Center for Family Health Research in Zambia (CFHRZ)<br>B22/F737 Mwembelelo Road<br>Lusaka, Zambia<br><br>KAVI– Institute for Clinical Research (KAVI-ICR)<br>College of Health Sciences, University of Nairobi<br>PO Box 19676- 00202, Nairobi, Kenya<br><br>The KEMRI-Wellcome Trust Research Programme (KWTRP)<br>Kenya Medical Research Institute (KEMRI) / Wellcome Trust<br>Centre for Geographic Medicine Research – Coast (CGMRC)<br>PO Box 230, Kilifi, Kenya<br><br>MRC/UVRI Uganda Research Unit on AIDS (MRC/UVRI)<br>Medical Research Council/UVRI Uganda Research Unit on AIDS. Uganda Virus Research Institute Plot 5159 Nakiwogo Road, Entebbe, Uganda<br>P.O.BOX 49 Entebbe, Uganda
**Committees**		Scientific Advisory Board (SAB)<br>Prof Christian Brander<br>Institut de Recerca de la Sida – IrsiCaixa<br>Hospital Germans Trias I Pujol,<br>Barcelona, 08916<br>Spain<br>Tel: 0034 93 465674<br>Email: cbrander@irsicaixa.es<br><br>Oxford Tropical Research Ethics Committee (OxTREC)

		Chair: Prof Kevin Marsh<br>Named Contact: Dr Rebecca Bryant<br>University of Oxford<br>Research Services<br>University Offices<br>Wellington Square<br>Oxford, OX1 2JD<br>United Kingdom<br>Tel: +44 (0)1865 282585<br>e-mail: oxtrec@admin.ox.ac.uk
**Regulatory Authorities**		National Drug Authority (NDA), Uganda,<br>P. O Box 23096, Kampala, Uganda.<br>Plot 19 Lumumba Avenue (opposite TWED plaza).<br>Phone: +256 417 788 100.<br><br>Pharmacy and Poisons Board (PPB), Kenya,<br>P.O. Box 27663 – 00506, Nairobi.<br>Lenana Road Opposite DOD.<br>Phone: +254 720 608811.<br><br>Zambia Medicines Regulatory Authority (ZAMRA), Zambia,<br>P.O. Box 31890 Lusaka, Zambia.<br>Tuleteka Road, Off Makishi Road.<br>Phone: +260 211 220 429
**Funder**		European and Developing Countries Clinical Trials Partnership<br>PO Box 93015<br>2509 AA The Hague,<br>The Netherlands<br>Tel: +31 70 344 0880<br>Fax: +31 70 344 0899
**Laboratories**		The Jenner Institute laboratories<br>University of Oxford<br>Old Road Campus Research Building<br>Roosevelt Drive<br>Oxford OX3 7DQ<br>United Kingdom

Confidentiality Statement

This document contains confidential information that must not be disclosed to anyone other than the Sponsor, the Investigator Team, host organisation, and members of the Research Ethics Committee and other regulatory bodies. This information cannot be used for any purpose other than the evaluation or conduct of the clinical investigation without the prior written consent of Prof. Tomáš Hanke.

Statement of Compliance
The trial will be conducted in compliance with the protocol, the principles of Good Clinical Practice, Medicines for Human Use (Clinical Trial) Regulations 2004 (as amended) and all other applicable regulatory requirements.

Chief Investigator approval and agreement
I hereby approve this version of the protocol and declare no conflict of interest:

CI Details Dr. Paola Cicconi

**Name**	**Signature**	**Date**
Dr Paola Cicconi	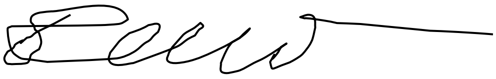	1st December 2021

PI details *(insert PI's Details)*

**Name**	**Signature**	**Date**

Modification History

Version	Date	Author(s)	Modifications
2.0	22 Jan 2020	Paola Cicconi	Section 1: Correction of Sponsor's contact details<br><br>Sections2 & 3: Clarification of first in human phase<br><br>Section 18.3: Clarification of the period Investigator records are be kept.<br><br>Table 9.1-1 Dose of placebo changed to 0.3-0.5 ml range in order to match the dose of the investigational vaccines.
2.1	6 May 2020	Alison Crook	Correction to section 7.2.1. The section on vaccination visits referred to Section 8.4. It has been corrected to refer to Section 9.4
3.0	14 Apr 2021	Mabela Matsoso and Vincent Muturi-Kioi	Page 2- Principal Investigators section: Change of site name from Zambia Emory HIV Research Project (ZEHRP) to Center for Family Health Research in Zambia (CFHRZ).<br><br>Section 1: Change of site name from Zambia Emory HIV Research Project (ZEHRP) to Center for Family Health Research in Zambia (CFHRZ).<br><br>Section 3.4: New rare risk of blood clotting associated with ChAdOx1 viral vector vaccine.<br><br>Section 6.3: Revised two exclusion criteria to include vaccinations using adenoviral vectored vaccines.<br><br>Section 15.4: Revised information on direct data entry.
4.0	01 Dec 2021	Vincent Muturi-Kioi	Page 27 – Addition of provision of a dose of paracetamol prior to administration of the MVA-vectored vaccines.

2 SYNOPSIS

INFORMATION	
**SYNOPSIS OF TRIAL HIV-CORE 006**	
**TITLE**	**HIV-CORE 006: A Phase 1 Trial of ChAdOx1- and MVA-vectored Conserved Mosaic HIV-1 Vaccines in Healthy, Adult HIV-negative Volunteers in Eastern and Southern Africa.**
**PHASE**	Phase 1 first-in-human trial
**SPONSOR**	University of Oxford
**HYPOTHESIS**	The mosaic immunogens delivered by a prime-boost regimen of non-replicating simian adenovirus followed by non-replicating poxvirus modified vaccinia virus Ankara (MVA) will induce $CD8^+$ T cells, specific for conserved epitopes common to HIV-1 variants, efficient in controlling HIV-1 infection or helping prevent establishment of chronic infection.
**OBJECTIVES**	**PRIMARY**<br>***Safety***<br>• To evaluate the safety and tolerability of a prime boost vaccine regimen utilizing non-replicating simian adenovirus (ChAdOx1) followed by non-replicating poxvirus modified vaccinia virus Ankara (MVA) in adults in Eastern and Southern Africa<br>***Immunogenicity***<br>• To evaluate the specific T-cell immune responses induced by the ChAdOx1.tHIVconsv1 followed by MVA.tHIVconsv3&4 vaccines in vaccine recipients.<br>**SECONDARY**<br>• To assess tHIVconsvX-specific T-cell responses of for their frequency, breadth and duration in vaccine recipients.<br>• To assess functional T-cell responses in vaccine recipients that inhibit replication in vitro of viruses of major HIV-1 clades A, B, C and D.<br>**EXPLORATORY**<br>• To assess induction of plurifunctional tHIVconsvX-specific memory T cells in the vaccine recipients.<br>• Characterization of the gut microbiome composition and richness.<br>• Feasibility of recruiting required sample size across all sites within 16 weeks<br>• Feasibility of retaining at least 90% of enrolled volunteers to end of study.
**ENDPOINTS**	**PRIMARY**<br>***Safety***<br>• Proportion of volunteers with local and systemic reactogenicity events from Day 0 to Day 7 post vaccination<br>• Proportion of volunteers with Grade 3 or 4 unsolicited adverse events through 28 days post final vaccination<br>• Proportion of volunteers with vaccine related serious adverse events (SAEs) collected throughout the study period<br>***Immunogenicity***<br>• Proportion of vaccine recipients developing HIV-1-specific T-cell responses<br>**SECONDARY**<br>• Frequency, breadth and duration of T-cell responses to conserved epitopes measured in IFN-γ ELISPOT assay in each vaccine recipient

<table>
<tr><td></td><td>

- Breadth of inhibition of HIV-1 viruses representative of circulating viruses in Kenya, Uganda and Zambia and other global clades in the *in vitro* Virus Inhibition Assay

EXPLORATORY

- Proportions of mono-, bi- and tri-functional T cells in the vaccine recipients
- Shotgun sequencing and metabolomic analyses.
- Proportion of target number of volunteers enrolled within 16 weeks
- Proportion of enrolled volunteers that are retained in the study through the final study visit.

</td></tr>
<tr><td>STUDY DESIGN</td><td>

<table>
<tr><th>Arms</th><th>N</th><th>Wk 0</th><th>Wk 4</th></tr>
<tr><td>1</td><td>72</td><td>C1</td><td>M3M4</td></tr>
<tr><td>2</td><td>16</td><td>Placebo</td><td>Placebo</td></tr>
<tr><td>Total</td><td>88</td><td></td><td></td></tr>
</table>

C1; ChAdOx1.tHIVconsv1
M3; MVA.tHIVconsv3
M4; MVA.tHIVconsv4

- All vaccinations will be given intramuscularly (IM), in the deltoid muscle of each arm.
- Follow up will occur for 48 weeks following enrolment (C1 vaccination).
- Unblinded study pharmacist(s) staff at each site will be responsible for investigational medicinal product preparation and accountability. All other site staff and all trial volunteers will be blinded to treatment assignment (i.e., active versus placebo).

Clinical Sites: Volunteers will be enrolled at 4 Clinical Research Centres in Kenya, Uganda, and Zambia, and randomised to receive vaccine or placebo.

</td></tr>
<tr><td>STUDY POPULATION</td><td>

Volunteers will include healthy HIV-uninfected male or female adults aged 18-50 years, who are at low risk of HIV infection, who are available for the duration of the trial, willing to undergo HIV testing, willing to use an effective method of contraception, and in the opinion of the principal investigator or designee, understand the study and provide written informed consent.

Principal exclusion criteria include confirmed HIV-1 or HIV-2 infection; pregnancy and lactation; significant acute or chronic disease; clinically significant laboratory abnormalities, recent vaccination or receipt of a blood product, previous receipt of an HIV vaccine; and history of severe local or systemic reactions to vaccination.

</td></tr>
<tr><td>DATA MONITORING AND ETHICS COMMITTEE (DMEC) AND PAUSING RULES</td><td>

The DMEC will review data from the first 10 volunteers at 2 weeks after vaccination with the prime (C1) and again at 2 weeks after the boost (M3M4).

PAUSING RULES

Enrollment will be paused if one or more volunteers experiences a Serious Adverse Event that is judged possibly, probably or definitely related to the IMP, if there is a volunteer death, if two or more volunteers experience grade 3 adverse events in the same System Organ Class that are considered to be at least possibly related to IMP, or if there is a grade 4 adverse event that is considered to be possibly, probably, or definitely related to IMP. The study will continue enrollment and vaccinations only after evaluation of relevant safety data by the DMEC.

</td></tr>
</table>

DESCRIPTION OF INVESTIGATIONAL MEDICINAL PRODUCT

- 5.0 x 10^{10} vp of ChAdOx1.tHIVconsv1 (C1) administered IM
- 1.0 x 10^{8} PFU of MVA.tHIVconsv3 (M3) administered IM
- 0.9 x 10^{8} PFU of MVA.tHIVconsv4 (M4) administered IM
- Normal sterile saline administered IM as Placebo

DURATION OF STUDY PARTICIPATION

Volunteers will be screened up to 28 days before the first vaccination and will be followed for 48 weeks after the first vaccine is administered. Total study duration for any individual volunteer will be approximately 52 weeks (4 weeks in the screening period and 48 weeks in the follow-up phase).

STATISTICAL CONSIDERATIONS

Safety Clinical safety data will be recorded using electronic data entry to allow central review of the entire database. At the end of the study, a full analysis will be prepared according to a pre-specified statistical analysis plan.
Tables 2 and 3 show the sample size calculations for safety, which are expressed in terms of the ability to detect related serious adverse events (SAE), i.e. the probabilities of observing 0 SAEs and 2+ (2 or more) related SAEs among a group of n=72 (any regimen) for a range of possible true event rates. For example, if none of the 72 volunteers receiving the vaccine experiences an SAE related to the vaccine, the 95% two-sided upper confidence bound for the rate of such reactions in a population exposed to the vaccines is 5%.

Table 2. Confidence in observed number of related SAEs (n = 72)

Observed number of related SAEs	95% Confidence Interval (exact)
0	0.0 – 5.0%
1	0.04 – 7.5%
2	0.3 – 9.7%
3	0.9– 11.7%
4	1.5 – 13.6%
5	2.3 – 15.5%

Table 3. Probability of observing 0 and 2+ related SAEs at a given SAE rate (n = 72)

Event rate (related SAE)	Any regimen (n=72)	
	0 events	2+ events
0.010	0.48	0.16
0.025	0.16	0.54
0.035	0.08	0.72
0.050	0.025	0.88
0.100	<0.001	0.995
0.150	<0.001	>0.999
0.200	<0.001	>0.999
0.250	<0.001	>0.999

Immunogenicity The primary immunogenicity endpoints are the frequency of vaccine responders.

The secondary endpoint is the frequency and breadth of HIV-1- specific T-cell responses induced by the vaccine in each individual and the number of HIV-1 viruses inhibited in a panel of HIV-1 after completion of the vaccination regimen. Inhibition is defined as a post-vaccination $\log_{10}$ reduction in HIV-1 production by $CD4^{+}$ cells by autologous $CD8^{+}$ effector cells of 1.5 or greater relative to pre-vaccination inhibition.

ABBREVIATIONS

AE	Adverse event
AR	Adverse reaction
CI	Chief Investigator
CRA	Clinical Research Associate (Study Monitor)
CRC	Clinical Research Centre
CRF	Case Report Form
CT	Clinical Trials
CTA	Clinical Trials Agreement
CTRG	Clinical Trials and Research Governance
DCC	Data Coordinating Centre
DMEC	Data Monitoring and Ethics Committee
DSUR	Development Safety Update Report
GCP	Good Clinical Practice
IB	Investigator's Brochure
ICF	Informed Consent Form
ICH	International Conference on Harmonisation
IEC	Independent Ethics Committee
IRB	Institutional Review Board
IMP	Investigational Medicinal Product
MHRA	Medicines and Healthcare products Regulatory Agency
MVA	Modified Vaccinia Ankara
OXTREC	Oxford Tropical Research Ethics Committee
PI	Principal Investigator
PIS	Participant Information Sheet
REC	Research Ethics Committee
SAE	Serious Adverse Event
SAR	Serious Adverse Reaction
SDV	Source Data Verification
SMPC	Summary of Medicinal Product Characteristics
SOP	Standard Operating Procedure
SUSAR	Suspected Unexpected Serious Adverse Reactions
TMF	Trial Master File
VISP	Vaccine-Induced Seropositivity

3 BACKGROUND AND RATIONALE

3.1. Urgent need for a vaccine against HIV infection/AIDS

Control of the HIV epidemic remains one of the global health priorities. Despite remarkable progress achieved in decreasing HIV transmission and AIDS-related deaths in the last decade due to development of over 30 antiretroviral drugs, HIV continues to spread, infecting approximately 1.8 million people in 2016(1). Around a third of people who are HIV positive do not know their status and hence do not receive treatment(1). For those who know their status: antiretroviral drugs may not be available on a regular reliable basis in many resource-poor settings(2). In addition to that, they have long-term side effects, their effective use requires rigorous daily compliance(3) and the circulating and/or transmitted viruses have been shown to develop resistance to several antiretroviral drugs. Also, there is unwillingness to take drugs in a surprisingly large proportion of infected individuals, even in the USA(4). Thus, a safe, effective, prophylactic HIV vaccine remains one of the priorities of HIV/AIDS research, and will be key to any strategy for halting the AIDS epidemic.

Phase 1 clinical trial HIV-CORE (COnserved REgions) 006 is a trial of a new combined vaccine regimen to determine safety and immunogenicity in healthy adults in Kenya, Uganda and Zambia. Immune responses may vary between populations and so it is important to confirm that the vaccines are suitable for the people and environment where they will be deployed for protection against HIV/AIDS. There are many different strains of HIV-1, and the virus can change to escape immune responses. This vaccine regimen is designed to work in all parts of the world.

Our aim is to induce effective cytotoxic T lymphocytes (CTL) against HIV-1. These could complement broadly neutralizing antibodies in prophylaxis and play a central role in cure. CTL exert their effector functions by killing HIV-1-infected cells and producing soluble factors, which directly or indirectly counteract the HIV-1 replicative cycle. In future, this approach could be combined, in human efficacy testing, with other immunogens that stimulate humoral responses, with the goal of effectively preventing HIV-1 infections.

The central principle of our strategy is to focus T-cell immune responses on the most conserved regions of the HIV-1 proteome. These regions are common to most variants and, if mutated, reduce the ability of the virus to grow(5); these regions are the “Achilles heel” of HIV-1. Targeting of conserved regions is further enhanced by using ‘mosaic’ proteins, which are designed by computer to maximize the match of the vaccine with global HIV-1 variants and to block common ways the HIV-1 changes to escape the immune response. Vaccines should match circulating HIV-1 variants as much as possible to stop them efficiently. When T cells attack conserved parts of HIV-1 proteins (parts that seldom or never change), the disease is better controlled—this vaccine includes those parts. The HIV-1-derived mosaic genes are called tHIVconsvX and are delivered by two safe, non-replicating vaccine vectors derived from chimpanzee adenovirus and poxvirus modified vaccinia virus Ankara (MVA). Adenoviruses, if able to grow, normally cause respiratory and gastrointestinal ailments, while the unmodified chimpanzee adenovirus is not known to cause disease in humans; the engineered vaccine vector called ChAdOx1 is crippled so it cannot grow. ChAdOx1 and similar experimental vaccines have been shown to be safe in over 1,500 human volunteers(6–8). MVA is a poxvirus, which does not replicate in humans. It was used safely as the smallpox vaccine in over 120,000 people at the end of the smallpox-eradication campaign and as an experimental vaccine vector against a variety of diseases in many clinical trials(9).

Humans and microbiota, which consist of bacteria, fungi, viruses and eukaryotic species, have co-evolved over millions of years and their coexistence is beneficial to both parties. Human immune system is constitutively exposed to microbial stimulation and any vaccine design and responsiveness needs to be considered in the context of host-microbiota interactions. Manipulation of the microbiota functions and composition through diet, engraftment and/or any other means may thus become a viable strategy for improving vaccine responsiveness as well as treating malfunctions of the immune system (10). The first reports on the influence of gut microbiota diversity and composition on responses to vaccination have

been emerging for some time (11–15). As part of the exploratory endpoints for this trial, we shall characterize the gut microbiome of study volunteers for composition and richness before and after administration of the study vaccines.

The ChAdOx1-vectored HIV-1 vaccine in this trial will be tested for the first time in humans, and MVA.tHIVconsv3 and MVA.tHIVconsv4 are currently administered to HIV-positive individuals in the first-in-human clinical trial in the USA (NCT03844386). However, this is the first time these C1-M3M4 vaccines will be administered to humans sequentially. In parallel with HIV-CORE 006, these vaccines will be tested for safety in the United Kingdom, in a phase I trial HIV-CORE 0052. The tHIVconsvX vaccines have been designed for global use irrespective of the HIV-1 strain and are therefore very suitable for Africa, where multiple strains are responsible for the epidemic, mainly from the HIV-1 families A, D and C. The HIV-CORE 006 trial will take place at four sites in Africa and will enroll healthy adults between 18 and 50 years of age. We do not yet know whether the vaccine will have a beneficial effect, no effect, or whether it could cause harm. The health of trial volunteers will be monitored carefully, and volunteers will receive counselling and support to minimize HIV-1 infection. Prior to the trial, consultations with policy makers, healthcare service providers and community leaders will take place to understand and address the obstacles and unmet demand for HIV services and healthcare services and to determine the appropriate level of baseline HIV-1-preventive services.

3.2. Investigational medicinal products

3.2.1. The investigational ChAdOx1.tHIVconsv1 vaccine

The ChAdOx1.tHIVconsv1 vaccine utilizes non-replicating engineered simian adenovirus vaccine vector ChAdOx1 derived from simian adenovirus (SAdV) serotype Y25 of chimpanzee origin to deliver a mosaic immunogen tHIVconsv1 derived from functionally conserved regions of HIV-1 proteins.

Rationale for using the ChAdOx1 vaccine vector. Selected types of attenuated adenovirus are very promising as highly immunogenic vaccine vectors(16, 17). A drawback for the use of the leading human adenovirus serotype 5 (HAdV-5) is pre-existing immunity, mainly neutralizing antibodies, found commonly in humans particularly in Africa(16–18). To circumvent this problem, a number of SAdV serotypes have been explored, which are unaffected by human pre-existing antibodies and display promising T-cell immunogenicity. The genomic DNA of these adenoviruses was cloned into bacterial plasmids, to eliminate any possibility of carrying over adventitious infectious agents from the original hosts. These viruses were rendered replication-incompetent by deletion of the E1 genomic region, their immunogenicity was increased by deletion of the E3 region and their genomes can stably accommodate passenger gene(s). HEK293 cells were used for preparation of high titre GMP virus stocks without the risk of generating contaminating replication-competent adenovirus (RCA) forms. For the 1st generation vaccines, we chose as the attenuated SAdV-derived vaccine vector ChAdV-63. In 2013, GSK acquired Okairos and the ChAdV-63 platform and withdrew further access to this vector. Hence, we switched to UOXF fully-owned ChAdOx1. Similarly to ChAdV-63, the vaccine vector ChAdOx1 is derived from E-group simian adenovirus, this time from serotype Y25 (19), which is at least equally safe and immunogenic to ChAdV-63 in mice (20) non-human primates and humans (21).

Rationale for the tHIVconsvX immunogens. The conserved HIV-1 regions of the 2nd generation vaccines used here are in total 872 amino acids (aa) in length and contain only 6 segments of the HIV-1 genome. The global-HIV-1-protein alignments curated by Los Alamos National Laboratory–HIV Sequence Database (LANL-HSD) *circa* September 2013, were used as the baseline data to define conserved regions. These alignments included only one sequence per individual, and only sequences spanning full-length proteins. Co-optimized, complementary pairs of two mosaic proteins (mosaic 1 and mosaic 2, which differ in 10% aa) were designed (22), spanning each HIV-1 protein. Conservation was defined by the capacity of the two mosaic proteins to have at least an 9/9-aa match to 80% of the potential 9-aa T-cell epitope (PTE) variants found among the diverse HIV-1 strains in the LANL-HSD alignments. The 80% match to group M HIV-1 isolates cutoff translated into a design of 6 segments from 29 to 333 aa long. Next, both beneficial and

detrimental epitopes as defined by Mothe *et al.*(23) were overlaid on vaccine-epitope-coverage maps for the final selection of regions for the 2nd generation conserved vaccine based on all criteria above, and the only regions included were *both* conserved and enriched for beneficial epitopes. Thus, the 2nd generation vaccine contained 33 of the 48 beneficial epitopes defined by Mothe *et al.*(23) (69%) - the missing 15 beneficial epitopes were in variable regions in p17 and Vif (not included). Env was deliberately excluded because of high variability and absence of beneficial epitopes. Based on the LANL epitope database, as of June 2015, 752 distinct CD8 T-cell epitopes restricted by 84 different HLA class I presenting molecules were contained in the six regions. While these numbers, based on the database summary of the literature, emphasize the rich immunogenic potential of the conserved regions we selected, we fully expect these regions will contain many as yet unknown epitopes(24). Finally, to avoid boosting with the same irrelevant cross-junctional epitopes, the six regions were arranged in different orders in six tHIVconsvX genes, of which tHIVconsv1, tHIVconsv3 and tHIVconsv5 coded for mosaic 1 and tHIVconsv2, tHIVconsv4 and tHIVcosnv6 coded for mosaic 2. Strong correlations of total tHIVconsvX-specific response magnitude and breadth with low viral load and high CD4 T-cell count were found in HIV-1-positive treatment naïve population in Japan.

Product Description; The ChAdOx1.tHIVconsv1 vaccine product will be supplied in sterile rubber-stopped glass vials. It will be presented as a slightly opaque solution, which will contain >1.1x10^{11} vp/ml of ChAdOx1.tHIVconsv1 with a total volume of 650 µl, of which 500 µl will be administered. The product will be stored frozen below -65 °C.

3.2.2. The investigational MVA.tHIVconsv3 and MVA.tHIVconsv4 vaccine components

Two vaccine components MVA.tHIVconsv3 and MVA.tHIVconsv4 complement each other and will be used as a pair for immunizations. They utilize non-replicating poxvirus MVA delivering bi-valent mosaic immunogens, either tHIVconsv3 or tHIVconsv4, derived from functionally conserved regions of HIV-1 proteins.

Rationale for using MVA as a vaccine vector; Modified vaccinia virus Ankara (MVA) is a vaccinia virus strain, which was attenuated by serial passage in chick embryo fibroblasts (CEF). It has lost 15% of the parental genome, including cytokine receptor genes. It replicates well in CEF and baby hamster cells but poorly in most mammalian cells (25, 26). MVA was used as a smallpox vaccine in the early 1970s towards the end of the eradication campaign in 120,000 people, without any serious adverse events reported, and is now licensed in the US for use in mass vaccination campaigns (Acambis 2000). Its safety in humans is therefore well established. MVA has been shown to be an effective vaccine vector and induce potent $CD8^+$ T-cell responses to passenger proteins. The immunogenicity of recombinant MVAs has been attributed in part to loss of several cytokine and chemokine receptor genes (27).

The safety and immunogenicity of a range of MVA-vectored vaccine candidates have been demonstrated in the BALB/c and SCID mice and in healthy and SIV-infected rhesus macaques, in GLP toxicology study UNO 0011 and in numerous preclinical studies (28–30). Similarly, the safety and immunogenicity of a range of MVA-vectored vaccine candidates for HIV-1 and other indications have been demonstrated in a number of clinical studies, in both healthy volunteers and HIV-1-infected volunteers (31–37).

Rationale for design of the tHIVconsvX immunogens; Please see above.

Product description MVA.tHIVconsv3 and MVA.tHIVconsv4 vaccine products will be supplied in separate sterile rubber-stopped glass vials. They will be presented as white cloudy solutions, formulated in Tris-HCl saline buffer (10 mM Tris-HCl, 140 mM NaCl, pH 7.7) at a concentration of 3.4 x 10^8 pfu/ml for MVA.tHIVconsv3 and 1.8x10^8 pfu/ml for MVA.tHIVconsv4, and an extractable volume of 500 µl for both. The products will be stored frozen below -65 °C.

3.3. A summary of findings from non-clinical studies and from other clinical trials

Testing of the 2nd generation conserved region mosaic vaccines is supported by clinical studies of the 1st generation conserved region vaccines, and studies of the 2nd generation regions in treatment naïve patients and animal models.

Thus, the 1st generation immunogen HIVconsv used 14 regions designed as clade alternating amino acid consensus (equivalent to a monovalent mosaic) (38). Vaccines ChAdV63.HIVconsv-MVA.HIVconsv tested in 8 clinical trials, showed promising immunogenicity (31–37) by producing a signal for viremic control in HIV-infected volunteers that were given early ART and 29% patients controlled viremia during monitored ART pause beyond the typical 4 weeks (spontaneous control occurs in 10%-15% patients) (36) .

Studies in 200 treatment-naïve HIV-1-positive Japanese patients demonstrated a statistically significant correlation between the total magnitude as well as the breadth of the CD8 T-cell responses specific for the conserved regions targeted by the 2nd generation vaccines and low plasma virus load and high CD4 counts (20) (39). All four correlations remained significant for 147 patients who did not have the HLA-B52 and B67 alleles protective in the Japanese population, suggesting that our vaccines could be effective in the entire population rather than only those who have protective HLA class I alleles.

Preclinical studies demonstrated high immunogenicity of the 2nd generation vaccines in mice and also of SIV-derived equivalent immunogens delivered by the same ChAdOx1-MVA vectors in rhesus macaques.

3.4. Summary of the known and potential risks and benefits, if any, to human volunteers

Risks of chimp adenovirus vaccination. This vaccine adenovirus is weakened so that it cannot multiply in humans. ChAdOx1 is being developed as a vaccine vector for a number of vaccine candidates such as chikungunya, MERS, influenza, tuberculosis, malaria, meningitis (Men B) and cancer. While the ChAdOx1 vectored HIV-1 vaccine in this trial will be tested for the first time in humans, previous vaccine candidates using this vector were found to be well tolerated. Trials with these vaccines revealed that the majority of local and systemic reactogenicity related to the use of this vector has been mild or moderate. Cases of severe (Grade 3) reactogenicity have been noted with doses of 2.5 x 10^{10} vp and 5 x 10^{10} vp and included local pain, fever, nausea, malaise, fatigue, headache, arthralgia and myalgia. Laboratory adverse events have also been noted, these were of mild severity including lymphopenia, leukopenia, neutropenia, and anemia.
The 1st generation mosaic vaccine ChadV63.HIVconsv was tested in volunteers in the United Kingdom, continental Europe and in Africa and was well-tolerated with a positive safety profile. The majority of local and systemic adverse reactions were of mild to moderate severity with severe (Grade 3) reactogenicity being noted with doses of 5 x 10^{10} vp including local pain, fever, arthralgia, headache, fatigue and malaise.

Other potential rare severe reactions. The ChAdOx1 part of the vaccine (the “viral vector” or “carrier”) is the same as has been used in a recently developed COVID-19 vaccine (ChAdOx1 nCoV-19 - commonly known as the Oxford/AstraZeneca vaccine/Vaxzevria or Covidshield). In March 2021, concerns were raised after observations pointed to rare blood clotting conditions that could be associated with the vaccine. These events were reviewed by the MHRA (Medicines and Healthcare products Regulatory Agency) and the EMA (European Medicines Agency). The reports were of a very rare type of blood clot in the brain, known as cerebral venous sinus thrombosis (CVST), and also of clots in some other organs together, with low levels of platelets (thrombocytopenia). Up to and including 31 March 2021 there have been 79 UK reports of these blood clots and unfortunately 19 people died. Additional reports were submitted to the EMA from across the European Union. By 31 March 2021, 20.2 million doses of the ChAdOx1 nCoV-19 vaccine had been given in the UK.

After investigation, the UK MHRAconcluded, based on the data currently available to them, they could not say that there was a definite link between the vaccine and the rare clotting events. The European

Medicines Agency concluded that unusual blood clots with low blood platelets should be listed as very rare side effects of the ChadOx1 nCov-19 vaccine.

Both agencies concluded that there wasn't enough evidence at present to say what the risk factors (e.g. age, gender, or other medical conditions) might be for having one of these rare clotting problems.

It is not known whether these rare clotting problems might be related to the vaccine vector virus (ChAdOx1), or to the SARS-CoV-2 part of the vaccine, the spike protein (the insert). The ChAdOx1 vector has been used in other clinical trials since 2012 (influenza, tuberculosis, prostate cancer, malaria, meningitis B, chikungunya, Zika and HIV vaccine trials). These rare blood clotting problems have not been seen in participants in these trials, however the number of people in these trials has been relatively small. These events remain extremely rare (in the UK it is estimated to affect 4 in a million people who receive a vaccine dose), and all medical regulators are collecting and analysing further data on them.

Risks of poxvirus MVA vaccination. The MVA vector was used as a smallpox vaccine about 40 years ago in over 120,000 people, without any reported serious adverse events during the vaccination campaign. Currently, MVA is being developed as a vaccine vector for a number of diseases such as HIV, malaria, tuberculosis, hepatitis, influenza and cancer. While the two MVA vaccines in this trial will be tested for the first time with ChAdOx1.tHIVconsv1 in humans. M&M Clinical Trial (NCT03844386) is the first-in-human clinical trial using MVA.tHIVconsv3 and MVA.tHIVconsv4. This is a double blind, randomized, placebo-controlled, parallel design, study in which 24 HIV-infected participants with durable viral suppression will be randomly assigned to receive vaccination with MVA.tHIVconsv3 (M3), MVA.tHIVconsv4 (M4), M3+M4 combined, or placebo. To date, 5 individuals were enrolled; no SAEs were recorded. Two other HIV vaccines developed by the same team in the University of Oxford, namely MVA.HIVA and the 1st generation conserved-region MVA.HIVconsv vaccines were tested in 370 and 188 healthy people, respectively. These vaccines were found to be safe and did not cause any significant or serious side effects in HIV-1 positive vaccinees on ART and HIV-negative vaccinees in the United Kingdom, continental Europe and Africa.

For the reasons given above, we do not expect to see any serious adverse reactions to the vaccines given in this study. Although no serious side effects were observed in experimental animals receiving these vaccines, it is possible that vaccines may produce unexpected and new side effects in some humans.

Any vaccination has the potential to cause a temporary soreness around the injection site; redness, pain, swelling, itching, bruising, a warm feeling; flu-like symptoms such as fever, chills, muscle aches and pains, headaches, nausea, dizziness and fatigue.

With any vaccination, there is a risk of rare serious adverse events, like an allergic reaction, characterized by rash, low blood pressure, sudden body swelling or serious breathing difficulty. Severe reactions in the nervous system are also extremely rare following vaccination and can cause Guillain-Barré syndrome (GBS). However, neither wild-type human adenoviruses nor vaccinia infections are associated with an increased risk of GBS, therefore, the possibility of this occurring as a result of vaccination with replication-defective viral vectors is extremely remote.

Vaccine-Induced Seropositivity (VISP). Administration of HIV vaccine candidates may cause some HIV-1 tests to give false positive results. Previous experience with the mosaic vaccines in mice has not demonstrated their ability to produce antibodies that could be detected by diagnostic antibody tests.

If VISP occurs, different testing methods will be used that will be able to distinguish true HIV infection from a false result due to vaccination.

3.5. Route of administration, dosage, dosage regimen and vaccination period

The vaccines will be administered into the deltoid region of each arm. The total doses employed as well as the heterologous-prime-boost chimp adenovirus-MVA regimen have been tested and optimized for these types of vaccine vectors in many human volunteers for HIV-1 and other disease candidate vaccines. The volunteers will receive two doses of vaccines; at enrolment, then at 4 weeks. The 4-week gap is tested to assess an accelerated immunization protocol, which would be advantageous in a subsequent proof-of-concept phase 2b efficacy trial. The 4-week interval was shown to be equivalent to the 8-week interval in the HIV-CORE 003 trial and studies with Ebola vaccines (AVS Hill, UOXF; personal communication). Volunteers will be followed for 48 weeks from enrolment at week 0.

3.6. Study population

The purpose of this trial is to determine whether the vaccination regimen is safe, tolerable and immunogenic. The vaccine immunogens tHIVconsvX were designed for maximum match with group M HIV-1 covering isolates from US/Europe and Africa. Previous studies have shown differences in African populations with respect to immune responses (40). This study will test the specificity, magnitude and functionality of T-cell responses after a regimen of one dose of ChAdOx1.tHIVconsv1 prime followed by MVA.tHIVconsv3 and MVA.tHIVconsv4 as a boost.

Enrollment for the HIV-CORE 006 trial will take place at four Clinical Research Centres (CRCs) in Eastern and Southern Africa. The target study population will include women and men at low risk of HIV infection.

4 OBJECTIVES AND OUTCOME MEASURES

Objectives	Endpoints/Outcome Measures	Timepoint(s) of evaluation of this outcome measure (if applicable)
**Primary Objective**<br><br>• To evaluate the safety and tolerability of a prime boost vaccine regimen utilizing non-replicating simian adenovirus (ChAdOx1) followed by non-replicating poxvirus modified vaccinia virus Ankara (MVA) in adults in Eastern and Southern Africa<br><br>• To evaluate the specific T-cell immune responses induced by the ChAdOx1.tHIVconsv1 followed by MVA.tHIVconsv3&4 vaccines in vaccine recipients	• Proportion of volunteers who experience local and systemic reactogenicity events<br>• Proportion of volunteers with Grade 3 or 4 unsolicited adverse events<br>• Proportion of volunteers with vaccine related serious adverse events (SAEs)<br><br>• Proportion of vaccine recipients developing HIV-1 specific T-cell responses	• Within 7 days post vaccination<br>• Through 28 days post final vaccination<br>• Throughout the study period<br><br>• At 1 and 40 weeks post final vaccination
**Secondary Objectives**<br>To assess:<br>• tHIVconsvX-specific T-cell responses for their frequency, breadth and duration in vaccine recipients<br><br>• Functional T-cell responses in vaccine recipients that inhibit replication *in vitro* of viruses of major HIV-1 clades A, B, C and D	• Frequency, breadth and duration of T-cell responses to conserved epitopes measured in the IFN-γ ELISPOT assay in each vaccine recipient<br><br>• Breadth of inhibition of HIV-1 viruses representative of circulating viruses in Kenya, Uganda and Zambia and other global clades in the in vitro Virus Inhibition Assay	• At 1 and 40 weeks post final vaccination<br><br>• At 1 and 40 weeks post final vaccination
**Exploratory Objectives**<br>• To assess induction of plurifunctional tHIVconsvX-	• Proportions of mono-, bi- and tri-functional T cells in the vaccine recipients	• At 1 and 40 weeks post final vaccination

Objectives	Endpoints/Outcome Measures	Timepoint(s) of evaluation of this outcome measure (if applicable)
specific memory T cells in the vaccine recipients		
• Characterization of the gut microbiome composition and richness	• Shotgun sequencing and metabolomic analyses	• Before vaccination, at 1 and 44 weeks post final vaccination.
• Feasibility of recruiting required sample size across all sites within 16 weeks	• Proportion of target number of volunteers enrolled within 16 weeks	• 16 weeks post first enrolment
• Feasibility of retaining at least 90% of enrolled volunteers to end of study.<br>•	• Proportion of enrolled volunteers that are retained in the study through the final study visit.	• End of study

5 TRIAL DESIGN

HIV-CORE 006 is a double-blind placebo-controlled trial, in which the mosaic immunogens are delivered by a prime-boost regimen of non-replicating simian adenovirus followed by non-replicating poxvirus MVA. Volunteers will be randomised to receive either the vaccine regimen or placebo at 2 vaccination visits 4 weeks apart. The vaccine regimen consists of a single mosaic prime ChAdOx1.tHIVconsv1 (C1) and a dual boost of MVA.tHIVconsv3 (M3) and MVA.tHIVconsv4 (M4) administered simultaneously. The trial will recruit healthy African adults 18-50 years of age, who are HIV-uninfected and at low risk of HIV infection.

The trial is designed to enrol 88 healthy men and women, who will be randomised to receive either the vaccine regimen or placebo in a ratio of 72:16:

- Vaccine Arm (ChAdOx1.tHIVconsv1 prime followed by MVA.tHIVconsv3 and MVA.tHIVconsv4 boost at 4 weeks after enrolment); 72 vaccine recipients;
- Placebo Arm; 16 recipients

To maintain blinding, all volunteers will receive two injections with half dose into the deltoid region of each arm of ChAdOx1.tHIVconsv1 or placebo at enrolment, and two injections (MVA.tHIVconsv3 or placebo into one deltoid region and MVA.tHIVconsv4 or placebo into the other) at 4 weeks after enrolment. The primary goal of assessing safety and immunogenicity will be served by weighting the randomisation toward vaccinees.

Enrolment of all volunteers into HIV-CORE 006 is expected to take up to 16 weeks. Participation in the trial for each volunteer will be approximately 52 weeks and all volunteers will be followed up for 48 weeks after first vaccination.

Data will be collected from each volunteer from screening, enrolment and every scheduled and unscheduled visit on CRFs. CRFs will be entered into the electronic database system.

6 VOLUNTEER IDENTIFICATION

6.1 Study Population

The study population consists of healthy HIV-uninfected male or female adults aged 18-50 years, who are at low risk of HIV infection, based on a HIV risk assessment at screening, in Kenya, Uganda and Zambia.

6.2 Inclusion Criteria

- Healthy male and female as assessed by a medical history, physical exam, and laboratory tests.
- At low risk of HIV infection and willing to maintain low-risk behaviour for the duration of the trial. Individuals from key populations are not excluded provided they are assessed to be at low risk of HIV infection at screening and are willing to maintain low-risk behaviour during the study.
- At least 18 years of age on the day of screening and have not reached their 51st birthday on the day of the first vaccination.
- Willing and able to give informed consent for participation in the trial before any study-related procedures are performed. Volunteers will pass an Assessment of Understanding before signing the consent form.
- Willing to comply with the requirements of the protocol and be available for follow up for the planned duration of the study.
- Willing to undergo HIV testing, risk reduction counselling, receive HIV test results
- All sexually active males (unless anatomically sterile or in a monogamous relationship with a female partner who uses a documented non-barrier method of birth control) must be willing to use an effective method of contraception (such as consistent condom use) from the day of first vaccination until 4 months after the last vaccination.
- If a female of childbearing potential, willing to use an effective non-barrier method of contraception (hormonal contraceptive or intrauterine device) from at least 2 weeks prior to first vaccination until at least 4 months after the last study vaccination. If not of childbearing potential: postmenopausal (>45 years of age with amenorrhea for at least 2 years, or any age with amenorrhea for at least 6 months and a serum follicle stimulating hormone [FSH] level >40 IU/L) or surgically sterile: no additional contraception required.
- All female volunteers must be willing to undergo urine pregnancy tests at time points indicated in the Schedule of Procedures and must test negative prior to each study vaccination.
- Willing to forego donations of blood or any other tissues during the trial and, for those who test positive for HIV antibodies due to vaccination (vaccine-induced seropositivity/ reactivity), until the anti-HIV antibody titres become undetectable.

6.3 Exclusion Criteria

- Confirmed HIV-1 or HIV-2 infection
- Receipt of any vaccine in the previous 28 days or planned receipt within 28 days of Investigational Medicinal Product. Volunteers who expect to receive any adenoviral vectored vaccines within the next three months after ChAdOx1.tHIVconsv1 vaccine administration should not participate due to the risk of immune interference with the study vaccine.
- Participation in another clinical trial of an Investigational Medicinal Product currently, within the previous 3 months or expected participation during the study.

- Previous receipt of another investigational HIV vaccine candidate, or any adenoviral vectored vaccine (Note: receipt of an HIV vaccine placebo will not exclude a volunteer from participation if documentation is available and the Medical Monitor gives approval).
- Receipt of blood transfusion or blood-derived products within the previous 4 months or expectation of receiving blood products during the study period.
- Receipt of immunoglobulin products within the previous 3 months.
- If female, pregnant or planning a pregnancy during the period of enrolment until 4 months after the last study vaccination; or lactating.
- Any clinically relevant abnormality on history or examination such as:
    - Any confirmed or suspected history of immunodeficiency including recurrent severe infections;
    - Use of systemic corticosteroids for >14 days (use of topical or inhaled steroids is permitted) within the previous 6 months;
    - Immunosuppressive, anti-cancer, anti-tuberculosis or other medications considered significant by the investigator within the previous 6 months.
    - History of splenectomy.
    - History of autoimmune disease
    - Bleeding disorder diagnosed by a physician (e.g., factor deficiency, coagulopathy or platelet disorder that requires special precautions). (Note: a volunteer that states that he or she has easy bruising or bleeding but does not have a formal diagnosis and has intramuscular injections and blood draws without any adverse experience is eligible)
    - History of myocarditis, pericarditis, cardiomyopathy, congestive heart failure with permanent sequelae, or clinically significant arrhythmia (including any arrhythmia requiring medication, treatment, or clinical follow up).
    - History of cancer (except basal cell carcinoma of the skin)
    - Asthma that is not well controlled.
    - History of allergic disease or reactions likely to be exacerbated by any component of the vaccine
    - History of severe local or systemic reactogenicity to vaccines (e.g., anaphylaxis, respiratory difficulty, angioedema).
    - History of Guillain-Barre syndrome.
    - Confirmed diagnosis of active or chronic hepatitis B, hepatitis C, active syphilis and/or active tuberculosis
    - Seizure disorder: an individual who has had a seizure in the last 3 years is excluded (Not excluded: an individual with a history of seizures who has neither required medication nor had a seizure for three or more years).
    - History of serious psychiatric conditions or any psychiatric condition that precludes compliance with the protocol. Specifically excluded are persons with psychoses, ongoing risk for suicide, or history of suicide attempt or gesture within the past 3 years.
    - Substance abuse disorder that precludes compliance with the protocol as assessed by the investigator.
    - Any clinically significant acute or chronic medical condition that is considered unstable/progressive, or in the opinion of the Investigator, may either put the volunteer at risk because of participation in the study, or may influence the result of the study, or the volunteer’s ability to participate in the study

- Abnormal clinically significant abnormal finding on screening biochemistry or haematology blood, or urinalysis, including but not limited to:
  <u>Haematology:</u>

    - Haemoglobin - <9.5 g/dl in females; <11.0 g/dl in males
    - Absolute Neutrophil Count (ANC) – ≤1,000/mm^3
    - Absolute Lymphocyte Count (ALC) – ≤650/mm^3
    - Platelets - <100,000 cells/mm^3

  Chemistry

    - Creatinine >1.1 x upper limit of normal (ULN)
    - AST >1.25 x ULN
    - ALT >1.25 x ULN

  Urinalysis

  Clinically significant abnormal dipstick confirmed by microscopy:

    - Protein = 2+ or more
    - Blood = 2+ or more (not due to menses)

  - Any other finding which in the opinion of the investigators would significantly increase the risk of having an adverse outcome from participating in the study
- If, in the opinion of the Principal Investigator, it is not in the best interest of the volunteer to participate in the trial.
- **NOTE:** Investigators should ensure that all study enrolment criteria have been met at the end of the screening period. If a volunteer's status changes at any time before the 1st dose of the study drug is given that they now meet an exclusion criterion, they should be excluded from participation in the study.

6.4 Recruitment of Volunteers

Healthy adult male and female volunteers may be recruited through information presented in community organizations/settings, other institutions and/or advertisements to the general public or from existing cohorts. Each site will devise and use locally effective practices to recruit from the specific populations. All materials to be distributed to the volunteers will be submitted for approval by the Ethics Committee before they are used. Efforts will be made towards overall gender balance as a composite of enrolled volunteers.

7 TRIAL VISITS

7.1 Screening and Eligibility Assessment

Screening assessments will be performed up to 28 days before enrolment. Screening procedures will only be performed after the volunteer's written consent has been obtained and may be conducted over multiple days during the screening period.

Before screening, prospective volunteers will receive a study number. At screening, demographic and risk factor information will be collected, and HIV testing will be performed. Volunteers who are identified as being HIV-infected will be referred for further HIV treatment and care.

Reasons for screening failure will be obtained from volunteers who are not enrolled including those who are eligible but not enrolled. The procedures at this visit are summarized below and will be conducted:

- Provide and or review the Informed Consent Form with the volunteer and answer any relevant question that the volunteer may have about the study.

- Complete Assessment of Informed Consent Understanding (AOU). Please refer to the Study Operations Manual (SOM)

  If the volunteer agrees to participate, passes the AOU and provides written informed consent, study staff will:
- Obtain written informed consent before performing any study procedures.
- Perform HIV testing, HIV test counselling and risk reduction counseling
- Administer HIV risk assessment
- Conduct family planning counselling, refer for contraceptive counseling if necessary
- Take a comprehensive medical history.
- Collect concomitant medication information.
- Perform a general physical examination including assessment of height, weight and vital signs;
- Collect specimens for all tests as indicated in the Schedule of Procedures see Appendix A (for details see the Laboratory Analytical Plan (LAP).

When available, the screening laboratory tests will be reviewed by the trial physician. Screening laboratory test(s) may be repeated <u>once</u> at the discretion of the Principal Investigator or designee to investigate any isolated abnormalities.

If the screening visit occurs more than 28 days prior to the date of vaccination, all screening procedures must be repeated. In such cases, the comprehensive medical history may be replaced by an interim medical history and the Informed Consent Form should be reviewed.

If a volunteer has signed the Consent Form but does not meet the eligibility criteria, the records must be kept at the site.

7.2 Investigational Medicinal Product Administration Visit

7.2.1 Vaccination Visit

Volunteers will receive either a vaccine or placebo depending on the randomisation schedule. These visits are specified in the Schedule of Procedures (Appendix A).

Prior to the first vaccination, site personnel will:

- Review any questions the volunteer may have about the study
- Review the Informed Consent Form with the volunteer
- Review screening safety laboratory data
- Obtain pregnancy test results prior to investigational medicinal product administration
- Perform HIV testing, HIV test counselling and risk reduction counseling
- Administer HIV risk assessment
- Conduct family planning counselling as per site specific procedures and ensure compliance with respective contraceptive method
- Review interim medical history
- Collect concomitant medication information
- Assess baseline vital signs (blood pressure, heart rate, respiratory rate and temperature)

- Perform a general physical examination
- Assess cervical and axillary lymph nodes
- Assess for the presence at baseline of any signs and symptoms related to the solicited local and systemic reactogenicity/solicited adverse reactions that will be assessed after vaccination. This includes an examination of the administration site.
- Collect specimens for all tests as indicated in the Schedule of Procedures Appendix A
- Assign an allocation number to the volunteer according to the instructions specified in the Study Operations Manual.
- Administer the Investigational Medicinal Product as specified in Sectiona 9.4. Administration of Investigational Medicinal Product and according to instructions in the Study Operations Manual.
- Observe volunteer closely for at least 30 minutes after administration for any acute (reactogenicity/solicited adverse reactogenicity /solicited adverse reactions) and train the volunteer on using the memory aid
- Assess any other adverse events

Subsequent vaccine/investigational medicinal product administration visits:
Study staff will perform the same procedures as above with the following additions:

- Review the routine safety laboratory parameters as appropriate from previous visit prior to each vaccine administration, if a volunteer has an abnormal value that is known at the time of vaccination, follow the specified guidelines (Section 12.0)
- Conduct HIV test counselling if an HIV Test is scheduled (Appendix A) or clinically indicated.
- It is recommended that all participants receive a dose of paracetamol prior to administration of the MVA-vectored vaccines. This recommendation is based on a high rate of adverse reactions, including severe (Grade 3) adverse reactions, noted following administration of the MVA-vectored vaccines.

Volunteers will receive investigational medicinal products at timepoints as specified in the schedule of procedures. If volunteers are seen outside the product administration visit windows, administration of IMP may be done in consultation with the Medical Monitor. All unscheduled visits will be documented in the volunteers' study records on applicable source documents and entered into the Case Report Form (CRF).

7.2.2 Post Vaccination Visits

The volunteer will be asked to return to the clinic on Day 7 (+/- 3 days), Day 14 (+/- 3 days) and Day 28 (+/- 3 days) after each vaccination for an assessment by clinic staff. A telephone contact or clinic visit will be conducted after each vaccination according to the Schedule of Procedures (Appendix A).

The volunteer will be asked to maintain a Memory Aid from the day of each vaccination and for the next 7 days. Study staff will review the Memory Aid and solicit local and systemic reactogenicity with the volunteer to determine the severity of the reactions through discussions with the volunteer.

The following procedures will be conducted at these visits:

- Review and record interim medical history
- Collect concomitant medication

- Perform a symptom directed physical examination including vital signs (pulse, respiratory rate, blood pressure and temperature), examination of vaccination site(s) and cervical and axillary lymph nodes in addition to any further examination indicated by history or observation
- Solicit and record serious and non-serious adverse events
- Obtain a blood sample for laboratory investigations in keeping with the schedule of procedures (Appendix A) and the Laboratory Analytical Plan

7.3 Additional Follow-up Visits

Assessments and procedures will be performed according to the Schedule of Procedures (Appendix A)

7.4 Final Study Visit or Early Termination Visit

The Final Visit or Early Termination Visit procedures will be performed according to the Schedule of Procedures (Appendix A). Volunteer retention strategies and procedures must be established at the site with a goal to achieve an annual retention rate of at least 90%. Volunteers may voluntarily withdraw from the study at any time and for any reason. Volunteers may be withdrawn from the study permanently if:

- The principal investigator(s) or designee has reason to believe that a volunteer is not complying with the study or study procedures.
- The sponsor or local regulatory authorities decide to stop or cancel the study.
- Volunteer is lost to follow up.

If a volunteer withdraws from the study, the date and reasons for study withdrawal (if available) will be recorded in the volunteer's source documents and study records and stored samples will be retained unless consent is withdrawn.

7.5 Unscheduled Visits

Visits / contacts other than those described in the Schedule of Procedures (Appendix A) will be classified as unscheduled visits and recorded on applicable source documents and entered into the designated CRF. They may occur:

- For administrative reasons e.g. the volunteer may have questions for the study staff or may need to re-schedule a follow up visit
- To review a laboratory investigation from a previous visit
- To review the outcome of an adverse event
- To conduct a study, visit where a volunteer has missed the scheduled study visit window
- For any other reason requested by the volunteer or Principal Investigator

8 STUDY PROCEDURES

8.1 Informed Consent Process

The study site staff is responsible for developing study informed consent documents for local use based on the core informed consent form developed by the sponsor/ designee. This document is translated (if necessary) submitted and approved by an Independent Ethics Committee (EC) Institutional Review Board (IRB).

The person obtaining the consent will be suitably qualified and experienced and will have been authorised to do so by the Principal Investigator.

All volunteers will give their consent to participate in the study on the basis of appropriate information and with adequate time to consider this information and ask questions. The volunteers will be allowed as much time as they wish to consider the information, and the opportunity to question the Investigator or other independent parties to decide whether they will participate in the trial. Prior to enrollment, the consent form will be reviewed with the volunteer. The informed consent form will be reviewed with them in person to enable discussion of: the exact nature of the study; the implications and constraints of the protocol; the known side effects and any risks involved in taking part. It will be clearly stated that the volunteer is free to withdraw from the study at any time for any reason without prejudice to future care, and with no obligation to give the reason for withdrawal. After reading the informed consent form (ICF), the assessment of understanding (AOU) will be administered. Volunteers who fail the AOU may repeat the test only once. Volunteers must pass the AOU before signing the ICF.

Written Informed Consent will then be obtained by means of a dated volunteer signature and dated signature of the person who presented and obtained the informed consent. If the volunteer is functionally illiterate, the consent document must be read to them in the language that they best understand in the presence of a literate impartial witness who will not be evaluating the volunteer directly and who will sign and date the informed consent as a witness. The signed/marked and dated informed consent form must be stored at the study site for verification. A copy of the signed/marked and dated informed consent form will be offered to the volunteer to take home if the volunteer is willing. If not, the volunteer's copy will be filed at the site**.**

Volunteers will be informed prior to consenting that they are free to withdraw from the study at any time without any penalty or loss of benefits that, otherwise, they would have been entitled to receive. This includes, but is not limited to, social and medical benefits as well as future care at the study site or clinic. Volunteers will be informed about any changes to the study, if any, through an amended informed consent.

8.1.1 Consent for Future Use of Samples

Volunteers may also provide consent for the long-term storage of their research samples for possible use in future HIV or other disease research. These sample will be de-identified and archived, and the testing laboratories will be blinded to the volunteer's identity. Only tests approved by an appropriate institutional Review Board (IRB)/ Independent Ethics Committee (IEC) will be performed. Volunteers could withdraw consent for long-term storage at any time before de-identification of samples by contacting the study team. If volunteers choose not to allow the future use of the remaining samples, they will be destroyed at the end of the trial, in accordance with local laws and SOPs.

8.2 Medical History and Physical Examination

Medical History

At screening, a comprehensive medical history will be collected including previous vaccinations and reaction to vaccinations, history of STI and contraceptive practices. At subsequent visits, an interim medical history will be performed.

Physical Examination

General Physical Examination

A general physical examination includes examination of skin, respiratory, cardiovascular, abdominal, limited neurological, musculoskeletal, systems, general appearance, Head/ENT (Ear Nose and Throat), neck and extremities at the time points indicated in the schedule of procedure (see Appendix A)

Symptom-Directed Physical Examination

A symptom-directed physical examination includes the examination of any systems that are indicated by history or observation. This examination is done at the time points indicated in the schedule of procedure (see Appendix A)

Measuring Height and Weight

Includes measuring the height and weight at the time points indicated in the schedule of procedure (see Appendix A)

Vital Signs

Vital signs including blood pressure, heart rate, respiratory rate, blood pressure and temperature are measured and recorded at the time points indicated in the Schedule of Procedures (see Appendix A)

8.3 HIV Testing and HIV- test Counselling

Trained and qualified study personnel will provide HIV counseling and testing at designated study visits as indicated in the schedule of procedures (Appendix A), and it will be performed according to a predefined, approved SOP. Counseling will be provided in compliance with national guidelines and locally accepted

standards of practice. Study sites will document their counseling policies and procedures prior to study initiation for purposes of staff training, QA, and study monitoring.

The counseling process will, at a minimum, include information on HIV, safe sex practices and risk reduction. The objective of counseling is to ensure that volunteers have sufficient knowledge about HIV infection to understand what the test is for, the implications of a positive or negative result and the care available for HIV infection locally. Additionally, risk reduction counseling, safe sex practices, proven methods of preventing HIV acquisition and avoidance of transmission to others will be discussed with all volunteers regardless of their HIV test results.

Condoms and other HIV prevention supplies will be offered to volunteers at each visit. Volunteers who are infected with HIV at screening and during follow up will be counseled and referred for care and treatment as needed. Counseling for HIV infected volunteers will include:

- Psychological and social implications of HIV infection
- Implications for sexual partners and family members
- Implications for child-bearing
- Avoidance of transmission to others in future
- Where to obtain HIV counseling, care and treatment

8.4 Combination prevention of HIV acquisition

Site counsellors will provide HIV risk reduction counselling based on reported individual risk at time points specified in the Schedule of Procedures (Appendix A) and provide free condoms. The procedures for risk reduction counselling will be detailed in site-specific SOPs. In addition, regular testing as well as treatment for sexually transmitted infections (STIs) will be provided. Counselling and referral for post exposure prophylaxis (PEP) will be provided when indicated. Information and referral for male circumcision will be carried out as well.

Oral pre-exposure prophylaxis (PrEP) is a HIV prevention option. Following national guidelines at the individual study sites, volunteers will receive information about the protection from HIV infection by oral PrEP. Study sites will facilitate access to PrEP services through referral to or providing PrEP on-site where available. At a minimum, sites will provide information on PrEP and refer interested volunteers to PrEP providing service providers or demonstration projects where they exist. Alternatively, where these services are in place, the clinical research centres may provide PrEP at the study centre. Volunteers will be informed as new prevention modalities are proven effective and become available.

8.5 Family Planning Counselling

Study staff will counsel volunteers about the importance of preventing pregnancies and use of condoms as well as other effective family planning methods, as appropriate. Volunteers may be referred for family planning services either on-site or to a family planning clinic, as necessary and according to site-specific SOPs as detailed in the SOM. Contraceptive methods chosen and compliance will be documented.

Women of childbearing potential (e.g., not menopausal or surgically sterile) must use an effective, non-barrier form of family planning, such as hormonal methods or an intrauterine device, from one month prior to vaccination to at least 4 months after receiving the last dose of the study vaccine. All volunteers will be strongly encouraged to use condoms throughout the duration of the study.

Appropriate interactive counselling will be provided to help volunteers determine the most appropriate form of family planning according to individual needs.

8.6 Randomisation, blinding and unblinding

Volunteers will be identified by a unique study identification number which will be assigned upon commencement of screening.

Volunteers will be randomised according to the randomisation schedule prepared by the statisticians at the Data Coordinating Centre (DCC) prior to the start of the study. Volunteers will be automatically assigned a unique allocation number as they are enrolled into the data entry system, corresponding to an allocation number on the unblinding list that will be provided to the unblinded site pharmacist by the DCC.

This is a double-blind study. Study staff (investigator and clinical personnel monitoring the safety and laboratory assay results) and volunteers will be blinded with respect to the allocation of Investigational Medicinal Product (vaccine or placebo).

A volunteer will be considered enrolled once he/she has been assigned an allocation number.

Volunteers will be informed about their assignment (vaccine/placebo) at study completion, once the database is locked. Should a study volunteer be unblinded during the study, further administration of the Investigational Medicinal Product (vaccine or placebo) will be discontinued. The study volunteer will be followed up until the end of the study according to Schedule of Procedures (Appendix A).

8.7 Un-blinding Procedure for Individual Volunteer

When indicated (in the event of a medical emergency where the study physician believes the management/medical treatment of the volunteer would be altered by the knowledge of the group assignment), an individual volunteer can be un-blinded by the principal investigator after consultation with the study medical monitor. The procedures and contact numbers for un-blinding are outlined in the SOM.

The un-blinded information should be restricted to a small group of individuals involved in clinical management/medical treatment of the volunteer (e.g., treating physician) and the blind must be maintained for those responsible for the study assessments.

The reasons for un-blinding should be documented and the sponsor, the medical monitor and the DCC should be notified as soon as possible.

In the event that *accidental* un-blinding of an individual volunteer occurs, the P.I. or study physician must report the occurrence promptly to the sponsor. All details surrounding the accidental un-blinding must be documented.

8.8 Sample Handling

Approximately 650 ml of blood from volunteers will be drawn over a period of 12 months. The study site will adhere to the Laboratory Analytical Plan (AP), the study-specific procedures manual/operations manual, and both local and sponsor SOPs for proper collection, processing, labeling, transport, and storage

of specimens. Specimen collection, testing, and storage at the laboratory will also be documented in compliance with Good Clinical Laboratory Practices.

In the event of an abnormal laboratory value, volunteers may be asked to have an additional sample collected at the discretion of the Principal Investigator or designee.

9 INVESTIGATIONAL MEDICINAL PRODUCT (IMP)

9.1 IMP Description

There are three investigational medicinal products to be studied in HIV-CORE 006 and one placebo. All investigational medicinal products were manufactured under Good Manufacturing Practices and in compliance with European Union GMP guidelines, Annex 13. Prior to IM injection the vaccines will be prepared by a trained and unblinded pharmacy member who will have no contact with the clinic or volunteers. The prepared IMP syringes will be masked to maintain the blind.

Two of the IMPs are MVA-based vaccines called MVA.tHIVconsv3 and MVA.tHIVconsv4. Both were manufactured, labeled and technically released by IDT Biologika GmbH in Dessau-Rosslau, Germany. MVA is an efficient single-round expression vaccine vector that is itself incapable of replication and spread in mammals. Both MVA.tHIVconsv3 and MVA.tHIVconsv4 contain a transgene (insert) coding for 6 conserved HIV regions that are fused together to form a chimeric protein immunogen. These 6 regions are arranged in different unique orders.

MVA.tHIVconsv4 and MVA.tHIVconsv3 vaccines are presented as white cloudy solutions, formulated in Tris-saline buffer (10 mM Tris HCl, 140 mM NaCl, pH 7.7) at a concentration of 1.8×10^8 pfu/ml for MVA.tHIVconsv4 and 3.4×10^8 pfu/ml for MVA.tHIVconsv3. The extractable fill volume is 500 µl. The products are supplied in sterile rubber-stopped glass vials. The vials are stored frozen below -65 °C.

The other IMP is a Chimpanzee Adenovirus-based vaccine called ChAdOx1.tHIVconsv1. It was manufactured, labeled and technically released by Advent S.r.l. in Rome, Italy. The ChAdOx1 vaccine vector is an engineered, non-replicating vector derived from simian adenovirus. ChAdOx1.tHIVconsv1 contains a transgene (insert) coding for 6 conserved HIV regions that are fused together to form chimeric protein immunogen.

The ChAdOx1.tHIVconsv1 vaccines are supplied in standard glass vials with rubber stoppers and caps. It is a slightly opaque frozen liquid supplied at a target concentration of $>1.1 \times 10^{11}$ vp/ml. The fill volume per vial is 0.65 ml. The vials are stored frozen below -65 ˚C.

All the vaccines will be labeled according to EU requirements for primary and secondary labels.

The placebo to be used in the study is normal saline and can be stored at 2-8 °C or at room temperature (25 °C ± 5 °C).

A description of all of the Investigational Medicinal Products is summarized in Table 9.1-1.

Table 9.1-1 Description of Investigational Medicinal Products

Vaccine/Placebo	Formulation	Concentration	Dosage	Total Volume in Vial (ml)	Total Volume to be injected (ml)	Route of Admin.
MVA.tHIVconsv3	10 mM Tris-HCl, 140 mM NaCl, pH 7.7	3.4×10^8 pfu/mL	1.0×10^8 pfu	0.52 ml	0.3 ml	IM
MVA.tHIVconsv4	10 mM Tris-HCl, 140 mM NaCl, pH 7.7	1.8×10^8 pfu/mL	0.9×10^8 pfu	0.5 ml	0.5 ml	IM
ChAdOx1.tHIVconsv1	10 mM L-Histidine, 35 mM Sodium Chloride, 1 mM Magnesium Chloride, 0.1% (w/v) PS80, 7.5% (w/v) Sucrose, 0.5% (v/v) Ethanol and 0.1 mM EDTA.	1.3×10^{11} vp / ml	5.0×10^{10} vp	0.65 ml	0.4 ml	IM
Placebo	0.9% Sodium Chloride	TBD		1.0 ml	0.3-0.5 ml	IM

IM = Intramuscular

9.2 Shipment and Storage of IMP

The MVA.tHIVconsv3, MVA.tHIVconsv4 and ChAdOx1.tHIVconsv1 investigational medicinal products are stored frozen below -65 °C. Normal saline for the placebo can be stored at 2-8 °C or at room temperature (25 °C ± 5 °C).

Authorisation to ship the Investigational Medicinal Product to the clinical site will be provided in writing by the Sponsor, upon confirmation that all required critical documents for shipment authorisation have been completed. The MVA.tHIVconsv3, MVA.tHIVconsv4 and ChAdOx1.tHIVconsv.1 vaccine candidates will be shipped on dry ice with a temperature monitoring device included inside the shipping container. Placebo can be shipped at ambient temperature.

Upon receipt at the pharmacy, the IMPs are inspected and then stored in a secure location in the pharmacy at the proper temperature. IMPs must be stored under continuous temperature monitoring for the duration of the clinical study.

9.3 Preparation of Investigational Medicinal Product (IMP)

Detailed instructions are provided in the SOM for preparing each of the investigational medicinal products. Volunteers will be randomised to receive either the investigational medicinal product or placebo. The pharmacist will be unblinded, but the study physician or designee administering the vaccine, clinical and laboratory staff, and PI will all be blinded. The dosage volumes to be administered are listed in Table 9.1-1. Instructions for reconciliation of used vials and subsequent disposal will be provided in the SOM. Syringes or other components that come in direct contact with Investigational Medicinal Products will be disposed of in a biohazard container and incinerated or autoclaved

9.4 Administration of Investigational Medicinal Product

IMP will be administered by intramuscular injection at the timepoints specified in the Schedule of Procedures (Appendix A).

Volunteers will receive two IM injections with half dose of ChAdOx1.tHIVconsv1 into each arm at enrollment, and two IM injections (MVA.tHIVconsv3 into one arm and MVA.tHIVconsv4 into the other arm) at the second vaccination visit.

The preferred site of administration is the deltoid muscle, unless contraindicated for any reason.

Complete instructions for administration of the Investigational Medicinal Product are supplied in the Study Operations Manual.

9.5 Accountability and Disposal of the Investigational Medicinal Product

A verifier will be required to double check and document each step of IMP preparation. All used vials will be returned to the IMP dispenser or pharmacy at the end of each vaccination visit. Accountability of the IMP will be maintained as follows:

- The date, vial allocation number and location of storage of the returned vials will be recorded.
- During the study, the investigational medicinal product accountability forms including dispensing and returning of vials will be kept and monitored.
- Accountability logs will be kept and monitored for each IMP for the duration of the study, to track vials received and dispensed.
- Used, empty vials will either be retained by the pharmacy until reconciliation by the monitor or the vial labels will be removed from used IMP vials and affixed to pharmacy documentation and the empty vials will be discarded as per site procedures.
- At the end of the study, the used and unused vials will be handled according to instructions of the Sponsor.
- Further information on Accountability and Disposal is supplied in the Study Operations Manual.

10 ASSESSMENTS

10.1 Safety Assessments

Data on local and systemic reactogenicity will be collected by structured interview and medical examination. Data on other adverse events will be collected with open-ended questions. All data will be recorded on the appropriate source documents and entered into the study database.

Signs and symptoms related to local and systemic reactogenicity events will be assessed by study staff prior to vaccination and at least 30 minutes post-vaccination as specified in the Schedule of Procedures (Appendix A).

Volunteers will be provided with and instructed on the use of Memory Aid to collect and grade reactogenicity events. Study staff will review the Memory Aid data with the volunteers before recording it in the volunteer source chart.

Data on other adverse events will be collected with open-ended questions. All data will be recorded on the appropriate source documents and entered into the study database.

10.1.1 Local reactogenicity

The presence of local reactogenicity will be assessed at the time points specified in the Schedule of Procedures (Appendix A).

Pain, tenderness, erythema/skin discoloration, swelling/hardening or thickening will be assessed and graded using Appendix C, Adverse Event Severity Assessment Table, as a guideline.

10.1.2 Systemic reactogenicity

The presence of systemic reactogenicity will be assessed at the time points specified in the Schedule of Procedures (Appendix A).

Fever, chills, headache, nausea, vomiting, malaise, sweating, fatigue, myalgia, and arthralgia will be assessed and graded using the Appendix C, Adverse Event Severity Assessment Table as a guideline.

10.1.3 Unsolicited Adverse Events

Unsolicited adverse events (AEs) will be collected through 28 days post final vaccination. Serious Adverse Events (SAEs) will be collected throughout the entire study period.

Open ended questions will be asked at time points according to the Schedule of Procedures (Appendix A). All adverse events will be graded using Appendix C, Adverse Event Severity Assessment Table, as a guideline and will be assessed for causality to the IMP. For more information regarding adverse events refer to Section 11, Safety Reporting.

10.1.4 Assessment of Lymph Nodes

At each vaccination visit, an assessment of cervical and axillary lymph nodes is performed by study staff prior to vaccination, and at reactogenicity visits as specified in Schedule of Procedures (Appendix A). If lymphadenopathy is increased relative to baseline, it will be followed until resolution.

10.1.5 Vital Signs

At each vaccination visit, vital signs (pulse, respiratory rate, blood pressure and temperature) will be measured by study staff prior to vaccination and at least 30 minutes post-vaccination.

For the other study visits, vital signs will be assessed at the time points specified in the Schedule of Procedures (Appendix A).

10.1.6 Concomitant Medications

Concomitant receipt of Investigational Medicinal Products, including other HIV vaccines, is prohibited during the study. Contraceptive use and significant medication ongoing at study entry will be documented.

During the study, information regarding concomitant medications and reasons for their use will be solicited from the study volunteers from enrolment through 28 days post final vaccination. Ongoing concomitant medications will be recorded until the end of the study.

10.1.7 Routine laboratory parameters

Table 10.1.7-1 shows the laboratory parameters that will be measured routinely. The samples for these tests will be collected at the time points indicated in the Schedule of Procedures (Appendix A).

Table 10.1.7-1: Laboratory Parameters

**Laboratory Parameter**	**Test**
Haematology	Haemoglobin, haematocrit, leukocytes, platelets, absolute neutrophil count (ANC), absolute lymphocyte count (ALC)
Clinical Chemistry	Liver function tests: aspartate aminotransferase (AST), alanine aminotransferase (ALT), total and direct bilirubin.<br><br>Kidney function: creatinine
Urinalysis	Dipstick test for protein, blood glucose, ketones, esterase (leukocytes) and nitrite. If clinically significant abnormalities (e.g.

	blood, protein, leukocytes) are found on dipstick test, then further test(s) will be performed (e.g. microscopy, culture).

10.1.8 Specific screening tests

Volunteers will be screened to exclude the following diseases:

- Hepatitis B: positive for hepatitis B surface antigen (HBsAg)
- Hepatitis C: positive for hepatitis C virus antibodies (HCV antibodies)
- Active syphilis: confirmed diagnosis (e.g., positive RPR confirmed by TPHA)

10.2 Immunogenicity Assessments

10.2.1 Cellular Responses

T cell responses will be determined initially by an Ex Vivo IFN-γ ELISPOT assay using a panel of overlapping peptides covering the length of the immunogen. Induction of T cells that can inhibit replication in vitro of viruses of major HIV-1 clades A, B, C and D will be assessed by a viral inhibition assay using a panel of infectious molecular clones representative of circulating viruses at the study site and other global clades. T cell polyfunctionality and the induction of immunological memory and homing patterns of T cells will be examined by intracellular cytokine staining by flow cytometry. Detailed epitope mapping studies are also planned again using flow cytometry to more fully examine the depth of vaccine induced T cell responses.

10.2.2 Antibody Responses

The induction of antibody will be measured by a standard endpoint ELISA assay using peptides covering the immunogen.

10.2.3 PBMC, Serum and Plasma Storage

PBMC, serum and plasma samples will be stored as indicated in the Laboratory Analytical Plan (LAP) and analysed for the purposes outlined within this protocol. Consent will be sought for long-term storage, for up to 15 years, of research samples for possible use in future HIV or other disease research. Samples not consented for long-term storage are to be stored until study close out and thereafter disposed of following the local country requirements and regulations relating to the disposal of biological research samples.

10.3 Other Assessments

10.3.1 HLA Typing

Samples for HLA typing will be collected as specified in the Laboratory Analytical Plan and may be analysed as warranted.

10.3.2 Microbiome Testing

Characterization of the gut microbiome composition and richness will be done using a shotgun sequencing and we may also perform extensive metabolomic analyses, which show associations with microbial dysbiosis. Volunteers will be given the option to provide faecal samples which will be collected at the timepoints indicated in the Schedule of Procedures (Appendix A).

10.3.3 HIV testing

Samples will be tested at the time points indicated in the Schedule of Procedures (Appendix A). Further information is specified in Section 12.1 HIV Testing

10.3.4 Pregnancy Test

A urine pregnancy test for all female volunteers will be performed by measurement of Human Chorionic Gonadotrophin (βhCG) at time points indicated in the Schedule of Procedures (Appendix A). The results of the pregnancy test must be negative prior to each vaccination.

10.3.5 HIV Risk Assessment

Study staff will assess volunteers for their past and current risk of acquiring HIV at time points indicated in Schedule of Procedures (Appendix A).

10.3.6 Social Impact Assessment

Each volunteer will have a social impact assessment administered at the time point specified in the Schedule of Procedures (Appendix A). This assessment is intended to assess the impact of trial participation on the volunteer’s personal and professional life.

11 SAFETY REPORTING

11.1 Definitions

Adverse Event (AE)	Any untoward medical occurrence in a volunteer to whom a medicinal product has been administered, including occurrences which are not necessarily caused by or related to that product.
Adverse Reaction (AR)	An untoward and unintended response in a volunteer to an investigational medicinal product which is related to any dose administered to that volunteer.<br>The phrase "response to an investigational medicinal product" means that a causal relationship between a trial medication and an AE is at least a reasonable possibility, i.e. the relationship cannot be ruled out.<br>All cases judged by either the reporting medically qualified professional or the Sponsor as having a reasonable suspected causal relationship to the trial medication qualify as adverse reactions.
Serious Adverse Event (SAE)	A serious adverse event is any untoward medical occurrence that:<br>• results in death<br>• is life-threatening<br>• requires inpatient hospitalisation or prolongation of existing hospitalisation<br>• results in persistent or significant disability/incapacity<br>• consists of a congenital anomaly or birth defect.<br>Other 'important medical events' may also be considered serious if they jeopardise the volunteer or require an intervention to prevent one of the above consequences.<br>NOTE: The term "life-threatening" in the definition of "serious" refers to an event in which the volunteer was at risk of death at the time of the event; it does not refer to an event which hypothetically might have caused death if it were more severe.
Serious Adverse Reaction (SAR)	An adverse event that is both serious and, in the opinion of the reporting Investigator, believed with reasonable probability to be due to one of the investigational medicinal products, based on the information provided.
Suspected Unexpected Serious Adverse Reaction (SUSAR)	A serious adverse reaction, the nature and severity of which is not consistent with the information about the medicinal product in question set out:<br>• in the case of a product with a marketing authorisation, in the summary of product characteristics (SmPC) for that product

	• in the case of any other investigational medicinal product, in the investigator's brochure (IB) relating to the trial in question.

NB: to avoid confusion or misunderstanding of the difference between the terms "serious" and "severe", the following note of clarification is provided: "Severe" is often used to describe intensity of a specific event, which may be of relatively minor medical significance. "Seriousness" is the regulatory definition supplied above.

For any pregnancy occurring during the clinical trial, the outcome of the pregnancy should be recorded and the infant followed up for congenital abnormalities or birth defects; should any abnormalities be detected, they would fall within the definition of "serious".

11.2 Causality

Assessment of relationship of an AE or SAE to IMP is the responsibility of the Principal Investigator or designee. All medically indicated and available diagnostic methods (e.g., laboratory, blood smear, culture, X-ray, etc.) should be used to assess the nature and cause of the AE/SAE. Best clinical and scientific judgment should be used to assess relationship of AE/SAEs to the IP and/or other cause.

The following should be considered:

- Presence/absence of a clear temporal (time) sequence between administration of the Investigational Medicinal Product and the onset of AE/SAE
- Presence/absence of another cause that could more likely explain the AE/SAE (concurrent disease, concomitant medication, environmental or toxic factors)
- Whether or not the AE/SAE follows a known response pattern associated with the Investigational Medicinal Product

The relationship assessment should be reported as one of the following:

Not Related: clearly explained by another cause (concurrent disease, concomitant medication, environmental or toxic factors, etc.).

Unlikely: more likely explained by another cause (concurrent disease, concomitant medication, environmental or toxic factors, etc.).

Possibly: equally likely explained by another cause but the possibility of the Investigational Medicinal Product relationship cannot be ruled out (e.g., reasonably well temporally related and/or follows a known Investigational Medicinal Product response pattern but equally well explained by another cause).

Probably: more likely explained by the Investigational Medicinal Product (e.g., reasonably well temporally related and/or follows a known Investigational Medicinal Product response pattern and less likely explained by another cause).

Definitely: clearly related and most likely explained by the Investigational Medicinal Product.

For the purpose of expedited safety reporting, all possibly, probably or definitely related AEs are considered Investigational Medicinal Product-related AEs.

11.3 Assessment of Severity of Adverse Events

The severity of events will be assessed on the following scale: Grade 1 = mild, Grade 2 = moderate, Grade 3 = severe, Grade 4 = very severe (potentially life threatening).

The following general criteria should be used in assessing adverse events as mild, moderate, severe or very severe at the time of evaluation:

Grade 1 (Mild): Symptoms causing no or minimal interference with usual social & functional activities

Grade 2 (Moderate): Symptoms causing greater than minimal interference with usual social & functional activities

Grade 3 (Severe): Symptoms causing inability to perform usual social & functional activities

Grade 4 (Very severe/Potentially Life Threatening): Symptoms causing inability to perform basic self-care functions OR Medical or operative intervention indicated to prevent permanent impairment, persistent disability, or death

Guidelines for assessing the severity of specific adverse events and laboratory abnormalities are listed in Appendix C, Adverse Event Severity Assessment Table.

AEs considered related to the trial medication as judged by a medically qualified investigator or the Sponsor will be followed either until resolution, or the event is considered stable.

It will be left to the Investigator's clinical judgment to decide if an AE is of sufficient severity to require the volunteer's discontinuation from further vaccination. A volunteer may also voluntarily withdraw from further vaccination due to what he or she perceives as an intolerable AE. If either of these occurs, the volunteer must be given appropriate care under medical supervision until symptoms cease, or the condition becomes stable.

11.4 Procedures for Recording Adverse Events

All AEs occurring from enrolment through 28 days post final vaccination that are observed by the Investigator or reported by the volunteer, will be recorded on the CRF, whether or not attributed to trial vaccination.

The following information will be recorded: description, date of onset and end date, severity, whether or not it is a serious adverse event, assessment of relatedness to investigational medicinal products and whether treatment was required. Follow-up information should be provided as necessary.

11.5 Reporting Procedures for Serious Adverse Events

Serious Adverse Events (SAEs) should be reported to the sponsor/designee within 24 hours of the site becoming aware of the event, and sent to the Sponsor as described in the SOM.

To discuss Investigational Medicinal Product-related SAEs or any urgent medical questions related to the SAE, the site investigator should contact one of the sponsor/designee directly (see Contact List in the SOM).

The SAE Report Form should be completed with all the available information at the time of reporting and sent to the Sponsor as described in the SOM. The minimum data required in reporting an SAE are the study identification number, date of birth, gender, event description (in as much detail as is known at the time), onset date of event (if available), reason event is classified as serious, reporting source (name of Principal Investigator or designee), and relationship to the Investigational Medicinal Product as assessed by the investigator.

The Data Monitoring and Ethics Committee (DMEC) will also be informed by the local PI of SAEs immediately or within 24 hours of awareness. The DMEC will review the SAE and confirm agreement with the investigator's assessment of relationship to vaccine and whether any specific safety measures are required (e.g. whether to delay or halt vaccinations). The DMEC will respond either via email or by teleconference as necessary.

The Principal Investigator or designee must notify the local REC of all SAEs as appropriate. In case of Investigational Medicinal Product-related SAEs, the Sponsor will notify responsible regulatory authorities, and other study sites where the same Investigational Medicinal Product is being tested.

The Principal Investigator or designee is required to prepare a detailed written report with follow up until resolution or until it is judged by the Principal Investigator or designee to have stabilized.

More details on SAE definitions and reporting requirements are provided in the SOM.

11.6 Expectedness

Expectedness will be determined according to the Investigator's Brochure.

11.7 SUSAR Reporting

All SUSARs will be reported by the Principal investigator to the relevant Regulatory Authority and to the Ethics Committees and other parties as applicable. For fatal and life-threatening SUSARS, this will be done no later than seven calendar days after the Sponsor or delegate is first aware of the reaction or following timelines required in the individual countries, whichever one is more stringent. Any additional relevant information will be reported within 8 calendar days of the initial report. All other SUSARs will be reported within 15 calendar days.

Allocation codes will be un-blinded for specific volunteers.

Principal Investigators will be informed of all SUSARs for all studies with the same IMP, whether or not the event occurred in the current trial.

11.8 Clinical Management of Adverse Events

The clinical study team will manage adverse events (AEs). They will assess volunteers, provide first line of care as appropriate and refer to health care and treatment facilities as warranted. If any

treatment/medical care is required as a result of the harm caused by the Investigational Medicinal Product or study procedures, this will be provided free of charge.

If a volunteer has an AE and/or abnormal laboratory value that is known at the time of study vaccination, the specifications of Section 13.0 will be followed.

Volunteers will be followed until the AE resolves or stabilizes or up to the end of the study, whichever comes last. If at the end of the study, an AE (including clinically significant laboratory abnormality) that is considered possibly, probably or definitely related to the Investigational Medicinal Product is unresolved, follow-up will continue until resolution if possible and/or the volunteer will be referred.

11.9 Pregnancy

Although not considered an AE, if a female volunteer becomes pregnant during the study, it is the responsibility of the Principal Investigator or designee to report the pregnancy promptly to the sponsor/designee using the designated forms. Vaccinations will be discontinued and the volunteer followed for safety until the end of pregnancy or study completion, whichever occurs last. If possible, approximately 2–4 weeks after delivery, the baby will be examined by a physician to assess its health status and the results will be reported to the sponsor/designee. The baby will be examined again by a physician around the age of 1 year and the results will be reported to the sponsor/designee.

Complications of pregnancy that meet criteria for a serious adverse event, specified in Section 11.1 of this Protocol (e.g., hospitalization for eclampsia, spontaneous abortion, etc.) should be reported as SAEs.

11.10 Intercurrent HIV Infection

The Investigational Medicinal Products cannot cause HIV infection. If a volunteer acquires HIV through exposure in the community, study vaccinations must be discontinued and the volunteer should have an Early Termination (ET) visit and offered referral to appropriate care and treatment facilities.

Intercurrent HIV infection in study volunteers, although not considered an SAE, must be reported promptly to the sponsor/designee using the designated forms. However, medical conditions associated with the HIV infection that meet criteria for serious specified in the Section 11.1 of this Protocol (e.g., sepsis, Pneumocystis jiroveci [carinii] pneumonia, etc.) should be reported as SAEs using the SAE Report Form.

11.11 SAFETY OVERSIGHT

11.11.1 Protocol Safety Review Team (PSRT)

A PSRT will be formed to monitor the clinical safety data. During the vaccination phase of the trial, the PSRT will review the clinical safety data on a weekly basis via electronic distribution of reports. An ad hoc PSRT review meeting will occur if any of the members of the PSRT requests a special review to discuss a specific safety issue or as specified in the Study Operations Manual. After the vaccination phase the PSRT will review the clinical safety data every 2 weeks.

The PSRT will consist of the Chief Investigator or designee, the IAVI Medical Monitor and PIs or designees from each clinical team. The study Chief Investigator or a sponsor/designee Medical Monitor may be the PSRT chair. Ex officio members will include representatives from the DCC.

Additional PSRT participants may include the following, as needed:

- Co-investigators and trial site senior clinical research nursing staff
- Laboratory directors
- Data management, study statistician and regulatory staff

The PSRT membership and procedures are detailed in the PSRT charter.

11.11.2 Data Monitoring and Ethics Committee (DMEC)

The DMEC will oversee the progress of the study. The DMEC will consist of independent clinicians/scientists/ethicists who are not involved in the study. Investigators responsible for the clinical care of volunteers or representatives of the Sponsor will not be members of the DMEC. Details of membership, chair and co-chair and responsibilities are outlined in the DMEC charter.

Principal Investigator(s) or designee and/or a Sponsor representative may be asked to join an open session of the DMEC meeting to provide information on study conduct, present data or to respond to questions.

Safety data will be reviewed by the DMEC at pre-specified time points and on an ad-hoc basis. The DMEC will review data from the first 10 volunteers at 2 weeks after vaccination with the prime and again at 2 weeks after 10 volunteers have received the boost.

11.11.3 Content of Interim Safety Review

The DMEC will be asked to review the following blinded data:

- Summary of reactogenicity (i.e., solicited adverse events)
- All clinical adverse events judged by the Principal Investigator or designee to be possibly, probably or definitely related to the Investigational Medicinal Product

- All laboratory adverse events confirmed on retest and judged by the Principal Investigator or designee to be possibly, probably, or definitely related to Investigational Medicinal Product and/or clinically significant

- All SAEs

- Any other situation where the PI, CI or Sponsor thinks independent advice or review is important.

An un-blinded presentation of all above-noted events may also be made available for the DMEC for their review if required by any member of the DMEC.

11.11.4 Criteria for Pausing the Study

Enrolment and vaccinations will be stopped, and a safety review conducted by the DMEC, for any of the following criteria:

1. One or more volunteers experiences a Serious Adverse Event that is judged possibly, probably or definitely related to the IMP.
2. There is a volunteer death, regardless of relationship to the IMP.
3. If two or more volunteers experience grade 3 adverse events (including reactogenicity events) in the same System Organ Class that are considered to be at least possibly related to IMP
4. Any grade 4 adverse event that is considered to be possibly, probably, or definitely related to IMP

The study will continue enrollment and vaccinations only after evaluation of relevant safety data by the DMEC.

The Sponsor will request a review by the DMEC, (or the DMEC chair if other DMEC members cannot be convened), to be held within two business days of the Sponsor learning of the event. The individual volunteer(s)/or study may be unblinded at the discretion of the DMEC.

Following this review, the DMEC will make a recommendation regarding the continuation or suspension of the vaccinations or the trial and communicate this decision immediately to the Sponsor. The Sponsor then will inform the Principal Investigators without delay.

Additional ad hoc review may be specifically requested by the Sponsor, the Principal Investigator(s) or by the DMEC.

12 MANAGEMENT OF HIV ISSUES DURING AND FOLLOWING STUDY

12.1 HIV Testing

All volunteers will be tested for HIV antibodies as indicated in the Schedule of Procedures (Appendix A) or as needed, if medical or social circumstances arise. All volunteers will receive HIV risk reduction counselling and pre- and post-HIV-test counselling, as specified in Section 12.3.1 Counselling.

Volunteers who have a positive HIV-antibody test as a result of vaccine-induced HIV antibodies will have their test results reported as “Not infected with HIV-1 or HIV-2” (to prevent unblinding of volunteer and

staff). A vaccine recipient who still tests HIV positive due to vaccine-induced antibodies (VISP) at the end of the study will be informed of his/her positive test result and offered continuing follow-up until the test becomes negative.

If a volunteer is found to be HIV-infected through exposure to HIV in the community during the course of the study, study vaccinations must be discontinued and the volunteer will be followed up as specified in Section 12.3.

Should a volunteer require HIV testing outside the study for personal reasons, it is recommended that the volunteer contact the study staff first. HIV testing can be done at the study site and then processed at an independent laboratory as above. Written evidence of HIV status (HIV-infected or HIV-uninfected) will be provided upon request.

12.2 Social Discrimination as a Result of an Antibody Response to Vaccine

In order to minimize the possibility of social discrimination among volunteers (if any) who develop vaccine-induced HIV antibodies and test positive on a diagnostic HIV antibody test, appropriate diagnostic HIV testing and certification will be provided both during and after the study as needed.

12.3 HIV Infection

Volunteers who are found HIV infected at screening and volunteers who acquire HIV infection after screening, during the study (intercurrent HIV-infection) will be provided the following:

12.3.1 Counselling

The volunteer will be counselled by the study counsellors. The counselling process will assist the volunteer with the following issues:

- Psychological and social implications of HIV infection
- Who to inform and what to say
- Implications for sexual partners
- Implications for child-bearing
- Avoidance of transmission to others in future

12.3.2 Referral for Support /Care

Volunteers will be referred to a patient support centre or institution of his/her choice for a full discussion of the clinical aspects of HIV infection. Referral will be made to a designated physician or centre for discussion of options of treatment of HIV-infection.

For those individuals who become HIV infected after enrolment in the study (i.e., from first vaccination through final study visit), antiretroviral therapy will be provided when clinically indicated according to nationally accepted treatment guidelines.

Volunteers with confirmed HIV infection should have an early termination visit. In the unlikely event that HIV infection is confirmed during a reactogenicity period, the Medical Monitor should be contacted to determine the course of action on a case-by case-basis.

HIV-infected pregnant women will be referred for prenatal care and to a program for the Prevention of Mother to Child Transmission (PMTCT) as per site-specific procedures. The pregnant volunteer will be followed according to timelines specified in Section 11.8

13 DISCONTINUATION OF VACCINATIONS AND/OR WITHDRAWAL FROM STUDY

13.1 Discontinuations of Vaccinations

Any planned or unplanned discontinuation from further vaccinations will be discussed with the sponsor/designee. Volunteers may be discontinued from further vaccination for any of the following reasons:

1. Pregnancy
2. Intercurrent HIV Infection
3. Use of systemic corticosteroids, immunosuppressive, anticancer, anti-tuberculosis or other medications considered significant by the investigator. Note: Volunteers requiring chronic corticosteroids (> 2 weeks) or long term therapy will not receive any further vaccinations
4. A disease or condition or an adverse event that may develop, regardless of relationship to the Investigational Medicinal Product, if the Principal Investigator or designee is of the opinion that further study vaccinations will jeopardize the safety of the volunteer
5. Any immediate hypersensitivity reaction judged to be related to the IMP, e.g., anaphylaxis and related symptoms such as hives, respiratory difficulty, angioedema and/or abdominal pain
6. Life threatening adverse event following study vaccinations unless not related to the Investigational Medicinal Product
7. Volunteer's request to discontinue further vaccination
8. A grade 4 reactogenicity event
9. A grade 3 reactogenicity event that persists at the grade 3 level longer than 72 hours
10. For grade 3 or greater unsolicited adverse event(s) possibly, probably, or definitely related to the vaccine administrations, evaluation will be on a case-by-case basis with particular emphasis on duration of signs and symptoms and/or any evolving pattern. The site Principal Investigator, and medical monitor will review the adverse event and consult with the Protocol Safety Review Team members prior to a final decision on discontinuation of the volunteer from subsequent vaccination in the trial.

All volunteers discontinued from subsequent vaccination will be followed for safety, at a minimum, until resolution or stabilization of all adverse events or reactions occurring during the follow-up period

The following events require resolution and/or review of clinical history by the Principal Investigator or designee and consultation with the Medical Monitor, prior to continuation of study vaccination(s):

1. Any of the following abnormal laboratory values that are known at the time of vaccination and have not resolved:

   Haematology

   - Haemoglobin: <9.5 g/dl in females; <11.0 g/dl in males
   - Absolute Neutrophil Count (ANC) – all volunteers: ≤1,000/mm^3
   - Absolute Lymphocyte Count (ALC) – all volunteers: ≤650/mm^3
   - Platelets: <100,000 cells/mm^3

   Chemistry

   – Creatinine >1.1 x upper limit of normal (ULN)
   – AST >1.25 x ULN
   – ALT >1.25 x ULN

   Urinalysis: Dipstick 3+ confirmed by microscopy for

   - Protein
   - Blood (not due to menses)

   All above-noted events should be confirmed on the original sample and/or repeated at least once to confirm abnormal values.

2. Receipt of vaccines (non-HIV) within 28 days or immunoglobulin within 3 months of administration of the Investigational Medicinal Product.

3. A Grade 3 or 4 local reactogenicity event involving the major part of the injected arm circumference.

4. If the use of a short tapering course (<2 weeks) of corticosteroids is required, the study vaccinations may be continued after a 4-week washout period, provided that the medical condition requiring this therapy has completely resolved and in the opinion of both the site investigator and Medical Monitor, the continuation of the study vaccinations will not jeopardize the safety of the volunteer.

5. Participating in another clinical study of an Investigational Medicinal Product

13.2 Deferral of Vaccinations

Vaccinations may be temporarily deferred if the volunteer is clinically ill at the time of the vaccination visit and/or presents with fever at the time of the vaccination visit. A volunteer must be clinically well and afebrile for a minimum of a 24-hour consecutive period prior to vaccination.

13.3 Follow up after Discontinuation of Further Vaccinations

Volunteers, who have study vaccinations discontinued due to adverse events, will be followed until the adverse event resolves or stabilizes or up to the end of the study, whichever comes last. These volunteers will not be replaced.

13.4 Withdrawal from the Study (Early Termination)

Volunteers may be withdrawn from the study permanently for the following reasons:

1. Volunteers may withdraw from the study at any time if they wish, for any reason
2. The Principal Investigator or designee has reason to believe that the volunteer is not complying with the protocol
3. If the sponsor or local regulatory authority decides to terminate or suspend the study
4. If a volunteer becomes infected with HIV (through exposure in the community)

If a volunteer withdraws or is withdrawn from the study, all termination visit procedures will be performed according to the Schedule of Procedures (Appendix A) where possible. Every effort will be made to determine and document the reason for withdrawal.

14 STATISTICS

A detailed statistical analysis plan will be developed by the DCC prior to database lock and final analysis.

14.1 Description of Statistical Methods

14.1.1 Analysis of demographics

Demographic characteristics (e.g., age at study vaccination in years, gender, ethnicity) will be summarized by group using descriptive statistics:

- Frequency tables will be generated for categorical variables such as gender.
- Mean, median, standard deviation will be provided for continuous data such as age.

14.1.2 Analysis of safety

Analyses will be performed on the safety population.

All analyses will be descriptive. Summaries will be presented by treatment group and dose, and by combined dose for placebos. Separate summaries will be presented for each site and for all sites combined.

The percentage of volunteers with at least one local solicited AE, with at least one systemic solicited AE (both within 7 days of vaccination) and with any unsolicited AE (within 28 days of vaccination), will be tabulated, with exact 95% CI, by maximum severity per volunteer. Unsolicited AEs will include the proportion of volunteers with any event of grade 3 or 4 severity.

The proportion of volunteers with vaccine related serious adverse events (SAEs) at any time during the study will be summarized.

Solicited local and systemic reactions will also be tabulated by each individual reaction.

The verbatim reports of unsolicited AEs will be coded according to MedDRA. The percentage of volunteers with at least one report of unsolicited AEs classified by MedDRA and reported up to 28 days after vaccination will be tabulated by systemic organ class (SOC), preferred term (PT) and severity, with exact 95% CI.

All unsolicited AE summaries will be repeated for those classed as possibly, probably or definitely related to study product.

At each haematology/biochemistry sampling time point, individual haematological and biochemical values will be presented as number and proportion of volunteers out of range (above or below normal range) and tabulated by toxicity grading (refer to Appendix C).

SAEs and other important medical events will be described in detail. Withdrawals due to AEs/SAEs will also be summarized or listed.

14.1.3 Analysis of immunogenicity

The study hypothesis is that mosaic immunogens delivered by a prime-boost regimen of non-replicating simian adenovirus followed by non-replicating poxvirus modified vaccinia virus Ankara (MVA) will induce $CD8^+$ T cells, specific for conserved epitopes common to HIV-1 variants, efficient in controlling HIV-1 infection or helping prevent establishment of chronic infection. Summary statistics will be mainly descriptive, though statistical tests of differences between the prime-boost and placebo groups, and within group tests of changes from baseline will be performed and p-values presented. This study is not powered to detect statistically significant differences between vaccine and placebo recipients in the magnitude or breadth of immune responses, however, it may be possible to discern trends, which could inform the design of future studies.

The analysis of immunogenicity will be performed primarily on the Per-Protocol set. Proportions will be reported as for safety (proportions and exact 95% CIs), but will include group comparisons based on the binomial distribution. All continuous outcomes will be analyzed on a $\log_{10}$ scale and back-transformed to the original scale for presentation, summarized by number of non-missing values, geometric mean (GM) with 95% CI, median (and 95% CI based on 1000 bootstraps) and range. Fold-rise from baseline will also be analyzed, based on post-baseline minus baseline values (log scale). Between-group comparisons will be analyzed by the Wilcoxon 2-sample test and within group changes from baseline will be analyzed by the signed-rank test.

14.2 The Number of Volunteers

The primary goal of this study is to evaluate the safety and tolerability of the vaccine regimen. The trial will enrol 88 volunteers and it is not the remit of this study to recruit sufficient numbers of volunteers to be statistically confident about the result. However, the incidence of serious adverse reactions will be used as a measure of the safety of a prime-boost regimen of non-replicating simian adenovirus followed by non-replicating poxvirus modified vaccinia virus Ankara (MVA).

The double-blind, randomised, placebo-controlled design of this study was chosen to minimise bias in the reporting of immunological data. Statistical analysis will be conducted to evaluate changes in $CD8^+$ T cells, specific for conserved epitopes common to HIV-1 variants, efficient in controlling HIV-1 infection or helping prevent establishment of chronic infection.

This study is not powered to detect a significant difference between the prime-boost regimen and placebo recipients in the magnitude or breadth of immune response to the immunogen. The sample size was chosen based on primary safety concerns, in particular the probability of detecting any SAEs which may be thought to be possibly, probably or definitely related to study product. Tables 2 and 3 show the sample size calculations for safety, which are expressed in terms of the ability to detect serious adverse reactions, i.e. the probabilities of observing 0 and 2+ (2 or more) related SAEs among a group of n=72 (any regimen) for a range of possible true event rates. For example, if none of the 72 volunteers receiving the vaccine experiences an SAE related to the vaccine, the 95% two-sided upper confidence bound for the rate of such reactions in a population exposed to the vaccines is 5%.

Note that every effort will be made to ensure that all randomised volunteers receive at least one IMP administration.

Table 2. Confidence in observed number of SAEs (n = 72)

Observed number of related SAEs	95% Confidence Interval (exact)
0	0.0 – 5.0%
1	0.04 – 7.5%
2	0.3 – 9.7%
3	0.9– 11.7%
4	1.5 – 13.6%
5	2.3 – 15.5%

Table 3. Probability of observing 0 and 2+ related SAEs at a given SAE rate (n = 72)

Event rate (Related SAEs)	Any regimen (n=72)	
	0 events	2+ events
0.010	0.48	0.16
0.025	0.16	0.54
0.035	0.08	0.72
0.050	0.025	0.88
0.100	<0.001	0.995
0.150	<0.001	>0.999
0.200	<0.001	>0.999
0.250	<0.001	>0.999

14.3 The Level of Statistical Significance

This study is not powered to detect statistically significant differences between groups or within groups. However, when reported, p-values <0.05 will be highlighted in the results.

14.4 Criteria for the Termination of the Trial

Termination of the trial can occur at any time at the discretion of the sponsor, based on their review of any events or advice from the DMEC that may occur as defined in section 11.11.4, Criteria for Pausing the Study.

14.5 Procedure for Accounting for Missing, Unused, and Spurious Data.

All attempts will be made to collect all data per protocol. As missing data are expected to be minimal, no imputation will be performed for missing values.

14.6 Analysis Populations

14.6.1 Safety Population

The safety population will include all volunteers with at least one vaccine/placebo administration documented.

14.6.2 Per Protocol Population (PP Population)

The PP population will be applied separately to each immunogenicity outcome and adapted by time point. To be eligible for the PP Population, the analysis time point must occur within the visit window and, up to the analysis time point, the volunteer must: 1) receive all scheduled doses per protocol, 2) have complete immunogenicity data, 3) have no major protocol deviations and 4) not receive any concomitant medications that potentially affect immunogenicity.

14.6.3 Unblinded Volunteers

If a volunteer is unblinded then any data collected after unblinding will be excluded from analysis.

14.7 Procedures for Reporting any Deviation(s) from the Original Statistical Plan

There are currently no deviations from the original statistical plan.

15 DATA MANAGEMENT

15.1 Source Data

Source documents are the documents where data are first recorded, and from which volunteers' CRF data are obtained. These include, but are not limited to, hospital records (from which medical history and previous and concurrent medication may be summarised into the CRF), clinical and office charts, laboratory and pharmacy records, diaries, microfiches, radiographs, and correspondence.

CRF entries will be considered source data if the CRF is the site of the original recording (e.g. there is no other written or electronic record of data). All documents will be stored safely in confidential conditions. On all trial-specific documents, other than the signed consent, the volunteer will be referred to by the trial volunteer number/code, not by name.

15.2 Access to Data

Direct access will be granted to authorised representatives from the Sponsor, host institution and the regulatory authorities to permit trial-related monitoring, audits and inspections.

15.3 Data Recording and Record Keeping

All trial data will be entered in the clinical trial database. Note that ICH GCP (Section 5.5) requires that electronic data entry systems are validated and that Standard Operating Procedures are maintained.

The volunteer will be identified by a unique trial specific number and/or code in any database. The name and any other identifying detail will NOT be included in any trial data electronic file.

15.4 DATA ENTRY AT STUDY SITE

The data collected at the site will be entered directly into the access -controlled clinical trial database, with a complete audit trail documenting all entries and changes made within the database. Some data, such as laboratory results, will be captured on source documents prior to entry into the clinical trial database. Source documents and CRFs may be used to capture clinical data in instances where access to the clinical trial database is unavailable due to connectivity issues. This is indicated on the Source Document Confirmation form. To provide for real time assessment of safety, data should be entered as soon as is reasonably feasible after a visit occurs keeping to the time limit specified in the SOM.

16 QUALITY ASSURANCE PROCEDURES

To ensure the quality and reliability of the data collected and generated and the ethical conduct of this study, a Study Operations Manual will be developed. All deviations will be reported and investigated. The Study Operations Manual describes reporting and deviation documentation requirements and procedures. Regular monitoring will be performed according to ICH-GCP as indicated in Section 17.2. An independent audit of the study and study sites may be performed by the Sponsor or designee to establish the status of applicable quality systems. Inspection by regulatory authorities may also occur. By signing the protocol, the Principal Investigators agree to facilitate study related monitoring, audits, IEC/IRB review and regulatory inspection(s) and direct access to source documents. Such information will be treated as strictly confidential and under no circumstances be made publicly available

17 DATA AND BIOLOGICAL MATERIAL

All data and biological material collected through the study shall be managed in accordance with the Clinical Trial Agreement (CTA). Distribution and use of these data will be conducted by agreement of all parties. The computerized raw data generated will be held by the DCC on behalf of the Sponsor. The study site will also hold the final data files and tables generated for the purpose of analysis. Principal investigators or designees will have access to the clinical study database with appropriate blinding.

18 ADMINISTRATIVE STRUCTURE

The Principal Investigator will be responsible for all aspects of the study at the study site

18.1 Study Supervision

The Principal Investigator will work closely with his/her study team to implement the study, address issues in a timely manner, assure consistent documentation, and compile and provide study progress reports to the Medical Monitor and DMEC. Accrual and retention rates, safety of study volunteers, and other relevant parameters will be regularly and closely monitored by the study team, Principal Investigator, Medical Monitor, and the DMEC.

18.2 Study Monitoring

Study monitoring will be conducted by IAVI. Monitoring will be conducted to ensure that: the rights and wellbeing of volunteers are protected; the reported data are accurate, complete and verifiable from source documents; and that study conduct complies with the currently approved protocol, standard operating procedures, Good Clinical Practice (GCP) and other applicable regulatory requirements.

Study monitors will regularly visit the study sites to review all trial documents including volunteer screening and enrolment logs, informed consent forms, source documents, CRFs, laboratory and medical records. The specific objectives of a monitoring visit will be to verify: 1) to verify the existence of adequately signed informed consent forms for each enrolled volunteer; 2) to verify the prompt, complete and accurate recording of data, and prompt reporting of all SAEs and SUSARs; 3) to verify the quality and accuracy of data by validation of CRFs against the source documents such as volunteers' medical records, laboratory reports, and any other relevant original data; 4) to verify adequate IMP supply, storage, management, and accountability; and 5) to ensure protection of study volunteers, and investigators' compliance with the protocol, regulatory requirements and applicable guidelines.

Study investigators and volunteers agree that the study monitor may review study facilities and source records and observe the performance of study procedures. Additionally, study investigators will permit inspection or audit of the study facilities and all study-related records by relevant regulatory authorities, the local IRB/IEC and/or representatives of the Sponsor. All information collected during monitoring or audit visits will be treated as strictly confidential and will under no circumstances be made publicly available.

18.3 Investigator's Records

Study records include administrative documentation, including reports and correspondence relating to the study, as well as documentation related to each volunteer screened for and/or enrolled in the study (e.g., screening and enrolment logs, locator information forms, informed consent forms, laboratory reports, case report forms, and all other source documents). The Principal Investigator will maintain and store, in a secure manner, complete, accurate, and current study records for a minimum of 5 years or according to local regulation (whichever longer) after the study is terminated and applicable regulatory authorities are notified. Study documents must not be destroyed without notifying the Sponsor.

19 SERIOUS BREACHES

A serious breach is defined as "A breach of GCP or the trial protocol which is likely to affect to a significant degree –

(a) the safety or physical or mental integrity of the volunteers in the trial; or

(b) the scientific value of the trial".

In the event that a serious breach is suspected, the Sponsor must be contacted within 1 working day. In collaboration with the PI, the serious breach will be reviewed by the Sponsor and, if appropriate, the Sponsor will ensure it is reported to the REC committee and relevant regulatory authorities within seven calendar days.

20 ETHICAL AND REGULATORY CONSIDERATIONS

20.1 Declaration of Helsinki Guidelines for Good Clinical Practice

The Principal Investigator will ensure that the study is conducted in compliance with the protocol, SOPs in accordance with guidelines formulated by the ICH for GCP in clinical studies, the ethical principles that have their origins in the Declaration of Helsinki and applicable local standards and regulatory requirements.

Deidentified volunteer-level study data may be shared with other researchers or made public in the future.

20.2 Approvals

The protocol, protocol amendments, site-specific informed consent documents, proposed recruitment materials, and other relevant documents will be reviewed and approved by the relevant local Research Ethics Committee (REC) and regulatory authorities. The trial will not be initiated before all the necessary approvals have been obtained. Amendments to the protocol will not be implemented without prior written REC approval except when they involve only logistical or administrative aspects of the study. The Principal Investigator will still have to submit such logistical or administrative amendments to REC.

The protocol, informed consent form, information sheet and any proposed advertising material will be submitted to an appropriate Research Ethics Committee (REC), regulatory authorities, and host institution(s) for written approval.

The Investigator will submit and, where necessary, obtain approval from the above parties for all substantial amendments to the original approved documents.

20.3 Reporting

The Principal Investigator shall submit once a year throughout the clinical trial, or on request, an Annual Progress Report to the REC, other regulatory authorities, and the Sponsor. In addition, an End of Trial notification and final report will be submitted to these institutions.

The Chief Investigator shall submit once a year throughout the clinical trial, or on request, an Annual Progress Report to the RECs, (including Oxford Tropical Medicine Ethics Committee (OxTREC)) and Sponsor. In addition, an End of Trial notification and final report will be submitted to the, the RECs, OxTREC and Sponsor.

20.4 Volunteer Confidentiality

The trial staff will ensure that the volunteers' anonymity is maintained. The volunteers will be identified only by a volunteer ID number on all laboratory specimens, trial documents and any electronic database. All documents will be stored securely and only accessible by trial staff and authorised personnel. Clinical information will not be released without written permission from the volunteer except as necessary for monitoring or auditing of the study.

20.5 Expenses and Benefits

Volunteers will be reimbursed for their time, effort and for costs to cover their travel expenses to the study site and any inconvenience due to study participation. Research centre-specific reimbursement amounts will be specified in the research centre-specific informed consent forms.

21 FINANCE AND INSURANCE

21.1 Funding

The study will be funded primarily by a grant from the European and Developing Countries Clinical Trials Partnership. The GMP manufacture of vaccines was funded by IAVI though the SOW 5, EDCTP through the GREAT award and EC H2020 though the EAVI 2020 award.

21.2 Indemnity

If any volunteer is harmed as a result of this trial, medical care will be provided. The sponsor should provide care until complete cure or stabilization of a research related injury. The injured research volunteer shall be given the best care available within the country for the research related injury. Research volunteers shall not be required to waive their legal rights for redress in courts of law.

Negligent Harm

Indemnity and/or compensation for negligent harm arising specifically from an accidental injury for which the University is legally liable as the Research Sponsor will be covered by the University of Oxford.

Non-Negligent Harm

Indemnity and/or compensation for harm arising specifically from an accidental injury, and occurring as a consequence of the Research volunteers' participation in the trial for which the University is the Research Sponsor will be covered by the University of Oxford.

21.3 Insurance

The University of Oxford has a specialist insurance policy in place which would operate in the event of any volunteer suffering harm as a result of their involvement in the research (Newline Underwriting Management Ltd, at Lloyd's of London and in Uganda specifically, a local insurance company, Jubilee Insurance Ltd.)

22 PUBLICATION POLICY

A primary manuscript and if appropriate ancillary manuscripts will be prepared promptly after data analysis is completed using mutually accepted Publication Guidelines. Authorship criteria will be based on contributions to the design, work, analysis and writing of the study report and authors may include investigators at the clinical research centres (CRCs) in Kenya, Uganda and Zambia, UOXF and IAVI. The trial results will be disseminated at organized meetings that will bring together stakeholders within the respective countries and will include at minimum, Government representatives from appropriate Ministries, research organizations, non-governmental organizations, the media, civil societies and

community representatives. A summary of the main findings will be developed that will be in non-technical language and translated into local language at the respective CRCs to be communicated to trial volunteers.

23 REFERENCES

1. UNAIDS. Fact sheet - Latest global and regional statistics on the status of the AIDS epidemic. *Unaids* [online]. 2017. No. June, p. 8. DOI 2017. Available from: http://www.unaids.org/sites/default/files/media_asset/UNAIDS_FactSheet_en.pdf

2. WORLD HEALTH ORGANIZATION. Access to anti-retroviral drugs in low and middle income countries. *World Health Organization* [online]. 2014. No. July, p. 1–37. Available from: http://apps.who.int/iris/bitstream/10665/128150/1/9789241507547_eng.pdf?ua=1&ua=1

3. OSTERBERG, Lars and BLASCHKE, Terrence. Adherence to Medication. *New England Journal of Medicine* [online]. 4 August 2005. Vol. 353, no. 5, p. 487–497. DOI 10.1056/NEJMra050100. Available from: http://www.nejm.org/doi/abs/10.1056/NEJMra050100

4. WEISER, John, BROOKS, John T., SKARBINSKI, Jacek, et al. Barriers to Universal Prescribing of Antiretroviral Therapy by HIV Care Providers in the United States, 2013–2014. *JAIDS Journal of Acquired Immune Deficiency Syndromes* [online]. April 2017. Vol. 74, no. 5, p. 479–487. DOI 10.1097/QAI.0000000000001276. Available from: http://insights.ovid.com/crossref?an=00126334-201704150-00001

5. SANOU, M. P., ROFF, S. R., MENNELLA, A., et al. Evolutionarily Conserved Epitopes on Human Immunodeficiency Virus Type 1 (HIV-1) and Feline Immunodeficiency Virus Reverse Transcriptases Detected by HIV-1-Infected Subjects. *Journal of Virology* [online]. 15 September 2013. Vol. 87, no. 18, p. 10004–10015. DOI 10.1128/JVI.00359-13. Available from: http://jvi.asm.org/cgi/doi/10.1128/JVI.00359-13

6. ANTROBUS, Richard D, COUGHLAN, Lynda, BERTHOUD, Tamara K, et al. Clinical Assessment of a Novel Recombinant Simian Adenovirus ChAdOx1 as a Vectored Vaccine Expressing Conserved Influenza A Antigens. *Molecular Therapy* [online]. March 2014. Vol. 22, no. 3, p. 668–674. DOI 10.1038/mt.2013.284. Available from: http://linkinghub.elsevier.com/retrieve/pii/S152500161631190X

7. MORRIS, Susan J, SEBASTIAN, Sarah, SPENCER, Alexandra J and GILBERT, Sarah C. Simian adenoviruses as vaccine vectors. *Future Virology* [online]. September 2016. Vol. 11, no. 9, p. 649–659. DOI 10.2217/fvl-2016-0070. Available from: https://www.futuremedicine.com/doi/10.2217/fvl-2016-0070

8. EWER, Katie, SEBASTIAN, Sarah, SPENCER, Alexandra J., et al. Chimpanzee adenoviral vectors as vaccines for outbreak pathogens. *Human Vaccines & Immunotherapeutics* [online]. 2 December 2017. Vol. 13, no. 12, p. 3020–3032. DOI 10.1080/21645515.2017.1383575. Available from: https://www.tandfonline.com/doi/full/10.1080/21645515.2017.1383575

9. ALTENBURG, Arwen, KREIJTZ, Joost, DE VRIES, Rory, et al. Modified Vaccinia Virus Ankara (MVA) as Production Platform for Vaccines against Influenza and Other Viral Respiratory Diseases. *Viruses* [online]. 17 July 2014. Vol. 6, no. 7, p. 2735–2761. DOI 10.3390/v6072735. Available from: http://www.mdpi.com/1999-4915/6/7/2735

10. COLLINS, Nicholas and BELKAID, Yasmine. Do the Microbiota Influence Vaccines and Protective Immunity to Pathogens? *Cold Spring Harbor Perspectives in Biology* [online]. February 2018. Vol. 10, no. 2, p. a028860. DOI 10.1101/cshperspect.a028860. Available from: http://cshperspectives.cshlp.org/lookup/doi/10.1101/cshperspect.a028860

11. ELOE-FADROSH, Emiley A, MCARTHUR, Monica A, SEEKATZ, Anna M, et al. Impact of oral typhoid vaccination on the human gut microbiota and correlations with s. Typhi-specific immunological responses. *PloS one* [online]. 2013. Vol. 8, no. 4, p. e62026. DOI 10.1371/journal.pone.0062026. Available from: http://www.ncbi.nlm.nih.gov/pubmed/23637957

12. OH, Jason Z, RAVINDRAN, Rajesh, CHASSAING, Benoit, et al. TLR5-mediated sensing of gut microbiota is necessary for antibody responses to seasonal influenza vaccination. *Immunity* [online]. 18 September 2014. Vol. 41, no. 3, p. 478–492. DOI 10.1016/j.immuni.2014.08.009. Available from: http://www.ncbi.nlm.nih.gov/pubmed/25220212

13. WILLIAMS, Wilton B, HAN, Qifeng and HAYNES, Barton F. Cross-reactivity of HIV vaccine responses and the microbiome. *Current opinion in HIV and AIDS* [online]. January 2018. Vol. 13, no. 1, p. 9–14. DOI 10.1097/COH.0000000000000423. Available from: http://www.ncbi.nlm.nih.gov/pubmed/29035947

14. KUKKONEN, Kaarina, NIEMINEN, Tea, POUSSA, Tuija, SAVILAHTI, Erkki and KUITUNEN, Mikael. Effect of probiotics on vaccine antibody responses in infancy--a randomized placebo-controlled double-blind trial. *Pediatric allergy and immunology : official publication of the European Society of Pediatric Allergy and Immunology* [online]. September 2006. Vol. 17, no. 6, p. 416–21. DOI 10.1111/j.1399-3038.2006.00420.x. Available from: http://www.ncbi.nlm.nih.gov/pubmed/16925686

15. SOH, Shu E, ONG, Dave Qi Rong, GEREZ, Irvin, et al. Effect of probiotic supplementation in the first 6 months of life on specific antibody responses to infant Hepatitis B vaccination. *Vaccine* [online]. 19 March 2010. Vol. 28, no. 14, p. 2577–9. DOI 10.1016/j.vaccine.2010.01.020. Available from: http://www.ncbi.nlm.nih.gov/pubmed/20105426

16. COLLOCA, S., BARNES, E., FOLGORI, A., et al. Vaccine Vectors Derived from a Large Collection of Simian Adenoviruses Induce Potent Cellular Immunity Across Multiple Species. *Science Translational Medicine* [online]. 4 January 2012. Vol. 4, no. 115, p. 115ra2-115ra2. DOI 10.1126/scitranslmed.3002925. Available from: http://stm.sciencemag.org/cgi/doi/10.1126/scitranslmed.3002925

17. TATSIS, N, TESEMA, L, ROBINSON, E R, et al. Chimpanzee-origin adenovirus vectors as vaccine carriers. *Gene therapy* [online]. March 2006. Vol. 13, no. 5, p. 421–9. DOI 10.1038/sj.gt.3302675. Available from: http://www.ncbi.nlm.nih.gov/pubmed/16319951

18. NWANEGBO, Edward, VARDAS, Eftyhia, GAO, Wentao, et al. Prevalence of neutralizing antibodies to adenoviral serotypes 5 and 35 in the adult populations of The Gambia, South Africa, and the United States. *Clinical and diagnostic laboratory immunology* [online]. March 2004. Vol. 11, no. 2, p. 351–7. Available from: http://www.ncbi.nlm.nih.gov/pubmed/15013987

19. DICKS, Matthew D J, SPENCER, Alexandra J, EDWARDS, Nick J, et al. A novel chimpanzee adenovirus vector with low human seroprevalence: improved systems for vector derivation and comparative immunogenicity. *PloS one* [online]. 2012. Vol. 7, no. 7, p. e40385. DOI 10.1371/journal.pone.0040385. Available from: http://www.ncbi.nlm.nih.gov/pubmed/22808149

20. ONDONDO, Beatrice, MURAKOSHI, Hayato, CLUTTON, Genevieve, et al. Novel Conserved-region T-cell Mosaic Vaccine With High Global HIV-1 Coverage Is Recognized by Protective Responses in Untreated Infection. *Molecular therapy : the journal of the American Society of Gene Therapy* [online]. April 2016. Vol. 24, no. 4, p. 832–42. DOI 10.1038/mt.2016.3. Available from: http://www.ncbi.nlm.nih.gov/pubmed/26743582

21. ANTROBUS, Richard D., COUGHLAN, Lynda, BERTHOUD, Tamara K., et al. Clinical assessment of a novel recombinant simian adenovirus ChAdOx1 as a vectored vaccine expressing conserved influenza a antigens. *Molecular Therapy*. 2014. Vol. 22, no. 3, p. 668–674. DOI 10.1038/mt.2013.284.

22. FISCHER, Will, PERKINS, Simon, THEILER, James, et al. Polyvalent vaccines for optimal coverage of

potential T-cell epitopes in global HIV-1 variants. *Nature medicine* [online]. January 2007. Vol. 13, no. 1, p. 100–6. DOI 10.1038/nm1461. Available from: http://www.ncbi.nlm.nih.gov/pubmed/17187074

23. MOTHE, Beatriz, LLANO, Anuska, IBARRONDO, Javier, et al. Definition of the viral targets of protective HIV-1-specific T cell responses. *Journal of translational medicine* [online]. 7 December 2011. Vol. 9, p. 208. DOI 10.1186/1479-5876-9-208. Available from: http://www.ncbi.nlm.nih.gov/pubmed/22152067

24. YANG, Otto O., ALI, Ayub, KASAHARA, Noriyuki, et al. Short Conserved Sequences of HIV-1 Are Highly Immunogenic and Shift Immunodominance. SILVESTRI, G. (ed.), *Journal of Virology* [online]. 15 January 2015. Vol. 89, no. 2, p. 1195–1204. DOI 10.1128/JVI.02370-14. Available from: http://jvi.asm.org/lookup/doi/10.1128/JVI.02370-14

25. WAHREN, B, DREXLER, I, SUTTER, G, HELLER, K and ERFLE, V. Highly attenuated modified vaccinia virus Ankara replicates in baby hamster kidney cells, a potential host for virus propagation, but not in various human transformed and primary cells. *Journal of General Virology* [online]. 1 February 1998. Vol. 79, no. 2, p. 347–352. DOI 10.1099/0022-1317-79-2-347. Available from: http://jgv.microbiologyresearch.org/content/journal/jgv/10.1099/0022-1317-79-2-347

26. MAYR, A, STICKL, H, MÜLLER, H K, DANNER, K and SINGER, H. [The smallpox vaccination strain MVA: marker, genetic structure, experience gained with the parenteral vaccination and behavior in organisms with a debilitated defence mechanism (author's transl)]. *Zentralblatt fur Bakteriologie, Parasitenkunde, Infektionskrankheiten und Hygiene. Erste Abteilung Originale. Reihe B: Hygiene, Betriebshygiene, praventive Medizin* [online]. December 1978. Vol. 167, no. 5–6, p. 375–90. Available from: http://www.ncbi.nlm.nih.gov/pubmed/219640

27. SUTTER, G and MOSS, B. Nonreplicating vaccinia vector efficiently expresses recombinant genes. *Proceedings of the National Academy of Sciences of the United States of America* [online]. 15 November 1992. Vol. 89, no. 22, p. 10847–51. Available from: http://www.ncbi.nlm.nih.gov/pubmed/1438287

28. HANKE, Tomás, MCMICHAEL, Andrew J, DENNIS, Michael J, et al. Biodistribution and persistence of an MVA-vectored candidate HIV vaccine in SIV-infected rhesus macaques and SCID mice. *Vaccine* [online]. 10 February 2005. Vol. 23, no. 12, p. 1507–14. DOI 10.1016/j.vaccine.2004.08.050. Available from: http://www.ncbi.nlm.nih.gov/pubmed/15670887

29. HANKE, T, MCMICHAEL, A J, SAMUEL, Rachel V, et al. Lack of toxicity and persistence in the mouse associated with administration of candidate DNA- and modified vaccinia virus Ankara (MVA)-based HIV vaccines for Kenya. *Vaccine* [online]. 22 November 2002. Vol. 21, no. 1–2, p. 108–14. Available from: http://www.ncbi.nlm.nih.gov/pubmed/12443668

30. ONDONDO, Beatrice, BRENNAN, Caroline, NICOSIA, Alfredo, CROME, Steven J. and HANKE, Tomáš. Absence of systemic toxicity changes following intramuscular administration of novel pSG2.HIVconsv DNA, ChAdV63.HIVconsv and MVA.HIVconsv vaccines to BALB/c mice. *Vaccine*. 2013. Vol. 31, no. 47, p. 5594–5601. DOI 10.1016/j.vaccine.2013.06.068.

31. BORTHWICK, Nicola, AHMED, Tina, ONDONDO, Beatrice, et al. Vaccine-elicited Human T Cells Recognizing Conserved Protein Regions Inhibit HIV-1. *Molecular Therapy* [online]. February 2014. Vol. 22, no. 2, p. 464–475. DOI 10.1038/mt.2013.248. Available from: http://linkinghub.elsevier.com/retrieve/pii/S1525001616311674

32. BORTHWICK, Nicola, LIN, Zhansong, AKAHOSHI, Tomohiro, et al. Novel, in-natural-infection subdominant HIV-1 CD8+ T-cell epitopes revealed in human recipients of conserved-region T-cell vaccines. *PloS one* [online]. 2017. Vol. 12, no. 4, p. e0176418. DOI 10.1371/journal.pone.0176418.

Available from: http://www.ncbi.nlm.nih.gov/pubmed/28448594

33. HANCOCK, Gemma, MORÓN-LÓPEZ, Sara, KOPYCINSKI, Jakub, et al. Evaluation of the immunogenicity and impact on the latent HIV-1 reservoir of a conserved region vaccine, MVA.HIVconsv, in antiretroviral therapy-treated subjects. *Journal of the International AIDS Society* [online]. 2017. Vol. 20, no. 1, p. 21171. DOI 10.7448/IAS.20.1.21171. Available from: http://doi.wiley.com/10.7448/IAS.20.1.21171

34. HAYTON, Emma-Jo, ROSE, Annie, IBRAHIMSA, Umar, et al. Safety and Tolerability of Conserved Region Vaccines Vectored by Plasmid DNA, Simian Adenovirus and Modified Vaccinia Virus Ankara Administered to Human Immunodeficiency Virus Type 1-Uninfected Adults in a Randomized, Single-Blind Phase I Trial. GOEPFERT, Paul A. (ed.), *PLoS ONE* [online]. 9 July 2014. Vol. 9, no. 7, p. e101591. DOI 10.1371/journal.pone.0101591. Available from: http://dx.plos.org/10.1371/journal.pone.0101591

35. MOTHE B, MANZARDO C, SNACHEZ-BERNABEAU A, COLL P, MORON S, PEURTAS MC, et al. Therapeutic ChAdV63.HIVconsv-MVA.HIVconsv vaccination refocused T cells to conserved regions of HIV in early reated HIV-1 infected individuals. *Submitted*.

36. MOTHE B, MOLTÓ J, MANZARDO C, COLL J, PUERTAS MC, MARTINEZ-PICADO J, et al. Viral control induced by HIVconsv vaccines & Romidepsin in early treated individuals. In : *The Conference on Retroviruses and Opportunistic Infections; Seattle, WA, USA*. 2017.

37. MUTUA, Gaudensia, FARAH, Bashir, LANGAT, Robert, et al. Broad HIV-1 inhibition in vitro by vaccine-elicited CD8+ T cells in African adults. *Molecular Therapy - Methods & Clinical Development* [online]. 2016. Vol. 3, p. 16061. DOI 10.1038/mtm.2016.61. Available from: http://linkinghub.elsevier.com/retrieve/pii/S2329050117300311

38. LÉTOURNEAU, Sven, IM, Eung-Jun, MASHISHI, Tumelo, et al. Design and pre-clinical evaluation of a universal HIV-1 vaccine. *PloS one* [online]. 3 October 2007. Vol. 2, no. 10, p. e984. DOI 10.1371/journal.pone.0000984. Available from: http://www.ncbi.nlm.nih.gov/pubmed/17912361

39. ZOU, Chengcheng, MURAKOSHI, Hayato, KUSE, Nozomi, et al. Effective Suppression of HIV-1 Replication by Cytotoxic T Lymphocytes Specific for Pol Epitopes in Conserved Mosaic Vaccine Immunogens. *Journal of virology* [online]. 1 April 2019. Vol. 93, no. 7. DOI 10.1128/JVI.02142-18. Available from: http://www.ncbi.nlm.nih.gov/pubmed/30674626

40. BADEN, Lindsey R, KARITA, Etienne, MUTUA, Gaudensia, et al. Assessment of the Safety and Immunogenicity of 2 Novel Vaccine Platforms for HIV-1 Prevention: A Randomized Trial. *Annals of internal medicine* [online]. 1 March 2016. Vol. 164, no. 5, p. 313–22. DOI 10.7326/M15-0880. Available from: http://www.ncbi.nlm.nih.gov/pubmed/26833336

24 APPENDIX A: SCHEDULE OF PROCEDURES

**Procedure / Study week**	**Screen**	**0**	**1**	**2**[4]	**4**	**5**	**6**	**8**	**12**	**16**	**30**	**44**	**48**[3]
**Study day**	**-28**	**0**	**7**	**14**	**28**	**35**	**42**	**56**	**84**	**112**	**210**	**308**	**336**
Visit windows (days)	(≤ -28)	0	±3	±3	±3	±3	±3	±3	±7	±7	±7	±7	±7
Informed consent	X												
Confirm eligibility	X	X											
Randomisation		X											
**VACCINATIONS**													
ChAdOx1.tHIVconsv1 or Placebo		X											
MVA.tHIVconsv3 and MVA.tHIVconsv4 or Placebo					X								
**COUNSELLING**													
HIV Risk Reduction Counselling	X	X			X			X		X	X	X	X
HIV Test Counselling	X	X			X			X		X	X	X	X
Family Planning Counselling	X	X			X			X	X	X	X	X	X
Social Impact Assessment													X
**CLINICAL ASSESSMENTS**													
HIV-1 Risk Assessment	X	X			X			X	X	X	X	X	X
Comprehensive Medical History	X												
Interim Medical History		X	X	X	X	X	X	X	X	X	X	X	X
General Physical Exam	X	X			X								X
Symptom Directed Physical Exam			X			X	X	X	X	X	X	X	
Weight	X												X
Height	X												

Procedure / Study week	Screen	0	1	2[4]	4	5	6	8	12	16	30	44	48[3]
**Study day**	**-28**	**0**	**7**	**14**	**28**	**35**	**42**	**56**	**84**	**112**	**210**	**308**	**336**
Visit windows (days)	(≤ -28)	0	±3	±3	±3	±3	±3	±3	±7	±7	±7	±7	±7
Vital signs	X	X[2]	X		X[2]	X	X	X	X	X	X	X	X
Cervical & Axillary lymph nodes		X	X		X	X							
Adverse Events		X	X	X	X	X	X	X					
Serious Adverse Events		X	X	X	X	X	X	X	X	X	X	X	X
Concomitant Medications	X	X	X	X	X	X	X	X					
Local and Systemic Reactogenicity Assessment[1]		X[2]	X		X[2]	X							
**LABORATORY PROCEDURES**													
HBsAg, HCV Antibodies,	X												
Syphilis, *Chlamydia trachomatis* and *Neisseria gonorrhoeae*	X							X		X	X		X
HIV-testing	X	X			X			X		X	X	X	X
Urinalysis	X							X				X	
Routine Haematology	X	X	X		X	X		X			X		X
Biochemistry	X	X	X		X	X		X					
Pregnancy Test (if applicable)	X	X			X			X		X			X
Faecal samples[5]		X[6]				X							X
**IMMUNOLOGY**													
HLA Typing		X											
Fresh ELISpots		X				X						X	
PBMC, serum and plasma storage (frozen ELIspot, VIA, ICS, memory)		X				X	X					X	

Procedure / Study week	Screen	0	1	2[4]	4	5	6	8	12	16	30	44	48[3]
**Study day**	**-28**	**0**	**7**	**14**	**28**	**35**	**42**	**56**	**84**	**112**	**210**	**308**	**336**
Visit windows (days)	(≤ -28)	0	±3	±3	±3	±3	±3	±3	±7	±7	±7	±7	±7
Epitope Mapping								X	X				

[1]Reactogenicity will be collected from Day 0 to Day 7 after each vaccination.

[2]Collect at baseline and at least 30 min post vaccination

[3] Early Termination (ET): Procedures to be performed at ET are the same as the Week 48 visit procedures.

[4] Visit can be conducted by phone. The visit may also be conducted in the clinic.

[5] Faecal sample collection will be optional

[6] Faecal sample should be collected before administration of the first vaccine dose at enrollment

25 APPENDIX B: AMENDMENT HISTORY

Amendment No.	Protocol Version No.	Date issued	Author(s) of changes	Details of Changes made

List details of all protocol amendments here whenever a new version of the protocol is produced.

Protocol amendments must be submitted to the Sponsor for approval prior to submission to the REC committee or MHRA.

26 APPENDIX C: ADVERSE EVENT SEVERITY ASSESSMENT TABLE

The Division of AIDS (DAIDS) Table for Grading the Severity of Adult and Pediatric Adverse Events. Version 2.1 July 2017.

https://rsc.niaid.nih.gov/sites/default/files/daidsgradingcorrectedv21.pdf (accessed 28th July 2019).
